# Why are the implementation of and access to child developmental assessment within routine healthcare so difficult in Ethiopia? A descriptive qualitative study

**DOI:** 10.1371/journal.pone.0355686

**Published:** 2026-08-10

**Authors:** Selamawit Gebeyehu, Monique Mommers, Sultan Hussen, Mark Spigt

**Affiliations:** 1 School of Public Health, Arbaminch University, Arbaminch, Ethiopia; 2 Department of Epidemiology, CAPHRI School for Public Health and Primary Care, Maastricht University, Maastricht, Netherlands; 3 Department of Family Medicine, CAPHRI School for Public Health and Primary Care, Maastricht University, Maastricht, Netherlands; Curtin University, AUSTRALIA

## Abstract

**Introduction:**

Developmental delays in early childhood present significant long–term challenges, making early developmental assessment crucial for effective intervention. Despite this, such assessments are not routinely implemented within routine healthcare services in Ethiopia. This study examines the barriers and facilitators affecting both the provision and accessibility of developmental assessments by exploring the experiences of health professionals and parents of under–five children.

**Methods:**

A descriptive qualitative study was conducted in the Gamo Zone, South Ethiopia, between August 2023 and January 2024. Twenty semi‑structured Key Informant Interviews (KII) were undertaken with purposively selected key informants, including one zonal maternal and child health (MCH) focal person, two MCH officers, two MCH coordinators, two pediatricians, three pediatric nurses, four general practitioners, and six parents of children under five years of age. All interviews were audio‑recorded, transcribed verbatim, and analyzed using ATLAS.ti 7. A thematic analysis approach was applied to identify barriers and facilitators influencing the provision and accessibility of child developmental assessments.

**Findings:**

Health professionals reported challenges that hindered the delivery of effective developmental assessments, including poor infrastructure, limited government commitment, funding shortages, and insufficient knowledge and technical skills regarding developmental delays and assessment procedures. Conversely, the structured layout of the existing health‑care system and an established referral pathway were reported as operational facilitators to support the provision of these services. Parents described barriers to accessing developmental assessments, including low socioeconomic status, prevailing community beliefs, societal attitudes, and stigma toward children with developmental delays. Encouragingly, broad community trust in and acceptance of routine child health programs emerged as an important facilitator that improved access to developmental assessment services.

**Conclusion:**

Improving the integration of developmental assessment into Ethiopia’s routine child health services requires addressing both system‑level shortcomings and the social realities that shape families’ access to care. A practical, implementation‑focused approach, one that strengthens frontline capacity, reinforces existing health‑system structures, and builds on community trust, offers the most promising path forward. With coordinated commitment, developmental assessment can shift from a neglected service to a reliable and sustainable part of pediatric care.

## Introduction

Early childhood is widely regarded as the most critical stage of development, forming the foundation for success and well–being throughout the lifespan [1,2]. Unfortunately, not every child has the opportunity to develop to their full potential; developmental disabilities are, in fact, the leading cause of childhood disability worldwide. In 2016, the Global Burden of Disease (GBD) study estimated that 52.9 million children globally (8.4%) were affected by one of six developmental disabilities: intellectual disability, attention–deficit/hyperactivity disorder (ADHD), autism spectrum disorder (ASD), epilepsy, hearing loss, or vision loss [3]. The majority of children with developmental disabilities reside in low–and middle–income countries (LMICs). Nigeria, the Democratic Republic of the Congo, and Ethiopia are among the top 10 African countries with the largest burden of pediatric developmental disabilities [2–8].

In response, the World Health Organization (WHO) and other global organizations have launched initiatives to improve early childhood development worldwide, including in Ethiopia [9]. Programs such as the Healthy Child Program (HCP), the Nurturing Care Program, and the Care for Child Development program have been implemented [9,10]. The programs acknowledge early identification of developmental problems is an essential first step toward ensuring that children receive the necessary interventions and support to promote optimal growth and development [7,8,11].

While developmental delay is a significant public health issue, its identification through early evaluation remains suboptimal, even in high–income countries [7,12]. A multi–country review highlighting this variability showed that the uptake of developmental checks fluctuates significantly by age and region: in Australia, 67.5% of children received checks at 12 months, and 61% at 3.5 years; in New Zealand, 68% received all core contacts by 12 months, with 89% receiving checks at 4 years. Sweden reports 89%–95% coverage for language screening at 2.5 years, while Canada reports 54% at 18 months. In the United Kingdom, 87% coverage was achieved at 12 months, and 78% at 2.5 years [12].

In LMICs, current detection rates of developmental disorders are lower than their actual prevalence. This discrepancy is compounded by a lack of data on attendance rates for developmental assessments, a gap that further highlights the persistent challenges in early identification [13]. Evidence from LMICs shows that most primary care providers utilize a flexible, continuous strategy to monitor a child’s developmental progress. In these settings, knowledgeable professionals conduct skilled observations, which include addressing parental concerns, obtaining a relevant developmental history, making accurate and informative observations, and sharing insights with other professionals [14,15].

In Ethiopia, developmental assessments are conducted by healthcare providers primarily in response to parental concerns or after identifying risk factors in the child. A study in Addis Ababa revealed that only 28.6% of healthcare providers assess children’s developmental milestones, and just 3.7% use a standardized developmental screening tool as a reference [16]. Among the respondents, 18.5% classify children for developmental delay and 18.1% use a clinical diagnosis classification system. Only 0.8% apply the Integrated Management of Neonatal and Childhood Illness (IMNCI) classification system for developmental delay. This low rate of assessment is attributed to the lack of referral forms, the absence of developmental milestone assessment tools, and limited provider knowledge [16].

Programmatically, most LMICs, including Ethiopia, rely on ‘opportunistic’ screening during immunization or illness visits, as these are the most consistent health touchpoints [9,17]. For instance, a study conducted in Saudi Arabia found that parents primarily brought their children to health facilities for illness or immunization visits, with routine checkups focused on height, growth, and vaccination [18]. Similarly, an Ethiopian study reported that only 58.6% of healthcare providers conducted developmental assessments during well–child and sick–child visits [16]. However, despite the strategic advantages of this integration, significant gaps remain. The existing literature does not fully explain the systemic or personal barriers that cause providers to miss these opportunistic assessments even when a child is present. Furthermore, this facility–based model inherently overlooks children who cannot access clinics due to structural barriers, similar to those identified in high-income contexts like Australia, such as limited transportation, language barriers, lack of awareness, misunderstanding of the importance of screening, and families’ social circumstances [19].

While the prevalence of developmental delay in sub–Saharan Africa is well–documented [2–8], data on the actual utilization of developmental monitoring services remains critically scarce. Unlike high–income settings where utilization rates are consistently tracked [12], evidence from regional neighbors like Kenya suggests that service uptake is hindered by a lack of awareness, with only 2.6% of caregivers knowing that developmental monitoring is available [20]. Even where integrated policies exist such as in South Africa, high patient volumes prevent many at–risk children from being reached [21]. Considering that research on these specific barriers in Ethiopia is limited, this study examines the challenges faced by health professionals and parents in delivering or accessing these assessments.

## Methods and materials

### Study area and period

This study was conducted in the Gamo Zone, Southern Ethiopia, of which Arbaminch is the administrative center. The zone comprises 20 districts, including 14 rural and six urban districts. As of July 2024, the total population of the Zone was estimated at 2,391,628 (1,185,044 males and 1,206,584 females). The population is predominantly rural, with 1,927,891 residents (80.6%) living in rural areas compared to 463,737 (19.4%) in urban centers. Crucially, children under five years of age, the target population for this study, accounted for approximately 368,310 (15.4%) of the total zonal population [22].

The Ethiopian health service operates within a three–tier system, with primary, secondary, and tertiary levels of care. At the primary level, services are provided by primary hospitals, health centers, and health posts. A primary hospital serves an average population of 100,000 people, offering both inpatient and ambulatory services. The Primary Health Care Unit (PHCU) includes a referral health center and five satellite health posts, which are the lowest–level health facilities, at the village level [23].

As of December 2025, Gamo Zone had 61 health centers, nine hospitals (one general hospital and eight primary hospitals), 17 clinics and 272 health posts providing pediatric services for children under five. Developmental assessments are currently conducted as clinical evaluations when clinicians suspect developmental delays, using the IMNCI guideline [24]. Growth monitoring for under–five children is available at hospitals, health centers, and through community–based outreach activities. The present study was conducted from August 1, 2023 to January 1, 2024.

### Study design and population

This study employed a qualitative descriptive design to provide a comprehensive overview of the developmental assessment implementation status. By integrating the lived experiences of parents and frontline clinicians with the systemic perspectives of MCH officers and coordinators, the research captured the entire implementation chain, from policy–level supervision to point–of–care delivery. A total of 20 Key informants were purposively selected from zonal and district MCH officers, facility–level MCH coordinators, and a range of clinical staff, including pediatricians, general practitioners (GPs), nurses, and health officers working in under–five outpatient departments (OPDs), alongside parents from selected districts.

Because administrative roles at the zonal and district levels consist of single–occupant positions, a total population sampling approach was used for these specific officials to ensure comprehensive representation. Their inclusion was critical for exploring system–level factors since they are responsible for the coordination, supervision, and monitoring of child health programs within routine healthcare services. For clinical and parent groups, participants were selected based on their “information–rich” perspectives. In the absence of a formal zonal registry or published prevalence data for developmental delays, mothers were recruited through the guidance of Health Extension Workers (HEWs). Acting as community gatekeepers, HEWs identified mothers who could effectively explain the service delivery based on their experience navigating under–five OPDs and their involvement in community health activities.

To ensure a comparative perspective on universal assessment, the principle that every child should receive developmental monitoring regardless of their apparent developmental status [25], the parent group included four mothers raising both typically developing children and children with developmental delays, and two mothers of typically developing children. This composition allowed the study to determine whether developmental assessment occurs during routine “well–child” visits or only when a deficit is already suspected. Data saturation was assessed iteratively through a constant comparative method. While the administrative sample was limited by nature, thematic redundancy across the clinician and parent groups was reached when successive interviews yielded repetitive information. For the parent group, saturation was achieved by the sixth interview, as narratives regarding facility–level gaps, social stigma, and referral challenges became highly consistent, confirming the adequacy and depth of the findings.

### Data collection instrument and procedure

Semi–structured guides were utilized for the KIIs (S1 File). Separate interview guides were developed for each participant group, informed by expert opinions, investigator expertise, and a review of relevant literature. Following the first interview with each group, the guides were revised, and questions were refined in the final version to better explore issues related to developmental delays. The interview guides covered topics such as socio–demographic characteristics, concepts of child health and development, the current status of developmental assessments, and barriers and facilitators within the health system, among professionals, communities, and parents. Additionally, they included questions on perceptions of using standardized screening tools and related recommendations. Questions were open–ended and supported by probes to encourage detailed responses. The guide was initially drafted in English, translated into Amharic, and refined following the first few interviews.

The KIIs were conducted by three interviewers, including the first author. All of whom held at least a master’s degree in a health–related discipline and had prior qualitative data collection experience. To ensure rigor and minimize bias, the first author oversaw the standardization of the interview guide and protocol. While the first author participated in data collection to ensure thematic depth, the other two data collectors, who were not involved in the initial study design, conducted separate interviews to provide a diverse range of participant perspectives. Study participants were identified through collaboration with the Zonal Health Bureau, hospitals, health centers, and HEWs. Written informed consent was obtained from all willing participants. Interviews were scheduled and conducted at participants’ workplaces or in open village spaces, ensuring a comfortable setting. The interviews were conducted face–to–face in the local language, and lasted approximately 30 minutes. All sessions were professionally audio–recorded, with key points documented in a notebook.

### Trustworthiness

Data were collected by trained and experienced interviewers who addressed challenges in consultation with the research team. A well–established, expert–reviewed interview guide was utilized, with investigators maintaining a neutral stance and encouraging participants to provide detailed responses. Credibility and trustworthiness were enhanced by engaging key informants with diverse experiences and backgrounds and by employing data triangulation. The findings were supported by direct quotes from participants. Transcripts, which are verbatim written records of interviews, were carefully created to preserve the exact wording of participants. Translations were also made into English, ensuring that the meaning and tone of participants’ words were accurately shared. Demographic summaries were carefully maintained to provide context for the findings.

### Researcher reflexivity

Throughout this study, our team engaged in ongoing reflexivity to consider how our backgrounds, assumptions, and ethical responsibilities shaped the research process. Although none of the authors had direct experience delivering developmental assessment services, we brought varied experiences in child development and health systems research. These perspectives guided our early thinking, but as the study progressed, we learned that some of our assumptions were not accurate; for example, we expected that health facilities would have guidelines for assessing developmental delay, yet in reality no such guidelines existed in some health facilities, and many child health services were delivered together with maternal services.

We were attentive to power dynamics during interviews and ensured that data were collected by the first author and other data collectors who had no supervisory relationships with participants, particularly higher–level officials and health service providers. We also recognized that parents might feel restricted when speaking with unfamiliar researchers; therefore, we clearly explained the aim of the study and worked to create an open, comfortable environment for discussion.

Reflexivity was further supported through the first author’s reflexive journal and regular team debriefings, where we discussed emerging impressions, questioned potential biases, and considered how organizational policies and system constraints influenced participants’ experiences. These reflections helped ground our analysis in participants’ perspectives, and the process has deepened our understanding of developmental assessment and will continue to shape our future work.

### Data processing and analysis

Audio–recorded interviews were transcribed verbatim and translated into English, with all participant identifiers removed (S2 File). Thematic analysis was employed to explore barriers and facilitators to child developmental assessment [26,27]. Transcriptions were coded using ATLAS. ti 7 and categorized into sub–themes. Key findings were presented based on emergent themes and discussed in the context of their relevance. Themes were cross–checked against transcripts and substantiated with direct quotes from participants to ensure accuracy.

Following the identification of key themes through thematic analysis, the findings were further examined using the Consolidated Framework for Implementation Research (CFIR). This framework was selected to provide a structured approach to understanding the implementation of developmental assessment practices. CFIR’s five domains: Intervention Characteristics, Outer Setting, Inner Setting, Characteristics of Individuals, and Process, offered a comprehensive lens for interpreting the barriers and facilitators identified in the data [28]. The framework was applied retrospectively; it was not used to guide data collection or the development of interview guides. Instead, it was employed after thematic analysis to align and interpret the emergent themes within an established implementation science framework.

### Ethical considerations

Ethical approval for the study was granted by the Institutional Review Board (IRB) of Arbaminch University, College of Medicine and Health Sciences with reference number IRB/1327/2022. A formal letter of support was obtained from the research coordination office. Participants were verbally informed about the study’s purpose before the interviews. The study posed no risks to participants, who retained the right to withdraw without consequences. Written and oral (audio–recorded) consent was obtained after providing a detailed explanation of the study. Confidentiality and anonymity were assured, and data were exclusively used for research purposes.

## Results

Twenty KIIs were conducted: fourteen with primary healthcare professionals and six with parents of children under five. Among the parents, four had children with developmental delays and two had children with typical development. Of the health‑care providers, eleven were male and three were female. Regarding educational attainment, two participants held a specialization in pediatrics, two held master’s degrees, and ten held bachelor’s degrees, which included the general practitioners (Table 1).

**Table 1:** Socio–demographics and professional characteristics of participants in key informant interviews, separately for health care providers and parents (mothers).

Characteristics		N	%
Health care providers (n = 14)			
Professional role	MNCH focal person (Zonal/Woreda/HC)	5	35.7
	Pediatricians	2	14.3
	Pediatric nurses/ Health officers	3	21.4
	General practitioners (GP)	4	28.6
Age (years)	20–30	6	42.8
	>30	8	57.2
Gender	Male	11	78.6
	Female	3	21.4
Years of experience	<5 years	4	28.6
	≥5 year	10	71.4
Educational status	Bachelor’s degree (including GP)	10	71.4
	Master’s degree	2	14.3
	Specialization (Pediatrician)	2	14.3
Parents (n = 6)			
Age (years)	20–30	3	50.0
	>30	3	50.0
Gender of the child	Male	2	33.3
	Female	4	66.6
Highest educational status	No formal education	2	33.3
	Primary	3	50.0
	Secondary and above	1	16.7
Parity	Primiparous	2	33.3
	Multiparous	4	66.6

**Note:** In Ethiopia, a Zone (Zonal) is an administrative division within a region, a Woreda is a district within a zone, and a Kebele is the smallest administrative unit within a woreda.

### Overview of the current developmental assessment practice

All healthcare providers highlighted that current developmental assessment services are inadequate and insufficient. They noted limited stakeholder involvement and reported that these services receive far less attention than other child‑health activities. Providers also explained that developmental assessments are offered only to children with identified risk factors rather than being accessible to all children.

*“The service being provided currently is not sufficient. The children come to the health facility by coincidence... it has not received attention like other diseases.”* (BSc nurse working at an under–five OPD in a health center)

*“We mainly see children who have concerns, especially those who are malnourished, and we do something special for them… If a child fails to meet expectations, we provide special supervision.”* (BSc nurse working at an under–five OPD in a health center)

#### Facilitators and barriers of child developmental assessment.

**Fig 1:**
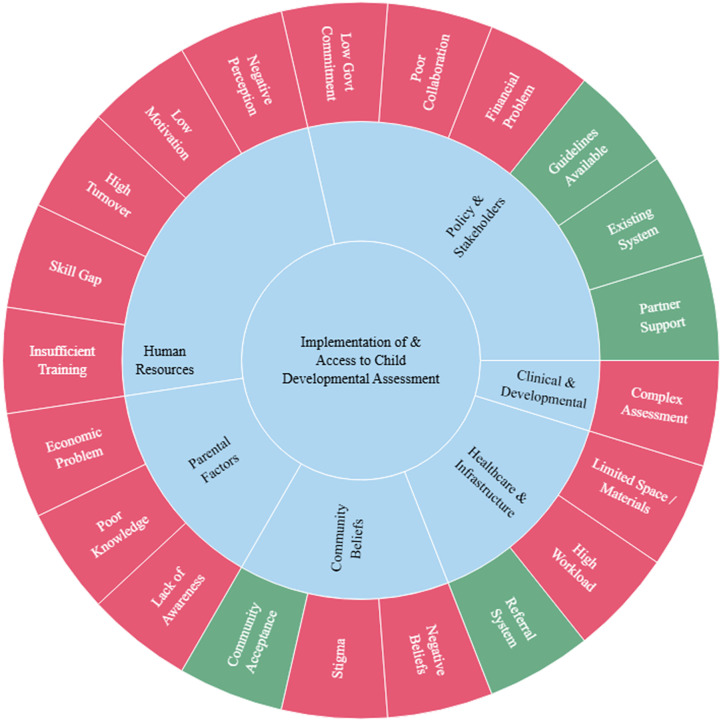
Themes and sub–themes of the barriers to and facilitators of assessment. Note: The sunburst diagram illustrates the multi–level factors influencing the implementation of and access to child developmental assessment. Red: Barriers; Green: Facilitators; Light Blue: Thematic domains.

We identified a range of facilitators and barriers influencing the implementation of child developmental assessment within routine health‑care services, organized according to the CFIR framework. These themes spanned four key domains: the outer setting, which included policy, government and stakeholder commitment and attention; the inner setting, which encompassed health‑care system capacity and infrastructure; characteristics of individuals, particularly human‑resource factors; and intervention characteristics, relating to the nature and design of the assessment. Additional influences emerged from community beliefs and attitudes, as well as parental knowledge, attitudes, and circumstances, all of which shaped how developmental assessment services were delivered and accessed (Fig 1).

### Theme 1: Policy, government, and stakeholder commitment and attention

This theme maps to the outer setting domain of the CFIR. Almost all participants described child developmental delay as a low–priority public health issue, with limited political commitment, weak multisectoral coordination, and insufficient financial allocation. National child health priorities in Ethiopia favor high prevalence infectious diseases (like diarrhea, malaria, and pneumonia), resulting in developmental delays being undervalued as a public health concern. This lack of top–level prioritization directly restricts budget allocations, resulting in shortages of essential materials, dedicated infrastructure and provider training. Structurally, while child development is recognized as a multifaceted issue, actual multisectoral stakeholder collaboration remains limited.

Participants consistently reported that developmental delay is not considered a major public health priority, resulting in weak policy attention and limited system–level advocacy.

*“…the problem has got less concern and it***’***s not considered as a public health priority. This may be due to the rare nature of the problem and it did not get that much concern. I think this might continue until someone starts to advocate about it.”* (General practitioner working at a primary hospital)

Providers emphasize that without political will and dedicated financing, identifying developmental delays will remain a peripheral task.

*“There is a lack of political attention at the top level, coupled with gaps in knowledge and skills at the provider level… Political commitment is crucial; if there is political will, the necessary structures will be implemented across health facilities.”* (Pediatrician)

Participants highlighted national initiatives introduced three years ago, such as the early identification of developmental delays as part of national strategies and using the prevalence of developmental disabilities as an indicator of child development, as key facilitators for providing developmental assessment. These initiatives have driven the adoption of updated IMNCI guidelines, which now incorporate developmental assessment as a component and emphasize reporting the number of children with developmental delays alongside other efforts to improve early childhood development.

*“Training was provided, integrated with IRT training for HEWs. Currently, HEWs use the ICMNCI guidelines, formerly known as integrated community case management (ICCM), to screen and classify children. For those aged 2 to 5 years, children are classified as having confirmed developmental delay or no developmental delay.”* (Child health focal person)

External partners were also described as supporting implementation efforts.

*“Another aspect is the presence of various stakeholders... The World Food Program and UNICEF have been working with the government to prevent and address developmental delays.”* (MCH focal person)

### Theme 2: Healthcare system and infrastructure

This theme maps to the inner setting domain of the CFIR. Participants emphasized that having an appropriate workspace, a dedicated room, and adequate supplies is crucial for providing developmental assessments. However, even when staff want to check a child, the physical working environment makes it difficult. A proper developmental checkup requires a calm space where a health worker can watch a child play, move, or speak. However, clinics are severely overcrowded. Most checkups happen in tiny, shared rooms where multiple activities are going on at the exact same time. There is no privacy for families, no physical space to test a child’s motor skills, and not even shelves to store testing materials securely.

The process of conducting developmental assessments also relies on the availability of a guide or screening tool. Unfortunately, in some health facilities, the national IMNCI guidelines are unavailable. Participants highlighted that the absence of standard tools hampers their ability to deliver consistent services, leading to significant variations in how developmental assessments are conducted across providers.

*“The setup is not suitable... expanded program on immunization (EPI) services are provided alongside developmental assessments. Regional officials brought some items, but we don’t even have a shelf to store them. It’s a real challenge to do it correctly*.” (Female BSc nurse working at under–five OPD at health center)

“*It is by looking and asking the mother, there is no manual other than that. I have neither been trained on nor experienced the new IMNCI guide, the one I am applying is the old one. I use it.*” (Female BSc nurse working at under–five OPD at health center)

Most healthcare providers agree that the current referral system is highly effective in identifying children with developmental delays. In Ethiopia, healthcare services follow a three–tier referral system, where health services are delivered at health posts, health centers, general hospitals, and specialized hospitals. Community health workers, particularly the HEWs, routinely visit households and refer any children suspected of illness for further evaluation at a higher health care level. In addition, they continue to follow up with the child after referral. This system improves service delivery for parents by making it more accessible and efficient, while also enhancing the quality of care through connections with experts who have the necessary knowledge and skills. They also recommend integration of the services with the existing child health services.

*“We provide follow–up and professional support... If we are unable to address the issue within our setup, we refer the child to other settings for additional support.”* (Key informant, public health officer)

*“To ensure continuous monitoring, for example, children who come for EPI services receive immunization whether or not they are sick. This allows us to follow up even after the children complete their immunization schedule.”* (Health officer at the health center)

This mismatch between available entry points and physical capacity highlights a major contradiction in how the system is designed to deliver developmental assessment. On one hand, participants mentioned that the healthcare system has a well–structured referral path, where local village HEWs can find sick children at home and refer them up to health centers and hospitals. Furthermore, the health facilities have established entry points, such as routine vaccine OPDs (EPI), where healthy children visit regularly. On the other hand, participants highlighted that the vaccine rooms and child OPDs used for developmental assessments are already crowded, loud, and occupied, presenting a structural barrier to service delivery.

### Theme 3: Human Resources

This theme spans the characteristics of individuals and inner setting domains of the CFIR. Participants described the clinical implementation of developmental assessment as heavily bottlenecked by individual provider attributes and acute systematic constraints within the facility environment. Frontline health workers face a heavy burden that undermines their motivation to do developmental assessments. First, their training was far too basic, feeling more like a quick orientation than a comprehensive education. This leaves them with major gaps in their clinical skills, making them feel insecure and hesitant due to the fear of making the incorrect diagnosis. Second, this low confidence is exacerbated by unfavorable health facility conditions, including low staff numbers, high turnover rates, and high workloads. It is common for a single worker to see up to 50 sick children per day at times. Because a high–quality developmental assessment can require up to an hour of patience and careful observation, it becomes physically impossible to do during a rushed day. Consequently, healthcare workers choose to spend their limited time treating acute infections that could be fatal within days, rather than tracking a developmental delay that appears less urgent.

*“I don’t think I have enough knowledge about developmental assessments… I’m not confident in making decisions regarding developmental problems.”* (B.Sc. nurse at health center)

*“One issue is the motivation of healthcare providers, since developmental assessments are time–consuming and require patience. Provider ignorance, possibly due to lack of training and updates, contributes to this issue.”* (GP at health center)

*“Regarding developmental issues, professionals often do not give them much attention, possibly due to a lack of awareness or not perceiving them as significant problems. Even when they do pay attention, they often do not take the time to thoroughly examine the child.”* (GP at primary hospital)

Parents experience this combination of provider under–training, limited knowledge and high workload as an absolute barrier to care, noting that developmental concerns are frequently minimized or actively dismissed during routine consultations.

*“Health professionals initially said it wasn’t a problem, so how can I say it is?... Is it reasonable to think that I know better than the health professionals?”* (Mother of a child with a gross motor problem who cannot walk)

### Theme 4: Clinical and assessment–related factors

This theme maps to intervention characteristics domain of CFIR. Most participants highlighted that the complex nature of developmental problems, acts as a prominent barrier to implementation in primary healthcare settings. Developmental assessment has features of high complexity due to the multi–causal, non–acute nature of developmental delays. Accurately assessing these conditions requires well–trained personnel, multidisciplinary teams, and specialized diagnostic materials that are missing at primary care tier.

Consequently, managing a suspected delay needs a resource–intensive diagnostic pathway involving extensive laboratory investigations and multi–stage referrals to higher levels of healthcare. This introduces a high-cost barrier that places an unsustainable financial burden on both the public health system and individual families.

Frontline physicians emphasize that the immense economic and infrastructural requirements of developmental testing isolate it from simpler, low–cost clinical services.

“*Treating developmental delays requires a multidisciplinary team, including physiotherapists, speech therapists, and pediatric neurologists.”* (Pediatrician)

“… *for developmental assessment, the system is poor related to economic issues. It needs a huge economy. In the other conditions with a little expense, you can do so many things but for this, it needs a big expense to provide the service.”* (GP at primary hospital**)**

### Theme 5: Community norms, attitudes, and belief–related barriers

This theme maps to the outer setting domain of the CFIR. According to providers and parents, developmental assessment implementation and access are moderated by the outer setting, specifically the cultural norms and traditional belief systems that shape patient needs and resources. Deep–seated community stigmas undermine formal healthcare–seeking behaviors. Developmental delays are frequently attributed to supernatural causes, ancestral curses, or evil spirits, which prompts parents to seek initial interventions from traditional or spiritual healers rather than formal medical clinics.

This delay is compounded by widespread cultural variations in milestone interpretation, such as normalizing delays as harmless, temporary family traits. Furthermore, fear of community stigmatization and the social shame associated with a perceived “family curse” frequently drive families to isolate and hide their children at home, preventing early interaction with healthcare providers.

Healthcare providers note that sociocultural explanations of disability drive families away from clinical settings, causing children to present to facilities only after severe medical complications emerge.

*“The community holds beliefs associating developmental delay with demons or evil spirits, leading them to believe that only spiritual intervention can help. This attitude is reinforced by key individuals in the community who advise parents to wait and see if the child improves on their own, suggesting that taking the child to a health facility won’t make a difference at that time. It’s largely an attitude problem. Parents often bring their children to health facilities only when other approaches fail, seeking medical treatment as a last resort.” (GP at primary hospital)*

*“The community prefers to keep cases of developmental delay private. Families usually only seek help when their child develops additional issues like diarrhea or fever. Typically, only a few educated family members pursue services for developmental delays. Most families isolate children with developmental delays socially, keeping them hidden at home. ” (Health officer at the health center)*

Mothers illustrate how local cultural traditions and informal community networks actively reinforce the use of alternative remedies over formal clinical evaluations.

*“In our culture, it is believed that children stand after a prolonged period of staying on their own, with thin children standing earlier. This is seen as a cultural norm where family conditions are thought to affect a child’s development. For example, if the parents are quick, the child will be quick as well; if not, the child may lag behind. This is considered a family–related condition.” (Mother of a developmentally delayed child)*

Conversely, most healthcare providers observed that the community is generally cooperative in engaging with established child health services. They noted that community members are typically willing to accept and actively participate in the delivery of these services.

“*To provide a continuous monitoring for example if we take children who come to receive EPI services, they come to receive the immunization service whether or not they are sick. So, we can proceed with the follow–up even after the children finish the immunization schedule... Our community could not challenge us to receive whatever service that we told them would benefit their child.‘*” (Key informant, public health officer)

### Theme 6: Parental related factors

This theme maps to the outer setting domain (patient needs and resource) of the CFIR and is divided into two socio-demographic sub-themes.

#### Poor parental knowledge.

Participants described how a parental lack of health literacy regarding the specific milestones of normal child development serves as a primary barrier to early entry into care. Parents frequently lack the foundational awareness required to conceptualize a developmental delay as a treatable medical condition. When developmental milestones are visibly missed, the lack of clear, actionable medical information causes intense parental stress and anxiety. Compounded by this anxiety, parents develop a dilemma over whether their concerns will be validated, accepted or even understood by primary health center staff. Many parents choose to stay home, consequently allowing the child’s underlying condition to worsen before seeking professional consultation.

“*They connect this thing with spiritual problems. When they come to us first, they think the problem is very complex and relate it to so many things. They had a feeling of regret, like they were the ones to put their child into this. We tell them the scientific reason for the occurrence of the problem, and teach them what is expected from them to do*. ” (GP working at health center)

“*Some people asked about what happened to the child. What can I say? It is not an infection, like the common cold. what can I say? How can I say the child is unable to crawl, unable to walk … or else how could I explain this? …Wondering in this dilemma, I stayed home.*” (Mother of a developmentally delayed child)

#### Parental economic status.

Almost all parents mentioned that household financial difficulties represent a severe structural barrier within the outer setting that directly impacts parental psychology. The high out–of–pocket expenses required to pursue advanced diagnostics, cover long–distance travel to urban referral centers, and maintain long–term intervention programs induce intense emotional distress, guilt, and profound isolation among low–income families. Faced with a structural deficit in localized, affordable support services, these acute financial constraints compromise a family’s ability to maintain clinical follow–up, forcing parents to abandon the assessment pathway entirely to remain in the household.

*“I know my child has a developmental delay, and I experience a range of emotions including sadness, confusion, fear, guilt, and frustration. I feel overwhelmed by the challenges ahead and worried about how I will support my child’s needs, especially given my financial constraints. Additionally, I feel isolated and alone in managing my child’s developmental delay due to the lack of resources and support services.”* (Mother of a developmentally delayed child)

#### Integration of findings with the Consolidated Framework of Interventional Research (CFIR).

Although the thematic analysis was conducted inductively, the resulting themes were subsequently mapped to the CFIR to systematically categorize the barriers and facilitators across major implementation domains (Table 2).

**Table 2:** Mapping of the barriers and facilitators across major implementation domains of the Consolidated Framework for Implementation Research (CFIR).

CFIR Domain	Corresponding study theme	Key constructs identified	Implementation role
Outer setting	Theme 1: Policy government, and stakeholder commitment	• External policies & Incentives • Cosmopolitanism	Facilitators: Integration of screening mandates into revised national IMNCI guidelines; NGO partnerships. Barrier: Low government prioritization; lack of multisectoral collaboration.
	Theme 5&6: community norms & parental factors	• Patient needs & resources	Barrier: Cultural stigmas, spiritual attributions of delays, severe parental financial constraints, and low health literacy
Inner setting	Theme 2: Healthcare system and infrastructure	• Available resources • Access to knowledge & information • Structural characteristics • Networks & Communications	Barrier: Cramped workspaces, tool shortages, extreme provider workloads, training gaps. Facilitator: An established, highly functional three–tier referral system.
Characteristics of individuals	Theme 3: Human resources	• Knowledge & Beliefs about the Intervention • Self–efficacy • Motivation	Barrier: Widespread clinical skills gaps and low provider confidence in diagnosing delays. Barrier: Low provider motivation driven by the perception that developmental assessments are too time–consuming for high–volume settings.
Intervention Characteristics	Theme 4: Clinical and assessment–related factors.	• Complexity • Cost	Barrier: The multi–causal, non–acute nature of developmental delays requires specialized, multi–disciplinary diagnostic pathways that are structurally and financially expensive for both facilities and families.
Process	Cross–cutting domain	• Planning	Facilitator: Leveraging established child–health platforms (EPI and nutrition programs) as pre–existing operational structures to expand developmental screening coverage efficiently without building new systems from scratch

## Discussion

The study identified six key themes driving implementation and access challenges, highlighting that current developmental assessment services are inadequate, narrowly focused, and lack accessibility. These themes include: (1) the limited government commitment, attention, and stakeholder funding compared to other child health programs; (2) insufficient healthcare system capacity and infrastructure, including a lack of dedicated space and screening tools; (3) human resource constraints, characterized by provider knowledge and skills gaps, low clinical confidence, and high workloads; (4) clinical and assessment–related factors, specifically the high complexity and resource–intensive nature of the diagnostic pathway; (5) community norms, traditional beliefs, and stigmas that hinder timely clinical engagement; and (6) parental–related factors, including a lack of knowledge and financial constraints that restrict service utilization.

Our study indicates that healthcare providers primarily focus on developmental assessments only when parents express concerns or when children present with illness or other clinical manifestations. While delayed detection was also reported in Saudi Arabia [18], the United States (US) [29], and Singapore [30], settings where children typically received developmental monitoring in the “well–child” clinics, the underlying reasons in Ethiopia are uniquely characterized by “a ‘survival-oriented’ clinical priority. “Unlike high–income settings, where missed assessments are often attributed to insurance reimbursement protocols or preventive care schedule limits [29,30], Ethiopian providers prioritize the urgent demands of acute illness and malnutrition [16]. This focus on immediate survival, while necessary, is insufficient given that a high proportion of children have developmental problems. This suggests a critical need to transition from a reactive, survival–only model to a comprehensive strategy that integrates developmental surveillance into routine pediatric care.

Our study found that the current primary healthcare (PHC) structure, which is central to the Ethiopian healthcare system [31,32], has the potential to facilitate developmental assessments by sharing responsibilities across different levels, thereby easing the individual provider workload and increasing service availability. However, at the PHC level, developmental assessment lacks the institutional commitment, structural support, stakeholder engagement, and funding standard in immunization and other child health programs. This gap reflects global findings regarding the disparity between general health program awareness and early intervention for developmental delays [33]. In LMICs, pediatric programs predominantly target child survival [6,14,34,35]. In Ethiopia, where data suggests up to 66% of children under five show developmental delays [36], the insufficiency of an exclusively survival–focused approach is clear, highlighting the need for comprehensive developmental strategies.

Beyond clinical priorities, the lack of physical infrastructure within the PHC structure remains a significant barrier. Participants identified limited workspace, insufficient equipment, and the lack of manuals as significant barriers to providing developmental assessments. These findings are consistent with research from the United States, where inefficient systems of care were identified as major barriers [10,37]. They also mirror local evidence from Addis Ababa, where only a small percentage of health professionals used standardized developmental screening tools or the IMNCI context classification system for child developmental delay [16]. However, in the Ethiopian context, these barriers are exacerbated by a disparity in funding and stakeholder engagement compared to high–priority programs like immunization. Although routine EPI offers ideal contact points to reach healthy children, space and capacity bottlenecks prevent facilities from co-locating developmental assessments. Consequently, the current PHC structure lacks the necessary infrastructure to implement standardized and culturally relevant assessment tools, leaving providers dependent on inconsistent, informal assessment methods despite global evidence recommending their use [24,38,39].

Our study also identified insufficient training, high turnover of trained staff, and limited awareness of child development and early identification mechanisms of delays as barriers. This is consistent with studies from the United States [25] and Saudi Arabia [18], which showed that providers lack consistent training, educational materials or updated clinical information. Consequently, providers in this study expressed a lack of confidence in making developmental decisions and cited poor personal knowledge of systematic developmental assessments. Similarly, research conducted in Singapore [40] and Ethiopia [16] found that two–thirds of providers lacked the necessary in–service education and 60.6% of health professionals had poor knowledge of child developmental milestone assessment. A lack of developmental awareness among practitioners can lead to overlooking subtle delays or specific deficits during treatments targeted at unrelated acute illnesses. Variations in provider knowledge may result in highly inconsistent assessment and screening practices during routine pediatric visits [41].

In this study parental lack of knowledge, awareness, and financial resources emerged as a major barrier preventing access to services and treatment for developmental issues. Studies in Pakistan and Saudi Arabia found significant gaps in mothers’ knowledge of developmental red flags (42.–59% and 37.6%, respectively) [42,43]. Additionally, low parental economic status often prevents families from accessing diagnostic services or pursuing further evaluation when a referral is given. Research from Australia and Romania similarly identified financial obstacles as a major hindrance to accessing specialized healthcare [44,45]. These financial delays frequently result in late clinical presentations of manageable health conditions. Implementing programs that subsidize healthcare costs for low–income families and developing community–based health programs that offer free screenings and targeted health education should be considered.

Our study highlighted that stigma is deeply prevalent within the sociocultural context, with both providers and parents noting that community beliefs about developmental delays are significant barriers to early intervention. Such beliefs often associate these conditions with spiritual or societal curses, leading to recommendations for spiritual or traditional interventions instead of formal clinical services. The study found that parents of children with developmental issues often face stigma, reflecting findings from a similar study in Butajira, Ethiopia, where families of children with autism faced systemic exclusion and negative social judgments [24]. To avoid social exclusion and judgment, some families keep children with developmental issues hidden at home, seeking formal healthcare only as a last resort when the child develops severe, unrelated medical illnesses. Fear of stigma thus emerged as a key factor contributing to delayed healthcare utilization.

Furthermore, stigma places a significant psychosocial burden on parents [46], contributing to stress, social isolation, and emotional exhaustion, which can reduce their capacity to engage in warm, responsive interactions with their children. These pressures limit opportunities for play, communication, and developmental stimulation, meaning stigma not only affects parents individually but also disrupts the daily relational patterns that shape parent–child interactions [47,48]. Because these interactions are reciprocal, a child’s behavioral or emotional difficulties can further increase parental stress, creating a feedback cycle that adversely affects family dynamics and overall psychosocial well–being [49]. Addressing community stigma is therefore essential for improving care–seeking behaviors, strengthening parent–child relationships, and supporting healthier family functioning in the context of developmental disorders [50].

We selected the implementation framework (CFIR) because it provides a structured approach to understanding what works, where, and why across multiple contexts, allowing researchers to focus on the constructs most relevant to their study environment [28]. Mapping our inductively derived themes to the CFIR helped organize barriers and facilitators at multiple interacting levels: the Outer setting (community norms, parental knowledge, and socioeconomic factors), the Inner setting (resources, referral systems, and communication networks), the Characteristics of Individuals (provider knowledge, motivation, and self–efficacy), and Intervention characteristics (complexity and cost). While no single theme fully aligned with the Process domain on its own, overlapping information highlighted the immense value of integrating developmental assessments into existing child health platforms such as EPI and nutrition programs. Overall, the CFIR provided a clear analytical lens to interpret findings, identify multi–level determinants, and guide future implementation strategies for developmental assessment [28,51].

### Strengths and limitations of the study

A primary strength of this study is the direct involvement of both healthcare professionals with experience in developmental assessment and parents of children with developmental delays, allowing for a comprehensive analytical identification of implementation and access barriers and facilitators. Including parents from a rural town, most of whom had children living with confirmed developmental delays, also added lived–experience insights.

However, a limitation is that the study was conducted in one zone with purposefully selected participants. While this purposive approach means the findings are not intended for statistical generalization, their transferability to other regions in Ethiopia remains high. This is because the fundamental structure of the primary healthcare system, the staffing patterns, and the reliance on national child health guidelines (like IMNCI) are standardized across the country. Therefore, the core institutional barriers and programmatic constraints identified here likely reflect the realities of similar districts. Nevertheless, transferring these findings to other regions must be approached with caution due to varying health system performance profiles, such as differences in intersectoral collaboration quality, equipment availability, and localized financial resources.

Finally, the scarcity of peer–reviewed qualitative literature on developmental assessment barriers within LMICs, and specifically within the Ethiopian primary healthcare context, presented a significant challenge for the discussion. Because there were very few local studies available, it was difficult to contextually compare and contrast our qualitative themes within a similar socioeconomic framework. Consequently, several findings had to be discussed in relation to evidence from high–income settings. While these comparisons provide a valuable global benchmark for provider barriers, the vast structural and socioeconomic differences between these settings must be kept in mind, as they may limit how directly those comparisons apply to our local context.

### Implications of the study

The findings of this study have several important implications for public health policy, research and clinical practice. Improving developmental assessments requires a coordinated consideration of the existing healthcare system and the active involvement of multi–sectoral stakeholders.

**Policy and Resource Allocation:** The limited recognition of developmental delays as a public health priority contributes to weak policy support and inadequate resource allocation. In practice, this means that ministries of health and local health authorities should integrate formal developmental screening mandates into national child health policies, and allocate dedicated budgets for early identification and intervention materials. Strengthening national surveillance through robust prevalence studies, community–based assessments, and routine health data registry tracking would generate the localized epidemiological evidence needed to guide resource planning and targeted interventions.

**Service Delivery:** Locating developmental assessments within overcrowded, high–volume sick–child clinics undermines service quality and worsens provider burnout. To reinforce existing health–system structures without creating separate, standalone programs that deplete limited workforce resources, a practical step is for health systems to establish designated “developmental monitoring corners” within existing child health spaces, specifically linked with routine immunization, nutrition, and growth monitoring services. Because existing clinic rooms are frequently highly condensed and cramped, these corners must be designed to maximize limited physical footprints through low–cost spatial reconfigurations, such as using simple privacy curtains or re–arranged furniture to zone off a dedicated assessment area.

These corners should be equipped with culturally and linguistically appropriate screening tools and simple job aids [52,53]. Strengthening provider capacity through regular, practical in–service training, structured mentorship, and supportive supervision is essential to ensure accurate assessments and timely referrals.

**Community and Parental Awareness:** Parents’ experiences of limited knowledge and stigma highlight the need for practical, community–level engagement. Awareness campaigns, delivered through HEWs, schools, religious institutions, and local media, can promote accurate information about developmental milestones and underscore the importance of early intervention. Facility–based health education sessions and parent–support groups can provide concrete guidance on recognizing developmental concerns, seeking timely care, and fostering responsive parent–child interactions. Such efforts can reduce stigma, encourage active help–seeking behaviors, and create a more supportive social environment for vulnerable families.

## Conclusion

This study highlights that the successful implementation of child developmental assessment within routine pediatric services in Ethiopia relies on a complex interplay of institutional problems and pressing sociocultural realities. The findings reveal that current developmental assessments are highly constrained by system–level barriers, including a lack of standardized screening tools, outdated or unavailable national guidelines, a severe workforce training gap, and infrastructural problems, such as a lack of private, dedicated OPD spaces. These institutional barriers are further compounded by social realities that shape families’ access to care, including community–based stigma, financial constraints among low–income households, and a frequent preference for traditional or spiritual healing. Concurrently, community health extension systems, stakeholder collaboration, and existing child health touchpoints emerge as important structural facilitators that can be leveraged to optimize care.

To navigate these challenges, a critical operational tension must be resolved: while providers creatively attempt to use routine well–baby and sick–baby clinics, they emphasize that severe frontline workloads and the lack of dedicated clinic space make performing these detailed assessments highly difficult. A practical, implementation–focused approach, one that strengthens frontline capacity, reinforces existing health–system structures, and builds on community trust, offers a good path forward. Ultimately, balancing frontline willingness against these intense resource limitations with a coordinated policy commitment will ensure developmental assessment shifts from a neglected, coincidental service into a reliable and sustainable part of routine pediatric care.

## Supporting information

S1 File Interview guide. Semi-structured interview guides used for key informant interviews with healthcare professionals and parents.
(ZIP)

S2 File Supporting data. Transcript and data supporting the study findings.
(ZIP)

## Abbreviations

DD: Developmental delay
GB: Global Burden of Disease
LMICs: Low– and Middle–Income Countries
SDGs: Sustainable Development Goals
U5MR: Under–5 mortality Rate
WHO: World Health Organization
