# Supplementary material for: Why are the implementation of and access to child developmental assessment within routine healthcare so difficult in Ethiopia? A descriptive qualitative study
Source: PLoS One. 2026 Aug 10;21(8):e0355686. doi: 10.1371/journal.pone.0355686 (PMC13456246; doi:10.1371/journal.pone.0355686)
Supplement: Supplementary file 1 [file pone.0355686.s001.zip › Interview guides/Interview gide for higher level stakeholders.docx]

**Study Title-** Assessment of Barriers and facilitators to provide and access developmental assessment service by providers and parents, respectively.

**Participants:** Zonal/Woreda Maternal and Child health focal person.

**IRB No: Session ID:** [________]	**Date (DD/MM/YY):** ____ /____ / ____

Name of Interviewer: ___________________________________ Signature _________________

| **Before the Interview check the following** | | | | | |  |
| --- | --- | --- | --- | --- | --- | --- |
| 1. ***Introduce yourself and the study*** |  | | |  |  |  |
| 2. ***Make sure the participant is eligible*** |  | | |  |  |  |
| a. What is the Participant's profession? | - pediatric nurse/ - Nurse | | | - Health Officer |  |  |
| ***Obtain informed consent [READ CONSENT SCRIPT]*** | | | | | |  |
| a. Did you answer any questions he/she had? | - YES | | | - NO |  she/He didn't ask/ |  |
| b. Did he/she consent to participate? | - YES | | | - NO |  |  |
| c. *Optional:* Did He/ SHE ACCEPT a copy of the consent form? | - YES | | | - NO |  |  |
| 3. ***Obtain permission to audio record*** |  | | |  |  |  |
| Did He/she give you permission to be recorded? |  | | |  |  |  |
| a. YES */* | *TURN ON RECORDER /* | | | | |  |
| b. NO/ | *THANK HER/him AND CONCLUDE SESSION* | | | | |  |
| 4. ***Turn on the tape recorder(s) and say:*** |  |  |  | | | |
| ***“Have you consented to this interview? (record her answer)*** *Thank you for giving me permission to record, today is XXX ( date) this is woreda XXX interviewer XXX interview code XXX*” | | | | | |  |

**Basic information of the participant**

**Profession: Experience:**

**Age: Sex:**

| **Part 1: Introduction**  The purpose of this section is to understand what healthy development is and how you do developmental assessment  Thank you again for your willingness to participate in this study and to help our research team learn more about your experience in developmental assessment and the service. Feel free to answer as you remember and understand. There are no wrong or right answers.  Shall we continue? | |
| --- | --- |
| 1. | Tell me what is currently being done to address those issues related with developmental problem in the healthcare system? |
| 2. | What programs or services are available or organizations that are working on child developmental assessment? |
| 3. | What are the facilitators to provide developmental assessment in the primary health care? |
| 4. | Regarding child developmental assessment, what are greatest strengths or assets around implementing the service? |
| 5. | What do you think leaders and decision‐makers in the healthcare system can do to help improve child developmental assessment? |
| **Part2** | Barriers to development assessment |
| 6. | please tell me what you see as the issues or concerns related to child development assessment services for the communities or populations with whom you work? |
| 7. | What do you see as the barriers around improving child development assessment in Gamo Zone? |
| 8. | What are the consequences to the health care system in not addressing these issues? |
| 9. | What is the impact of the above consequences on the community/population for whom you work or on your organization/agency? |
