# Supplementary material for: Why are the implementation of and access to child developmental assessment within routine healthcare so difficult in Ethiopia? A descriptive qualitative study
Source: PLoS One. 2026 Aug 10;21(8):e0355686. doi: 10.1371/journal.pone.0355686 (PMC13456246; doi:10.1371/journal.pone.0355686)
Supplement: Supplementary file 1 [file pone.0355686.s001.zip › Interview guides/Interview guid for parental assessment.docx]

**Study Title –** assessment of Barriers and facilitators to provide and access developmental assessment service by providers and parents, respectively.

**In-Depth interview guide for parents**

IRB No:

Session ID: [________]	Date (DD/MM/YY): ____ /____ / ____

Name of Interviewer: ___________________________________________________________

| **Before the Interview check the following** | | | | | |
| --- | --- | --- | --- | --- | --- |
| 1. ***Introduce yourself and the study*** |  | |  | |  |
| 2. ***Make sure the participant is eligible*** |  | |  | |  |
| a. Is the participant a mother? |  YES | |  NO | |  |
| b. Does the child below five years |  YES | |  NO | |  |
| c. Was the child has developmental problem |  YES | |  NO | |  |
| ***Obtain informed consent [READ CONSENT SCRIPT] /*** | | | | | |
| a. Did you answer any questions she had? |  YES | |  NO | |  she didn't ask/ |
| b. Did she consent to participate? |  YES | |  NO | |  |
| c. *Optional:* Did she accept a copy of the consent form? |  YES | |  NO | |  |
| 3. ***Obtain permission to audio record*** |  | |  | |  |
| Did she give you permission to be recorded? |  | |  | |  |
| a. YES | *TURN ON RECORDER* | | | | |
| b. NO | *THANK HER AND CONCLUDE SESSIOn* | | | | |
| 4. ***Turn on the tape recorder(s) and say:*** |  |  | |  | |
| ***“Have you consented to this interview? (record her answer)*** *Thank you for giving me permission to record, today is XXX (date) this is woreda XXX interviewer XXX interview code XXX*” | | | | | |

**Basic information of the participant**

**Age of the mother/care giver: number of children in the household**

**Age of the child: Sex:**

| **Part 1: Concepts of healthy development and developmental assessment**  **(In this section I am going to ask you what you think about healthy development and developmental assessment in general. Tell me what you think about the issues that will be raised)**  The purpose of this section is to understand what healthy development is and how you do developmental assessment  Thank you again for your willingness to participate in this study and to help our research team learn more about your experience in developmental assessment and the service. You are selected in this study as you we feel you are related with the child’ development as you are a mother. Feel free to answer as you remember and understand. There are no wrong or right answers.  Shall we continue? | |
| --- | --- |
| 1. | Tell me a little about yourself and your family.   - How old is this child? Where did you give birth to this child? - How many children do you have? - Where do you receive child health service for this child? - Does this child vaccinated? - Can you please tell me about your general experience when attending the health facility with your child? |
| 2. | When do you say a child has a healthy development? Why? |
| 3. | When do you say a child has unhealthy development? Why?   - What are the signs of unhealthy development? |
| 4. | What are the most developmental related health problems a child encounter? |
| 5. | How do you know if someone has developmental problem in your area (in this setting? location of the health center)?   - At what stage of the problem do you usually know the child has developmental problem? / - When do you often recognize a child has a developmental problem? |
| **Part2** | **Developmental assessment** |
| 6. | Have you ever heard about developmental assessment?  If yes   - What is developmental assessment? - Where is the service provided? By whom?   If no   - what do you think it is? |
| 7. | Has (child name) had a developmental check before?   - If so, who did it and why? - If not, why? What is the reason they had for not receiving the developmental assessment? Why? |
| 8. | Before going to the health care facility, did you have any concerns about (child name)’s development? |
| 9. | Can you please tell me about your experience of going through the developmental check with health care provider?   - What do you like most? Why? - What don't you like? Why? - What is the question you ask? - What is your concern about developmental assessment? |
| **Part 3** | **Barriers to access developmental assessment** |
| 10. | - What is the factor s at a health facility that could be barrier for you to access developmental assessment for you? - What do you think are the factor at providers side that could be barrier to access developmental assessment for you? - What other factors be barrier to access developmental assessment for you? |
| **Part 4** | **Facilitators to access developmental assessment**   - What is the factor s at a health facility that could be facilitators to access developmental assessment? - What do you think are the factor at providers side that could be facilitators to access developmental assessment? - What other factors be facilitators to access developmental assessment? |
| **Part 5** | **For parents having developmental delay child ask the following questions?** |
| 11. | When did you first notice your child has developmental difference or delay? |
| 12. | How did you feel about the issue? |
| 13. | What made you become concerned about the developmental delay? |
| 14. | Where did you seek help? Why you did seek help? |
| 15. | Were there any challenge in seeking help? What happened next? |
| 16. | How confident did you feel about overcoming obstacles to getting the help for your child? |
| 17. | Did anything prompt you to seek help?(event, media, professionals, other people)? |
| 18. | Did any other personal factor impact your experience?(culture,income,location) |
