# Supplementary material for: Why are the implementation of and access to child developmental assessment within routine healthcare so difficult in Ethiopia? A descriptive qualitative study
Source: PLoS One. 2026 Aug 10;21(8):e0355686. doi: 10.1371/journal.pone.0355686 (PMC13456246; doi:10.1371/journal.pone.0355686)
Supplement: Supplementary file 1 [file pone.0355686.s001.zip › Interview guides/Interview guide for Health profestional .docx]

**Study Title-** Assessment of Barriers and facilitators to provide and access developmental assessment service by providers and parents, respectively.

**Participants:** Health professionals working in under five clinic /pediatrician.

**IRB No: Session ID:** [________]	**Date (DD/MM/YY):** ____ /____ / ____

Name of Interviewer :___________________________________signature _________________

| **Before the Interview check the following** | | | | | | |
| --- | --- | --- | --- | --- | --- | --- |
| 1. ***Introduce yourself and the study*** |  | | |  |  | |
| 2. ***Make sure the participant is eligible*** |  | | |  |  | |
| a. What is the Participant's profession? | - pediatric nurse - Nurse | | | - Pediatrician - Health Officer |  | |
| ***Obtain informed consent [READ CONSENT SCRIPT]*** | | | | | | |
| a. Did you answer any questions he/she had? | - YES | | | - NO | she/he didn't ask/ | |
| b. Did she consent to participate? | - YES | | | - NO |  | |
| c. *Optional:* Did He/she accept a copy of the consent form? | - YES | | | - NO |  | |
| 3. ***Obtain permission to audio record*** |  | | |  |  | |
| Did He/she give you permission to be recorded? |  | | |  |  | |
| a. YES */* | *TURN ON RECORDER /* | | | | | |
| b. NO/ | *THANK HER AND CONCLUDE SESSION* | | | | | |
| 4. ***Turn on the tape recorder(s) and say:*** |  |  |  | | |  |
| ***“Have you consented to this interview? ( record her answer)*** *Thank you for giving me permission to record, today is XXX ( date) this is woreda XXX interviewer XXX interview code XXX*” | | | | | | |

**Basic information of the participant**

**Profession: Experience:**

**Age: Sex:**

| **Part 1: Concepts of healthy development and developmental assessment**  **(In this section I am going to ask you what you think about healthy development and developmental assessment in general. Tell me what you think about the issues that will be raised)**  The purpose of this section is to understand what healthy development is and how you do developmental assessment  Thank you again for your willingness to participate in this study and to help our research team learn more about your experience in developmental assessment and the service. You are selected in this study as you provide service in the area. Feel free to answer as you remember and understand. There are no wrong or right answers.  Shall we continue? | |
| --- | --- |
| 1. | Tell me your daily routine work in under five OPD |
| 2. | When do you say a child has a healthy development? Why? |
| 3. | When do you say a child has unhealthy development? Why?   - What are the signs of unhealthy development? |
| 4. | What are the most developmental related health problems a child encounter? |
| 5. | How do you know if someone has developmental problem in your area (in this setting? location of the health center)?   - At what stage of the problem do you usually know the child has developmental problem? / When do you often recognize when a child has a developmental problem? |
| **Part2** | **Developmental assessment** |
| 6. | What services do you do to children to know their developmental status, currently?   - How do you provide developmental assessment either in the community or health facility? - What materials you use to perform developmental assessment? - In your observation do you think the service you are providing timely, adequate? Why? HOW? - What makes the service you are offering different from others? |
| 7. | Do you do routine developmental assessment for all children?  **if yes**   - What proportion of children do you think will come to a health facility receive developmental assessment?   **If no** on what grounds/for what reason they decide to evaluate one child but not the other |
| 8. | What do you do when you know when a child has developmental problem?   - How do you follow the child? |
| **Part 3** | **Barriers for developmental assessment** |
| 9. | Think about a child who received developmental assessment service, What does your interaction look like;   - Where do you do the assessment? Why? - How long it takes? Why? - What material you use? Why? - What is the most common problem you encounter to do developmental assessment? - What is your concern while doing the assessment? |
| 10. | Think about a child who you want to do developmental assessment for and failed to provide?   - What is the reason you have for not doing the developmental assessment? Why? - What is the most reason you have for not doing the developmental assessment? Why? |
| 11. | Think about a child who you provide but the parents refuse to received developmental assessment service?   - What is the reason they had for not receiving the developmental assessment? Why? - What is the most common reason they had for not receiving the developmental assessment? Why? |
| 12. | From your observation:   - What is the factor s at a health facility that could be barrier to do developmental assessment? - What is the most important factor at a health facility that could be barrier to do developmental assessment? - What do you think are the factor at providers side that could be barrier to do developmental assessment? - What do you think is the most important factor at providers side that could be barrier to do developmental assessment? - What other factors be barrier to do developmental assessment? |
| **Part4** | **Facilitators for developmental assessment** |
| 13. | From your observation:   - What are the factor at a health facility that encourage to do developmental assessment? - What are the most important one the factor at a health facility that encourage to do developmental assessment? - What do you think is the most important factor at providers side that encourage to do developmental assessment? - What are the most important one the factor at providers side that encourage to do developmental assessment? - What other factors be facilitators to do developmental assessment? |
| **Part 5** | **Perception towards continuous developmental monitoring?** |
| 14. | What do you think of changing the current mode of developmental assessment to developmental monitoring by using screening tool?   - What are the possible benefits ? - What would be the possible challenges? - What are the possible facilitators? |
| **Part 6** | **Parental access to developmental assessment** |
| 15. | In your observation what do parents do when they know the child has developmental problem?   - Where do they go to get health services? - At what stage do they seek health care? - What circumstances does lead them to seek health services? |
| 16. | In your opinion what is the attitude of parents towards developmental assessment? Why?   - What is the characteristics of parents that mostly visit the clinic? - What is the characteristics of parents that come to follow-up visits? - What do they like most? Why? - What don't they like? Why? - What is the question they ask? - What is their concern about developmental assessment? - What is the reason they give not to receive developmental assessment? |
| 17. | Who is the most important family or community member in seeking developmental services? how? |
