# Supplementary material for: Why are the implementation of and access to child developmental assessment within routine healthcare so difficult in Ethiopia? A descriptive qualitative study
Source: PLoS One. 2026 Aug 10;21(8):e0355686. doi: 10.1371/journal.pone.0355686 (PMC13456246; doi:10.1371/journal.pone.0355686)
Supplement: Supplementary file 1 [file pone.0355686.s001.zip › Interview guides/Interview guide for middle level stakeholders.docx]

**Study Title-** Assessment of Barriers and facilitators to provide and access developmental assessment service by providers and parents, respectively.

**Participants:** MCH focal person.

**IRB No: Session ID:** [________]	**Date (DD/MM/YY):** ____ /____ / ____

Name of Interviewer: ___________________________________ Signature _________________

| **Before the Interview check the following** | | | | | |  |
| --- | --- | --- | --- | --- | --- | --- |
| 1. ***Introduce yourself and the study*** |  | | |  |  |  |
| 2. ***Make sure the participant is eligible*** |  | | |  |  |  |
| a. What is the Participant's profession? | - pediatric nurse/ - Nurse | | | - Pediatrician - Health Officer |  |  |
| ***Obtain informed consent [READ CONSENT SCRIPT]*** | | | | | |  |
| a. Did you answer any questions he/she had? |  YES | | |  NO |  she didn't ask/ |  |
| b. Did he/she consent to participate? |  YES | | |  NO |  |  |
| c. *Optional:* Did He/SHE ACCEPT a copy of the consent form? |  YES | | |  NO |  |  |
| 3. ***Obtain permission to audio record*** |  | | |  |  |  |
| Did He/she give you permission to be recorded? |  | | |  |  |  |
| a.  YES */* | *TURN ON RECORDER /* | | | | |  |
| b.  NO/ | *THANK HER/him AND CONCLUDE SESSION* | | | | |  |
| 4. ***Turn on the tape recorder(s) and say:*** |  |  |  | | | |
| ***“Have you consented to this interview? ( record her answer)*** *Thank you for giving me permission to record, today is XXX ( date) this is woreda XXX interviewer XXX interview code XXX*” | | | | | |  |

**Basic information of the participant**

**Profession: Experience:**

**Age: Sex:**

| **Part 1: Introduction**  Thank you again for your willingness to participate in this study and to help our research team learn more about your experience in developmental assessment and the service. You are selected in this study as you provide service in the area. Feel free to answer as you remember and understand. There are no wrong or right answers.  Shall we continue? | |
| --- | --- |
| 1. | Tell me your daily routine work in your coordination unit regarding child growth and development? |
| 2. | When do you say a child has a healthy development? Why? |
| 3. | When do you say a child has unhealthy development? Why?   - What are the signs of unhealthy development? |
| 4. | What are the most developmental related health problems a child encounter? |
| 5. | How do you know if someone has developmental problem in your area?   - At what stage of the problem do you usually know the child has developmental problem? |
| **Part2** | **Developmental assessment** |
| 6. | What are the programs that your organization implementing to identify children with developmental problem, currently?   - How do you provide developmental assessment either in the community or health facility? - How do you explain the available infrastructure/logistics to provide the service? - What are capacity building activities done so far regarding developmental assessment? - How do you explain the skill and knowledge of providers to do developmental assessment - In your observation do you think the service you are providing timely, adequate? Why? HOW? |
| 7. | What proportion of children do you think will come to a health facility receive developmental assessment? |
| **Part3** | **Facilitators for developmental assessment** |
|  | - What are the facilitators to do developmental assessment? |
| **Part 4** | **Barriers for developmental assessment** |
| 8. | From your observation:   - What are the barrier to do developmental assessment? |
| **Part 5** | **Perception towards continuous developmental monitoring?** |
| 9. | What do you think of changing the current mode of developmental assessment to developmental monitoring by using screening tool?   - What are the possible benefits? - What would be the possible challenges? - What are the possible facilitators? |
| **Part 6** | **Parental access to developmental assessment** |
| 10. | In your observation what do parents do when they know the child has developmental problem?   - Where do they go to get health services? - At what stage do they seek health care? - What circumstances does lead them to seek health services? |
| 11. | What do you see as the barriers for parents to access child development assessment?   - What are the differences in access to child development assessment (monitoring)? - Who is responsible for resolving barriers? why? |
| 12. | Who is the most important family or community member in seeking developmental services? how? |
