# Supplementary material for: Why are the implementation of and access to child developmental assessment within routine healthcare so difficult in Ethiopia? A descriptive qualitative study
Source: PLoS One. 2026 Aug 10;21(8):e0355686. doi: 10.1371/journal.pone.0355686 (PMC13456246; doi:10.1371/journal.pone.0355686)
Supplement: Supplementary file 2 [file pone.0355686.s002.zip › data/Transcripit 1.docx]

**Transcript 1**

1. What programs or services are available around child development monitoring in Ethiopia as well as Gamo zone?
   - What are the directions set around progress monitoring

**Answer:** The activities to prevent developmental delay starts first from pregnancy period. During the pregnancy period there are so many activities that can be done on the mother, especially on the first 1000 days which is from pregnancy till the child become 2 years old. Pregnant mothers during there pregnancy period engage in activities in the first one thousand days that can prevent stunting of the child after giving birth and this has been implemented so far in Ethiopia. Ethiopia is currently working not only on children, rather we are following a life cycle approach. The intervention in this life cycle approach starts after the child is born then to adolescent and continued to adulthood to tackle developmental problems. The thing that you have mentioned (developmental assessment) is one of the interventions.

Beginning from pregnancy then till the child is born infant and young child feeding related works has been implemented. Therefore, mothers told to feed the child for the first six months exclusive breast feeding then to start complementary feeding. In addition to this nutritional intervention has been implement by health extension also in the community and in the health facilities. Regarding Child feeding in every kebele food preparation lesson is given. Even if they have their own limitations this is what is being exercised here.

Regarding child developmental monitoring every month there is growth monitoring. what is done on this is to encourage mothers whose child is growing well and counsel those mothers whose child is lagging behind his/her age so that he can improve the growth in the next time.

**2. Please explain the current policies related to child development monitoring at the program level**

**Answer:** Good, it has been a year and some months since early childhood development is being implemented. The training is even given integrated with IRT training for health extension at the time. however, in principle the health extension workers currently use ICMNCI which previously is called ICCM to screen and classify the child. For children in 2 to 5 years, they classify as confirmed developmental delay, no developmental delay. Also, for those children less than 2 years they will be categorized as suspected developmental delay and no developmental delay. The updated ICMNCI training should be given for the health extension workers. But this one has not got as much focus as the IRT training in which ICMNCI training was given for the health professionals. Chart booklets are prepared in Amharic for health extension workers but regarding this problem there is no chart booklet prepared in Amharic and also detail training is not given to them. A simple orientation was given for providers to assess all ill under five children who visit the health facility for their development. The logic behind the assessment of the children is to classify and take appropriate actions or measures. However, the training was not as such detail so they do the assessment and report for us. We are getting reports. But is this right? we did not do further confirmatory works. This is because they did not take training and they got orientation on IRT and the chart booklet has no much information about childhood development and the training is not sufficient. At the time they had training on why we invest in childhood development and what child developmental assessment is but they do not get knowledge on how to assess, classify and then treat. Thus, currently it is the focus of government. However, its not implement on ground as other programs like they treat diarrhea, pneumonia. Also, they do not have skills to assess, classify and treat. The training was given by regional health bureau for a limited number of health centers which has highest flow of children visit. One individual from zonal health office gets the training. This is what exists still now and its not implement in a good way on ground.

Regarding child health, health extension workers are the one who highly involve in child related issues. Thus, the training should be given in detail for them but still now much work is not done.

Still now the chart booklet is not translated from English to Amharic in order to be easy to use for them. Currently, they are exercising the assessment by simple orientation. During the training they do not exercises detail assessment, classification, treatment and actions to be taken for children who had confirmed developmental delay.

2. You told me that the health professionals did not get enough training and the program is implementation involves from zonal health beaure to health extenstion workers. Would you please tell me how the service is being delivered in the health care system?

**Answer:** The service is now delivered integrated with nutrition related activities. There are activities related to child safety during pregnancy and after the child is born till 2 years old or the first one thousand days. In the community health extension workers are nearest and they identify developmental delay or other childhood development related problems for those children who are ill and come to visit the health facility not as a routine screening. The mother might bring the child and when one child comes to the health facility may be ill of one or two diseases then the health professionals assess all things and tell the mother. Whether or not the mother ask the presence of a problem, the health professional assess, classify and treat or refer if the problem is beyond there capacity to treat. Generally, this is what being done in the community.

When we go up from the community these activities should be done well in the health center and also hospitals. Children come passing the service in the community when they are ill and there should be assessment. All children should be assessed, classify and action should be taken but it’s seen that there is lack of attention by health professional to do the work. Health extensions workers are the one nearest to the children and even if they come passing the health extension workers to other health center they should be examined. The children should pass ICMNCI service to get the ICMNCI service at health center then ICMNCI service from primary hospital and general hospital and also to get other services and treatment after laboratory investigation. In order to provide these services first health professionals should work on early identification and they should give emphasis. Even if the service is being provided starting from the community by health extension workers, it does not get focus due to the limited and short training they had.

Currently, for us this is included as indicator to evaluate and monitor the program after revised HMIS. Its new and we have no experience previously. Currently it’s a new indicator and we hope it will be improved as days go by.

3. If it is indicator is there any set of targeted children to be screened, to link developmental delayed children and how is our progress on that?

**Answer:** may be for next time there will be target but I did not see any still know.In a year all under five children in the zone are eligible and those children who are ill and come to the health facility having diarrhea ,pneumonia or any other case . There is a set of target to treat pneumonia or diarrhea so under five children are eligible for the service. We do the coverage by classifying this much number of children have confirmed developmental delay and this much number of children have suspected developmental delay.

Target is our plan and the plan is under five children. We work to achieve the percentage as a coverage. This is how we work, may be our target is set as a plan. This is being considered currently may be this is why its not included under strategic plan or other plans.

4. As you already mentioned the service is being delivered at health facilities only for those child who visit sick baby clinics what is the policy or program related reason for this? Is there any plan to provide the service for those children who did not visit the health facility?

**Answer:** Even if it comes as one indicator now, previously we have been working on nutrition related activities to solve nutrition related problems. However, we did not see how many of the children have confirmed developmental delay or suspected developmental delay and the need to see this comes now on HMIS may be this in last year or the year before. So, the work starts after ward.

Currently most children who had developmental delay in the community did not come to the health facility even if they have confirmed delay. Currently, only those children who are sick get the service as initiating the service. Next time based on government direction the screening will be given as nutritional screening.

Personally I believe that the screening is necessary but children don’t get the screening. This children are moving like a healthy child and they don’t say I am sick or something. This problem is identified by health professionals and also mother bring only the sick child to the health facility. The health professional follow their susception to ask question about the stated milestones and in order to screen this it takes time . But currently the program is on its beginning and children in the community are not well addressed only those who are sick and visit the health facility access the service.

5. What are challenges or barriers to provide the service?

**Answer:** The first thing is that if it is considered as indicator ,health professionals starting from the lowest to those in health center should take trainings in a organized way. Chart booklets in the previous time is prepared in English like in ICMNCI.ICMNCI is given at the community level and its prepared in Amharic. But the government did not prepare it in Amharic currently. However, the activity exist under reporting formats. Developmental delay data exist at the health post level in a form of aggregate. the health professions should take this training but what they take was orientation on what is the need of child developmental assessment? What is its importance? Why we invest on that? They take a highlighted information on that during the IRT training. In addition, there is a gap of training on identification of those children who have confirmed DD, suspected DD and no developmental delay and then to take action on that.

For health professional working in health center under five clinics training should be given every time. But to give training few peoples have TOT at Regional level but not at Zonal level. This training should be given in a well-organized manner.

In addition, the service should not only give with IMNCI as mentioned earlier but there are nutritional interventions to prevent developmental delay in the first 1ooo days. However, there is nothing done to identify how many of the children have the problem and training was not given. The reason for this is shortage of budget to give training on the above-mentioned points. Those official in regional health bureau should give training based on demand, giving training for few individuals could not bring that much change. As professionals treat pneumonia, diarrhea or as they screen and manage malnutrition, they should get training in order to work by identifying those children who have developmental delay and to see the developmental progress of the child and to identify whether the child has good development or delayed.

Professional also should have to take responsibility and be committed to identify the problem. However ,in order to do this work emphasis is not given by the regional health bureau ,limited trainings are given on IMNCI and assessing, classifying development is included under it. A detail training is not given.

The other barrier is the higher official do not give attention so we face shortage of budget ,lack of trained professionals, book lates and professional at the lowest level don’t get the training. The chart booklets don’t involve the community and health extension workers. The professional should also take responsibility to assess and do the work at all level.

There should be demand creation and the community should get awareness that it does not means all children who walk have no any problem. children can have developmental delay we do not know till they are screened. Therefore, in order to do this activities the government should give solution for budget related problems .The attention for the problem is given at regional level but at other levels much attention is not given. If those at the lowest level are not involved there might not be success. Even the community should involve in the program. Preparing and sending module and giving training for 40 minutes only is not enough. This is what I say.

6. What are the other challenges related with infrasucture to provide the service?

**Answer:** In order to assess and classify this we don’t need other things. There are milestones which already exist on the chart booklet. The child will be checked based on the milestones set on the chart booklet for each specific age groups starting from birth to at one month, four month,6 month,9 months, etc. and the expected development for each age group. If the child does not full fill this the child has the problem.

There are other activities like nutritional interventions like nutritional screenings by using MUAC which help to identify their nutritional status and other visible problems, therefore working these activities simultaneously by using the chart booklet for identification of whether the child has developmental problem or not.

There is no other additional infrastructures or material for this purpose. If we don’t get any other additional material for identification in the future, currently professionals use to identify developmental delays by looking at the expected milestone on the booklet.

In addition, we are familiar with working by going up and downs in rural areas. we have been working by walking long distance.

7.what about the challenges with the availability of materials for developmental assessment and working environment?

**Answer:** Maybe for what you say now those children who are malnourished ,when they get their treatment they play in the facility and providing them material to play will help them to develop well because malnutrition children are damaged in multiple ways. In the facility malnourished children play by using the pictures on the wall . This is what exist in the facility but what I mentioned earlier as a problem exist at the community.

I thought the question was about the infrastructural problems that exist in the community to identify the children who have the problem. The materials you have mentioned don’t exist in all places. I don’t know if you see some drawings on the wall and playing materials specially in EPI rooms. For malnourished children at stabilization centers and those in the community there is no playing materials. out patient children come to the stabilization center once a week to take the available food or planpy nut and return home. Its not questionable there chance of this children having DD. But children whose nutritional status is not identified have the chance of having developmental delay. However, at community level there are no material to play with. At facility level for example at health centers, hospitals pictures are drawn and paying materials are available in limited facilities and these things are not available in all health facilities.

7. Still now we have discuss the barriers for in implementing child developmental assessments, what would be the possible solution and the responsible body?

Answer: The responsibility is shared starting from the government . The commitment should start from the higher official. The consequence of developmental delay is known and the government starts to set direction. What is started should be implemented on the ground. The government should also give attention and integrate the service with other programs. Emphasis should be given by government starting from the higher officials to integrate with other services and provide training.

Everyone is responsible for this starting from the ministry, regional health offices, zonal health offices then to community. Every health professional and health extensions are also responsible and we should have to aware them what is expected from them .we have to tell them they should have to work on that. Everyone has a responsibility to improve and change the service .This means we have to support by providing resource, training and other things like what we do on other programs and it’s a responsibility of all of us. Those individuals at lowest position as well as woreda level and zonal level is responsibility. If we done lead them and aware them as a program those at lowest level will ignore it. It should be handled as a program. At this level everyone is responsible.

8. What are the facilitators to provide child developmental assessments? what are the good practices at the policy program level?

**Answer:** The first thing is, already the system exists so its easy to make this a program. If you say what? always there is nutritional screening. we can work, screen with that then provide treatment as you asked earlier we can work by full feeling the infrastructures, playing materials and other logistics in the service.The presence of the system is one thing.

The other thing is the presence of different stakeholders to support this. World food program and UNCIF haven working with government to prevent developmental delay and after the problem occurs.

The government also put this as an indicator and there are already established initiatives as a system. There is also reporting. This is very encouraging. It is inserted as indicator which make it easy to measure and to provide the service continuously and meet the children. This all things are supportive.

9.If you get the chance to improve the service what things you will change and what things should be given to increase the accessibility of the service?

**Answer:** First, in order to provide the service in a well-organized way and to screen all children timely and to give action, we have to identify the challenges and solve them. Specially we have to give training for health professionals then create a way to identify children from the community . In addition, we have to full feel different infrastructures and provide treatment in the health facilities . We should also provide treatment at all level , doing only the screening is not good.They should get treatment at health center and hosipital level.Logistics should be available.

Everyone should be responsible for program and it should be strengthen. Putting it as indicator is not enough.it should be considered beyond that because the consequence of developmental delay is large .so handling it in a well organized manner by government is important as a country.

**Thank you**
