# Supplementary material for: Why are the implementation of and access to child developmental assessment within routine healthcare so difficult in Ethiopia? A descriptive qualitative study
Source: PLoS One. 2026 Aug 10;21(8):e0355686. doi: 10.1371/journal.pone.0355686 (PMC13456246; doi:10.1371/journal.pone.0355686)
Supplement: Supplementary file 2 [file pone.0355686.s002.zip › data/Transcripit 10.docx]

**Transcript 10**

**Interviewer:** Are you willing to participate and your voice to be recorded?

**Respondent:** yes, I am willing.

**Interviewer**: to begin with this, as a pediatrician what are your routine activities here in this facility?

**Respondents:** ok, good here we are doing many activities. Here we have intern students practicing and medical doctors as staff. Once they assess a patient if necessary we consulted for decision on treatment. Usually, cardiac-related, Neurological conditions, syndromes, severe infections, neonatal, and other severe forms are few in need of our involvement as a senior. And we have follow-up cases at OPD like seizure and cardiac cases. For those with active seizure, shock, and severe pneumonia, we managed at an emergency room. For those admitted cases with clear indications, we look at wards.

**Interviewer:** when I came to the research, what does a child's healthy development mean for you?

**Respondents:** when we say a child's health development, it is not merely a size or weight increments or decrement. There are other issues considered as mental development, attitude, talking, language, communication, peer interaction, social interaction, and ways of solving problems that need to be considered.

**Interviewer:** what is your experience as a pediatrician?

**Respondents:** I have 16 months of experience as a pediatrician and I served 4 years as a medical doctor before specialization.

**Interviewer:** with this experience in the Gamo zone, which type of developmental delay do you experience?

**Respondent:** many times most births end up with healthy outcomes, sometimes perinatal asphyxia might happen which leads to the development of cerebral palsy which in turn to developmental delay. This is common in developing countries commonly in preterm where ANC follow-up is not strong. The perinatal asphyxia might occur before labor, during labor, and after labor. Before labour, those having HTN-related problems need to be managed to prevent asphyxia. During labour conditions like fetal distress need to be followed and managed accordingly to prevent this. After birth those who need resuscitation need to be assisted early to prevent it. If we treat early we can prevent damage to the brain. Each minute has its own meaning.

**Interviewer:** which type of developmental delay do you experience? You tried to mention earlier but I want to know another type of developmental delay.

**Responder:** from my experience, the most common developmental delay is related to cerebral palsy. The other is related to hypothyroidism, in most developed countries newborns are screened immediately after birth and managed accordingly. However, screening is not common in developing countries. There is also developmental delay due to rickettsia which results due to lack of sunlight. There might be also downs syndrome.

**Interviewer**: you told me the causes of developmental delay. From language, cognitive, and physical gross motor delay, which one is common?

**Respondent:** developmental milestones include: gross motor, fine motor, language, cognition, and social. The common is gross motor-related delay. Most children have this at 1 year of age and afterlife. Those in rural areas are late to come and usually after a year. hoping that the child can get better.

**Interviewer:** at what age those with developmental delay visit health facilities?

**Respondent:** mostly visits at the late stage

**Interviewer:** How is developmental assessment being assessed here in your institution? What related services are provided here in this institution?

**Respondents:** mostly we do even if not normally included in treatment guidelines. From education, and experiences, we ask gross motor related issues like when started sitting, standing, walking and we compared with the age standard. We also assess fine motor related like when smiling started, playing with peers, and the like. The problem is even if we diagnosed fine motor-related delay we don’t have the treatment here. For motor-related delay, options might be taken like physiotherapy, referral to Addis Ababa to bring neck support, and the like. Most are not available in our setup.

**Interviewer:** important material missed when you want to support such cases?

**Respondent:** prosthetic materials like head support, and back support (like a belt applied at the back), are important in supporting and increasing muscle strength. And kits like testing hypothyroidism, and rickets testing are important if available.

**Interviewer:** do you believe the service provided is adequate and timely?

**Respondents:** not adequate. Treating developmental delay needs many things like multidisciplinary staff including physiotherapists, speech therapists, and pediatric neurologists.

**Interviewer:** for whom developmental delay assessment is being done? Most children visit facilities for illness or immunization in most cases.

**Respondent:** for those coming for immunization this assessment is not being done. Usually for those at OPD, emergency and at-ward developmental delay assessment is being done.

**Interviewer**: do you think, the assessment is important for all children?

**Respondents:** yes important to address for all. But practically we are doing for those coming with complaints.

**Interviewer:** what do you think about not addressing all?

**Respondents:** I think lack of awareness for providers in the immunization room since doctors and specialists are not assigned there. In follow-up clinics many cases are there making busy staff.

**Interviewer:** once diagnosed how is the follow-up?

**Respondents:** once diagnosed delay, follow up is based on the parameters for example if having difficulty in supporting head the follow focus this. Sometimes underlying causes may be treatable like hypothyroidism, and rickets, and evaluate the changes.

**Interviewer:** How is data handling during follow-up?

**Respondents:** registered in card either part of a complaint or physical examination

**Interviewer:** what are the barriers at health facilities?

**Respondents: There** might be a lack of political attention at the top level. Knowledge and skill gap at provider level. Sprit-related beliefs and lack of awareness are barriers at the community level. Financial-related barriers also play a role as tests are costly for example for cerebral palsy MRI is required which is costly and also costs related to thyroid testing. Political commitment is paramount important. If political commitment is there the structure share to down which forces implementation in every health facility.

**Interviewer:** what are the enabling conditions we have now?

**Respondents:** some causes are treated like vitamin D is available for rickets and hypothyroidism. And now subspecialty programs are expanding which contribute to treatment. Human power is now increasing like pediatricians.

**Interviewer:** if we develop standard screening tools and manage in facilities, what do you think are the challenges?

**Respondents**: as I have said some are easily treatable. But others like cerebral palsy need multi-disciplinary. The benefits are related to facilitating early diagnosis. Late diagnosis prevents the chance of reversing the cause.

**Interviewer:** Have you ever been diagnose developmental delay? How the families reactions?

**Respondents:** yes, most cases visit facilities believing a delay in sitting and walking. Once diagnosed we tell the treatment options that might be psychological, or define treatment. For those with financial capability, they continue to follow but others fail. The awareness level is better for urban families. Many options are used especially from the rural side like holly water, and traditional healers. Most Childs are accompanied by their mother. Some services accepted for which they see as effective and have their involvement include physiotherapy. We teach how the practice of physiotherapy and when the saw affects the feeling of happiness. And they eagerly ask when their child recovers.

**Interviewer:** understandings of the provider with the occurrence of developmental delay?

**Respondent:** most can understand. For the below inters, they are not aware. Special training is required to fully understand the cause.

**Interviewer:** Thank you
