# Supplementary material for: Why are the implementation of and access to child developmental assessment within routine healthcare so difficult in Ethiopia? A descriptive qualitative study
Source: PLoS One. 2026 Aug 10;21(8):e0355686. doi: 10.1371/journal.pone.0355686 (PMC13456246; doi:10.1371/journal.pone.0355686)
Supplement: Supplementary file 2 [file pone.0355686.s002.zip › data/Transcripit 11.docx]

**Transcript 11**

Interviewer: How is child development monitoring being implemented in this health facility?

Answer: When they come to us, after they take out a card. Then we will ask them about the problem and why they came. Then we will measure their weight, take their temperature, then we will take their height and age, and then we will do an assessment. If we see what the problem is, then if it's a cough, we'll write down how long it's been while coughing, and then we'll count their respiration. After that, we will check how much is it. we put and check whether it is appropriate for their age or is it high or low. Then there are diseases called under five top diseases. If they exist, we fill yes or no on the registry. If there are cases to be sent to the laboratory like diarrhea, we will send them. we continue asking after their return from the laboratory, then if it is not under five top disease 5, we will fill in other. Here, we put the classification in the record and we give treatment, then we give diagnosis, we give follow up. Then we ask the developmental milestone. After looking at age and weight, we check the weight respective of the age then see whether she was good previous or has lost weight, by using weight scale. if she lost weight, we check on which month she lost and why she lost weight, is that due to illness or not. we can see that it is not going well with her age.

Interviewer: Okay, you are telling me what you do the assessment by using IMNCI guide. Do you think this guide help you to pick developmental problem?

Answer: Yes, what is on it will be useful if we ask and do it properly. The family will also be happy. If we tell them such kind of things, they will be happy. For example,’’ this is the child's age. he should do this at this age. Now, the decrease on his growth may be related to the illness and he needs to be monitored. Now we have prescribed medicine for this illness. When you come back, after two days or a week we will see the weight again’’. If she come after a week and she say that the weight is better now, and we check that and send her home.

Interviewer: let me make it clear , in order to assess developmental milestone for example whether to check a child starts to smile at the age expected from him or not. Do you think the assessment way is sufficient to identify the problem?

Answer: okay, it is not normal at the current stage to identify children's having development, that is, their cognitive development or the actions they show in one or two months. We have under five OPD but normally if the pediatric has its own room, it is convenient to monitor that. But currently it is up to under five, so there are gaps. These gaps are normally we do not admit children below two months unless they are sick. In order to check that for example if a child can smile, identify thing below two months, we have to examine them in bed but there is gap regarding this. In addition, now there is a case flow and this again causes a gap. But it is good if it is practiced

Interviewer: How do you involve families in the child developmental assessment? Do you provide counseling on how to monitor child development?

Answer: Yes, if a family comes to us, we will ask how they are breastfeeding. There are some children who live up to two years of age, do not take any food, are only breastfed and have good score on the growth monitoring. Their weight is going in line with their age. Also, what we do with the others whose weight is lower than the expected at that age, we tell them that she is not breastfeeding well and it is a sign of less follow up, so she has to keep the monitoring.’’ You have to monitor the baby. We ask her how many times within a day you are breastfeeding. We tell her that you should have to breastfeed 8-12 times per day. We tell her You have to feed the other breast after the baby is done with one and you don’t have to give one side of the breast because the baby is crying unless it is finished.

Interviewer: Thanks, just to make it clear, do you tell the mother /caregiver to observer what the child is doing what is expected at his /her age till the next visit. For example, do you tell the mother of one year baby to observe what is excepted from the child and what she should observe or you only give counseling on breast feeding or other nutritional problem?

Answer: ooh there is gap on that. Those professionals who had the training, not every one give attention to that. Normally, there are individuals who come and ask us about it. There is gap between professional based on whether they had training or not. The other gap is if only one person work in that area it will be a burden. Of course, for those children who came to us we should tell about it and ask what kind of things the child is doing ,what did he show should be asked. There is a gap in asking to know whether he struggle to sit in one year, does he turn around when you moved things around, does he tried to talk ,can he move here and there, does he try to stand holding objects

Interviewer: How do you provide a continuous developmental assessment in this health facility? What you are going to do if you find a child with developmental delay and how you involve parents? Did you face a challenge related to this problem?

Answer: So far I did not face a child with developmental delay while working in rural settings but in under five clinics at the urban areas there are shiny materials and when they see it children may show something suddenly. when they come to us as they are normal and we refer them if we suspect some thing and tell the mother he should be seen. The mothers can be shocked while they are being examined at the referred site. If we tell the mother the child should do this things but he is silent and this means he has some disease or his development is not right, she will be shocked. So we tell them as ‘what you should do is to attend the follow up, play with the child, you have to play with child while he sleep, you have to support him to move and in addition his eyes should turn with you equally”….we teach the mother by telling this things.

Interviewer: would you please tell me what the barriers are to provide the assessment? Please tell me the barriers related to providers or the healthy system?

Answer: yes, of course there are experts who had training on that. But even if there are trained experts they are few in number in the area. if 1 2 3 individuals are trained, we can make a shifts every 8 hours, and it can be done well on a weekly basis. If there are challenges, it is possible to gather and talk about the possible solutions. Normally, there were trained professions on the old one and some were shifted to other places ,I am the only one who had training on the new one. while I am working at that unit there was high burden of cases so I shifted to other places, I return to this unit because the burden of case is high. I was asking …. give me a one-month break and I want to go to other regions… still the case is high. And in under-five if the number is high in middle there is a challenge otherwise there is no other problem. Orientation is being given for professionals but some work by giving emphasis and others do not.

Interviewer: you mentioned one barrier as few trained staffs, what about the other barrier you encounter to provide developmental assessment?

Respondent: yes, off course, the room is not like the previous one. It has been a year and some months since we come to this new health center. when we were at the old health center it was good. This one has some ups and downs and when clients come it cause some confusion. The rooms have their own beds to do examination. The height and weight measurements are also available. The unit lead this but material which are important are not available. Its is good to have a demonstration place but it’s not available. we can do demonstration for 30 or 40 minutes for each child, we can even use these materials if available for children having developmental delay.

Interviewer: you told me that there are no materials, what support you are receiving from the managers.

Respondent: Regarding him, he has no problem. He is very willing to do whatever we ask him. He is cooperative and willing to do anything. if there are materials at the store, he let us to use it.

Interviewer: what other barriers are available regarding time?

Respondent: we work for 8 hours like any other place. Our shift is every 8 hours expect for the emergency rooms which works for 24 hours, the health officers and nurses are responsible to work there.

Interviewer : sorry to interrupt but the question is regarding the time to do the assessment?

Respondent: okay related with it, to do the developmental assessment it takes time .For one child 20 or 30 minutes is needed. We are not using such time to do the right assessment due to the high number of case flow, we do not use the appropriate time. If the room is two or there is a few number of cases we can do that but we fail to do so.

Interviewer: Do you think the guide you have know is enough to do assessment for each domain?

Respondent: we need additional material and its better if the materials to be used is written by bold and put in the room . The manual we have is the IMNCI guidelines and we use it because it describe the age with the responsibility.

Interviewer : How could we solve this problems?

Respondent: As I told you earlier we discuss this issue with the manager. we discuss about what we encounter every week. But the biggest challenge for us is the health insurance thing. Individuals come to us, we have to examine for 10 to 20 minutes and we could not say you have no any disease and discharge/return the patient, it’s very difficult for us……………(could not hear )

Interviewer: What do you think of changing the current mode of developmental assessment to developmental monitoring by using screening tool?

Respondent: If such kind of thing available we can identify the problem early. The cause could be due to feeding problems or due to lack of knowledge on how to support the child to develop well. If this thing is available, we could identify the problem early before it gets complicated whether its stunting or other problem. So, this service is better. I think it helps to provide the service early and can make them to come to the health facility.

Interviewer: So, this is the benefit, so what would be the possible challenge for this?

Respondent: The challenge could be if we go to home to home to and say your child has to do this thigs at this age but he/she fail to do so. The parents could say and resist ‘who are you to guess this’. The community could challenge you when you do such kind of things. So you have to develop a good gate in strategy. You have to ensure them that you come to provide so many services not only this one. You could not let the community know why you went there directly. You should have to go through so many steps. They have the fear of being stigmatize by the community if once you tell the mother your child has this problem so they challenge you to receive such kind of service.

Interviewer: what could be the challenge related with the health care system?

Respondent: Related to the health care system, the focus is providing urgent activities. If somebody is assigned to provide this service and some urgent disease condition happen, the professionals stop working and shift their focus to that. So, it will continue like this but if it’s a duty for one individual and if one individual is assigned to work in that area, they will engaged on that fully. But if individuals are assigned temporarily to work on this together with other activities like family planning and some urgent activities come all individual would shift to that .so the program could stop. This are the possible challenges.

Interviewer: what are the possible facilitators?

Respondent: okay when I start from facilitator factors for example if you assign professional to sites, they need something for example they need money to go to site. If there is nothing to motivate them, they do not fully engage in the work, even if they can go to the work place, they do not engage on it. If you assign professionals and there is budget for it, it will motivate them to engage on the work.

Interviewer: what will be the possible direction you suggest to have a continuous follow up?

Respondent: In order to provide a continuous monitoring for example if we take children who come to receive EPI service, they come to receive the immunization service whether or not they are sick. So, we can proceed the follow up even after the children finish the immunization schedule. When they come, we know who finish the immunization so we will provide a health follow up whether or not they are sick or they had immunization to take or not we tell them to continue the follow up for some time in order to do the assessment. If we made a plan and start our activity, our community could not challenge us to receive whatever service that we told them benefit there child.

Interviewer: what would you do if you identify the child has developmental problem?

Respondent: if we reach at the diagnosis of the child having developmental delay we start searching for the reason that cause it. It could not come by it self because when one child had developmental delay it could be related to some illness or nutritional problem. So the solution is to look for the reason or the cause for the problem.

Interviewer: what kind of service you provide for such kind of problem?

growth retardation based on the weight measurement.

Interviewer :sorry can you tell me in relation with developmental delay?

Respondent: I could say anything about that because I did not get training on IMNCI .so I do not know about it. But in this facility what we provide is identification service not treatment service.

Interviewer : what about the other barriers?

Respondent: currently there are a few number of professionals around 4 or 5 . Two of them are assigned permanently in this OPD. One work at AM and the other on PM. At the night shift children may not meet those professionals who had training because there is a shift. I raised this question that professional who had training should be permanently assigned at duty time. its better for the children to get the service at anytime from individuals who had training because the IMCI guideline also suggest that.so we discuss on that and decided that in the next budget year professional to be assigned permanently on AM,PM and night shift schedule and to be available at anytime. So this will be arranged for the next time but for now there is no service.

Interviewer :can you explain what the emphasis of the health care system for developmental assessment?

Respondent:It is a service that has not got emphasis, the system focus on immunization, stunting but not on development.

Interviewer :what do you think the reason is?

Respondent: I think that in our community the case is not common ,it occurs occasionally. But the condition is occurring in a more frequent way now a days. I think from the first place the problem is not identified or not studied well. However I could not say it does not available in the community. But I perceive it is not a well studied area.

Interviewer :what is your suggestion to improve the current service?

Respondent: The first thing starts from the room that the under five OPD should be separated from immunization. There should be a comfortable place to examine the children. so EPI and under five service should be separated. The other thing is in the next buget year we have to consider budget for the things necessary to provide such service. Also, professionals should be assigned in the outreach to facilitate the service provision. So, the managements should consider and decide whom to assign. The other is to work with the health extension workers and educate them in order to collaborate in so many activities.

Interviewer: Thank you
