# Supplementary material for: Why are the implementation of and access to child developmental assessment within routine healthcare so difficult in Ethiopia? A descriptive qualitative study
Source: PLoS One. 2026 Aug 10;21(8):e0355686. doi: 10.1371/journal.pone.0355686 (PMC13456246; doi:10.1371/journal.pone.0355686)
Supplement: Supplementary file 2 [file pone.0355686.s002.zip › data/Transcripit 12.docx]

# Transcript 12

Interviewer: introduced ourselves thank you for your willingness to be interviewed. We will have few minutes of interview with you may be not more than 30 minutes. How old is that child? Four month old.

Response: okay.

Selam: no we will be talking about the four years old child not the younger one.

Interviewer: okay. Is he four year old?

Response: yes. She female

Interviewer: okay. Is she female? How many children do you have?

Response: three children

Interviewer: three children, with this child?

Response: Yes

Interviewer: where did give birth for that child (the four years old child)

Response: Her in Arbaminch,

Interviewer: hospital or health center?

Response: Arba Minch hospital. They took me there due to difficult labor

Interviewer: okay did he vaccinate?

Response: yes; he already finished (the little child crying)

Interviewer: okay. You said this four years old child is unable to speak?

Response: yes! He cannot speak, he cannot stand up

Interviewer: did you try to go to health facility for medical attention?

Response: yes. I do. I follow there in Arbaminch hospital. He is receiving drug. He has nose bleeding, dizziness.

Interviewer: is there nose bleeding

Response: yes.

Interviewer: did you go to medical attention

Response: Yes I tried but no change.

Interviewer: you said the child is unable stand up

Response: yes, he cannot stand up, move, do not eat independently

Interviewer: when we say there developmental delay for one child. What are the manifestations?

Response: I come here foe follow up they said there follow up: my problem is breast milk very small breast milk.

Interviewer: we are not talking about your problem, but the signs of developmental delay you can observe on the child.

Response: one child should walk at age one, “year should not end before I stand up”. This did not stand up at age one, people said child can stand up after one year; it is not such serious problem. I accepted their opinion but still she did not. After two years, passing saliva via mouth started. Unable to eat food, sit on the ground. she disturb. When I follow treatment they said during delivery she got some harm.

Interviewer: was it by operation

Response: no it was by labor but, it took long on labor, she drunk amniotic fluid

Interviewer: one you’re said how you said one child has healthy development and or not healthy (phone ringing). Sorry. What characteristic should healthy child show?

Response: healthy child sit down at age of seven month, at age of 8-9 month crown, and at age of one year child should independently stand walk. Some children can walk at age of nine months. Healthy child walk at age of one year.

Interviewer: did have information that developmental delay is problem in this community?

Response: me

Interviewer: yes you

Response: the first child walks at age of one year and four months. It was the first child, but the second child remains on siting down.

Interviewer: now when we say the problem, there is speaking problem, walking problem…and the like among those problem which one is the common problem?

Response: it was due to lack breast milk, and food. Lack food and generally care.

Interviewer: which one is the common one. Is it unable to speak or unable to walk?

Response: due to lack care, children fail to stand up early due to short coming of in food (phone ringing)

Interviewer: in this community to say there is developmental delay or not what are the common signs seen on children?

Response: there is abdominal pain, diarrhea, cry simply, emaciate

Interviewer: when we say the child has developmental delay, if you know it where did go first?

Response: we bring to health center, w show there.

Interviewer: after how long stay (duration) did you say the child has developmental delay? Is one year, in two years or after how long?

Response: on my side I took to the health center after two years. People say child can stand up at age four or five do not worry, I was waiting for it, but she begins falling down, dizziness, and salvia passing via mouth. Blow out her eye, (7:24) (child’s sound), therefore, I think there is some problem; I took her to health facility.

Interviewer: you took her to medical attention at age two?

Response: yes at age two. It was due the health problem, not for the developmental delay, she was sick. If was not sick I would not go to health facility.

I: yes!

Interviewer: When child is unable to speak, unable to stand up (child crying), what do other people say, when is the time to stand up and speak?

Response: some people say at age of two, if there is no problem on eating, and healthy it is not stressful. Their health conditioned hampered is the main problem.

Interviewer: have ever heard about developmental delay, its diagnosis and care?

Response: I have not heard about it.

Interviewer: you do not know about it? (*Other women taking about vaccination, and greeting*), how did you cope with the condition? How do you feel? You said that you don’t have information about developmental delay diagnosis and treatment. What do you feel about developmental delay diagnosis and treatment treat what do you say? How should look like?

Response: on my side I say, I say if children fail to stand up, there if some problem, unfulfilled thing. If the child recurrently become sick, there should be follow-up, they should quickly seek medical attention. Those with capacity (economic capacity) can feed their children and make appropriate follow up.

Interviewer: you say there should be follow up.

Response: yes! To they should ask why the child fail to stand up at the appropriate age?

Interviewer: which part is it about unable to stand up or unable to speak? When should the start the follow up?

Response: something if they are unable to speak at their appropriate age, some people use traditional medicine for those unable to speak.

Interviewer: is it medicine

Response: it is something…..fruit/cereals, (shimbra, and the like )

Interviewer: is this make them speak out or treat the problem of unable to speak

Response: they told me to take this solution, but I didn’t. One chickpeas (shimbra) barely, wheat, cereal, maize, and all cereal type seven. Feed the child, no one should observe while doing this, then the child will immediately start speaking they said. (Phone calling)

Interviewer: okay! Eheheheh….now did your child made medical checkup about developmental delay?

Response: in the very time, at the time birth?

Interviewer: it can be others or the child with developmental delay problem. Did they make any medical checkup about developmental delay?

Response: they didn’t

Interviewer: including the other?

Response: yea they didn’t

Interviewer: why? Is there no service?

Response: I did not inform them. I did not disclose.

Interviewer: including all the other children?

Response: yes all the children. Children walk by their time. There no problem, the priority is health, if there is no disease at four or seven children can walk, they said, and then I keep waiting. But this child has weak body, unable to stand up, and I don’t think she will stand.

Interviewer: now child with developmental delay, what was the first sign that made you suspect for developmental delay?

Response: first, there was seizure, blow out her eyes, begin to fall down, foaming (passing saliva through her mouth), and then I suspected for other disease. Therefore we started medical attention. As the healthy child she did not tried, supported by chair, she did not tried it. She remains as she sits down.

Interviewer: what did the family members say?

Response: the family members hesitated; there might be problem during birthing, and got the follow up chart (paper). There was problem during birthing, it was head nerve, they made us to start follow up, and saying there was head nerve. It was about one year, seizure stopped at that moment. Now unable to stand up, unable to feed herself, and our main concern

Interviewer: what are you feelings on her?

Response: what

Interviewer: your feeling on her, she is behind her age group, she sick…?

Response: we feel much, we cry always, her age group started education, but she tied in the house, we cry a lot. All her age group started education at age four.

Interviewer: I did you ever seek help/aid?

Response: actually we have no capacity (to mean economic capacity). We feel if she is cured, awe think a lot but we did not steeped forward.

Interviewer: is it only idea or did you tried any steps?

Response: meaning?

Interviewer: just seeking medical attention, or trying treatment. (Child crying sound)

Response: we did not. We have not tried anything aid.

Interviewer: why

Response: actually, if someone shows directions, in the kebele the registered those with disability, but nothing come after the registration,

Interviewer: what hindered you from seeking help from kebele or else bodies? What is the stronghold?

Response: nothing. If aid is available we are willing, during the registration we made her registered. They go and no come back

Interviewer: you only informed kebele, did you try medical attention?

Response: we did not tried.

Interviewer: do you think that there health service for this kind of problem

Response: yes

Interviewer: what is the big problem that make you not seek treatment?

Response: ehehe. We go to Arba minch, they treated her.

Interviewer: to Arba Minch?

Response: yes

Interviewer: what did them said?

Response: they said it is head nerve, she drunk amniotic fluid (meconium aspiration). She did not get adequate treat at birth. She stayed at warming room (to mean NICU) for fifteen days. I was there for fifteen days in the hospital waiting room. She did not treat adequately at that time. It continued as she grows older and older.

Interviewer: what is the facilitating factor to seek medical attention?

Response: we wait by hoping she will get better.

Interviewer: to seek treatment, or aid what motivate, or facilitate you?

Response: we wait by hoping she will get better.

Interviewer: searching aid and seeking treat, you didn’t think?

Response: we think it.

Interviewer: what hindered you to take one step to treatment?

Response: they told us, take her to where her birth takes place, we go there. There is no change.

Interviewer: no change

Response: yeah! No change. They go to better place, higher treatment centers. For this we have no capacity (economic)

Interviewer: who said go to better place

Response: they said

Interviewer: who are they?

Response: hospital, you should better go to better health care, we have no drug for her they said.

Interviewer: is there any condition that hinder you for stepping to better health.

Response: it is capacity (economic)

Interviewer: you mean capacity to mean money or economic capacity?

Response: yes. It is to mean money.

Interviewer: how about culture? Customs?

Response: no problem, it is only capacity

Interviewer: on your side (family) what difficulties are there to make follow up about development?

Response: as I told you it is capacity. Preparing balanced diet is difficult for us.

Interviewer: not just to say what you are saying? It is about early identification of developmental delay at eight month or earlier. Whether you have interest or not to make developmental assessment.

Response: this is when at age of fifteen days the child should be exposed to sun light, massaging, breast feeding, and make good sleep. Without doing this if the child grows up to four moths…

Interviewer: regarding age-appropriate developmental assessment follow up, what hinders you from seeking developmental assessment? What facilitate it? For example ,in health center or hospital there is a milestone, which describes four month child’s character, eight months, one year and the like developmental stage or levels. If the child fails to show that character the milestones level the child as hampered development or growth. For this there is follow up in the mention health facilities. Is there anything that hinders you or make you follow it?

Response: nothing

Interviewer: so, why you didn’t follow?

Response: just it is peoples opinion, some people say child might stand up at any age, some others asked about what happened to the child? What can I say, it not infection, like common cold, what can I say? How can I say the child is unable to crown, unable to walk…or else how could i? I think could health center accept me or not. What should I do? Wondering in this dilemma I left behind.

Interviewer: you left behind; you did not try bringing to health facility?

Response: yes I left behind. You know sick people come to health facility. How could I bring walking problem as medical problem? What could people say?

Interviewer: yeah. This is what you think?

Response: yes

Interviewer: okay ehaheheheheh..get out of this kind of thinking and to go to health facility if the child is unable to crown at age eight months, and unable to walk at one year of age, what motivates you and what hinders you?

Response: the motivating, we stressed, what happened to my child. Is it disease? And the like questions are the common questions.

Interviewer: is there any other children in the community you know with such kind of problem? Is there any cultural or social connections?

Response: yes, in culture, children stand after pronged stay, mostly tin children stand early (child with weak body (tin body)). This is Local recommendation, family related conditions can be manifested on standing early or not. This cultural advice from local people. Don’t worry. It’s due to weak body or big body (tin and fat body). If the father or mother is fast, the child is fast too, if not the child is not too. It is family related condition.

Interviewer: in the community, family and individually you say there is no barriers and facilitators to go to the health facility to make follow up about developmental assessment?

Response: yeah it is only advice from people.

Interviewer: is It only advice from people?

Response: yes! People advice different things some of them are good and some others are not significant. The child can stay long before she/he stands up., every child is not equal, they beehive differently. We accept this advice and lag back.

Interviewer: now we discussed different issue relate developmental delay, you already understood it. What do think in the future about developmental assessment service utilization? Are you ready to make follow up?

Response: yes! I accepted your advice. First I lag back due to local advice. It was my fault, i could have made follow up. This is mistake. I hurt my child, I will make follow up in the future.

Interviewer: is there any other idea

Response: yeah! My breast has no enough milk, is it the problem? Naturally my breast has no milk; I think this could be the reason. Why my children stand up early, they stay long before they stand up.

Interviewer: the others have similar condition?

Response: my first child took one year and four months to stand up and walk. She (the first child) was by operation, this one is by labor. She was harmed by labor.

Interviewer: do you have knowledge about the existence of developmental assessment service in health facilities?

Response: no I have not.

Interviewer: there is service of developmental assessment which is called growth monitoring or it can be named developmental assessment. If your child leave behind his/her age inform the healthcare providers. The service is in health center of health post or they can link you to the service. You need to make follow up. Okay?

Response: ok fine! I will do it.

Interviewer: about the service in the hospital, what liked and what she dislike?

Interviewer: yeah! She reminding me about the NICU service, you said you were admitted to hospital at the time of birthing

Response: yes

Interviewer: from the services delivered for you at that time, which one did you like and which you disliked?

Response: they give me care, they were compassionate, but during the lobor they drugged the baby’s head by applying instrument. (Forceps application)

Interviewer: ohoo! Was it on the head?

Response: yes. They put instrument on the head and drugged it out by force. Then they hided the child, I have seen my baby after four days. At day four one health care worker come to me and said are you Merkeb? I said yes, what happed to your baby’s head? She said. I have seen wound on the head. I said I haven’t seen my baby. They did not show me. You did not see? I said yes I haven’t seen. Please show my baby if she is alive or tell me if she is died, please! Don’t hide please! Then she said I will took you there (NICU), she took me there. Then I have seen her head was open wound, deep and wide wound, I feel so sad, and then I stayed there for fifteen days there in the hospital. They providing care for the wound and there was follow up. Then after fifteen days, they said now she is in good condition, they checked different things. Then they discharged me and my baby and told me go to home. Then it was fine, the problem come after two years. She falls sick and I come here (Lante health center), they told me to go to hospital where I give birth. Then I go to the hospital, the watched the previous note, and said there is problem on follow up, she did not complete the follow up. Then after, I am giving her the drug.

Interviewer: why did you interrupt the follow up?

Response: they told me that she is fine. They stopped treatment, disconnected something from her nose (oxygen supply). When they interrupt the drug from her nose, she begins breathing fast. But when for test the disconnected the drug from her nose (oxygen), she become fine for one day then at the next day they said yeah! She fine now you can go home. They appointed me to come back after fifteen days. I did it. They said she is fine, it was the main doctor. She was fine at that time. But the she become sick after two years.

Interviewer: after her health condition was hampered, what hindered you from seeking medical attention?

Response: actually I have no money; I show them and told the condition, they told me to go to Hawassa or Addis Ababa, or other place to get treatment for her. They said, we have no treatment for her. To go to the mentioned place, there is capacity (money) problem. Then they gave me drug, she is taking simply drug. There was syrup, but she did not take it. There is drug every month,

Interviewer: hihihihihi… do you have other idea? Or question or else?

Response: no question.

Interviewer: thank you very much for your time and the information you shared with us.
