# Supplementary material for: Why are the implementation of and access to child developmental assessment within routine healthcare so difficult in Ethiopia? A descriptive qualitative study
Source: PLoS One. 2026 Aug 10;21(8):e0355686. doi: 10.1371/journal.pone.0355686 (PMC13456246; doi:10.1371/journal.pone.0355686)
Supplement: Supplementary file 2 [file pone.0355686.s002.zip › data/Transcripit 13.docx]

**Transcript 13**

**Interviewer:** my name is X; my friend’s name Is Y. We are from Arba minch university. We are health professionals. We are conducting research on child development, developmental delay. We are going to ask you some questions regarding child developmental delay assessment. It may take 30 up 40 minutes. We will be focusing on developmental delay, and normal child development. Thank you for your willingness to participate.
**Respondent:** do not ask me I don’t know many things.

**Interviewer:** we will ask you very easy questions and we can explain it for you. How old is this child?
**Respondent:** five years.

**Interviewer:** how many children do you have?
**Respondent:** three.

**Interviewer:** where did you give birth of this child?
**Respondent:** Arbaminch hospital.

**Interviewer:** how is your developmental follow up experience regarding this child? Where did he follow developmental follow up?
**Respondent:** I started the follow up at hospital and changed to here (Lante health center).

**Interviewer:** why did you transfer to Lante health center?
**Respondent:** I used to live in Arba minch and changed the area. I was in Arba minch.

**Interviewer:** what follow up did he make?
**Respondent:** he was admitted to warm room (NICU). He is lucky, he escaped many death. During labor he was harmed, there was prolonged labor, he was hurt and admitted to warm room.

**Interviewer:** what was the reason for his admission to warm room?
**Respondent:** during labor he became weak and unable to breathe correctly. It was rainy day, and midnight. No transportation to health facility, we waited for long time before reaching hospital.

**Interviewer:** how long did he stay in the warm room?
**Respondent:** fifteen days. Then after discharge he was also infected by cold (pneumonia) after 20 days and admitted for 23 days again.

**Interviewer:** what is cold disease? Was there coughing?
**Respondent:** it was due to washing by cold water, yes there was cough.

**Interviewer:** how is his vaccination status?
**Respondent:** he completed immunization, I have his certificate.

**Interviewer:** you told us that you were in hospital with this child; from the services they provided for you which service did you like more? Any disappointing service or you dislike?
**Respondent:** nothing disappointed me. It was good service I am happy.

**Interviewer:** think about the treatment and services at that time, what are the services provided for him?
**Respondent:** they put him in machine. From the machine they give him to me to breastfeed, and then he became breathless and cold body. Then they immediately took him to the machine and gave him warmer. Good help and he became fine.

**Interviewer:** what was the weight of the child at birth?
**Respondent:** I don’t know the weight.

**Interviewer:** what do you know about normal child development or abnormal child development?
**Respondent:** I don’t know. A child born fine grows normal.

**Interviewer:** For a child, there is time for sitting, time for crawling, time for walking, time for speaking… which we call normal development. When do you say a child is growing well?
**Respondent:** are you referring to my child? He is behind.

**Interviewer:** not only your child, generally what is normal child development and abnormal child development for you?
**Respondent:** when a child turns around by the time, crawling at eight month… walking supported by chair. Those children who immediately start walking—this is normal development.

**Interviewer:** how about abnormal development?
**Respondent:** sitting for long time, or remain at sitting position, or not turn around or not walking, start speaking while in sitting position, and order older brother or sister before standing up. While walking and not speaking is not normal. All the events must be synchronized.

**Interviewer:** what are the signs of developmental delay?
**Respondent:** all I mentioned before: not speaking, not walking, not crawling as per their time is sign of abnormal growth.

**Interviewer:** which type of developmental delay is common in this area?
**Respondent:** mostly standing problem.

**Interviewer:** how do people or you know that a child has developmental delay?
**Respondent:** in comparison with peer—equally or younger age child performs more than the delayed child.

**Interviewer:** where do people go when there is developmental delay? When this condition is identified?
**Respondent:** in one year, it can be identified. A child must stand up and walk by a year.

**Interviewer:** is developmental delay identified in one year?
**Respondent:** yes in one year.

**Interviewer:** is it not possible to identify before one year?
**Respondent:** yeah! It is not easily identified before a year.

**Interviewer:** how about after a year? About speaking? When?
**Respondent:** speaking might be genetic based… speaking has many reasons, I don’t know about speaking anymore.

**Interviewer:** do you have any information about the service regarding developmental delay assessment, growth monitoring, care and treatment?
**Respondent:** yes!

**Interviewer:** okay! You know the service?
**Respondent:** ohoo! Not me—it is health care providers, they know the service.

**Interviewer:** I am not talking about health care providers. Do you know the service?
**Respondent:** no I don’t know.

**Interviewer:** just the existence of the service… do you have information about the presence of the follow up?
**Respondent:** yes I know the presence of the follow up.

**Interviewer:** okay, fine. What is done for the child during follow up?
**Respondent:** they give advice to take care of children affected, and it depends on the level of problem.

**Interviewer:** is it education?
**Respondent:** yes.

**Interviewer:** what is the service for development assessment follow up? What do they see? Where is the service delivered—hospital, health center or health post?
**Respondent:** I guess it is provided in health post.

**Interviewer:** are you guessing?
**Respondent:** yes! It is delivered in health post. I hope it is delivered in health post.

**Interviewer:** who is in charge?
**Respondent:** the main body, the main responsible bodies.

**Interviewer:** who are they?
**Respondent:** I think it could be health extension workers.

**Interviewer:** if you know the service, say few things about who give the service? Where is it available?
**Respondent:** I did not use the service.

**Interviewer:** where did you make follow up for this child?
**Respondent:** I follow immunization here, I do not know other thing.

**Interviewer:** sure! Did this child get diagnosed for developmental delay or made any follow up about growth and development?
**Respondent:** many people ask and leave. Some say this child is affected, some say no problem he is fine. People come and go, no one has remedy.

**Interviewer:** did the child make any developmental assessment?
**Respondent:** not at all.

**Interviewer:** did people come by their own to give the opinion?
**Respondent:** yes.

**Interviewer:** who are they?
**Respondent:** they are health workers; I don’t know where they come from.

**Interviewer:** did you take this as problem? If you take it as a problem, did you try to go for medical attention?
**Respondent:** health professionals said, it is not a problem. Then how could I say it as a problem? Where should I go? I am not better than them.

**Interviewer:** you did not go anywhere to seek medical attention for this child?
**Respondent:** nowhere.

**Interviewer:** he is five years old, he didn’t speak?
**Respondent:** yes!

**Interviewer:** when did you identify the problem of this child?
**Respondent:** when he was two years old.

**Interviewer:** you didn’t identify before?
**Respondent:** yes! It was after a lot of prayer he stand up, but the mouth is not opened (not speaking).

**Interviewer:** what was the feeling? What do you feel?
**Respondent:** we think it was due to problem during birth.

**Interviewer:** is it major issue in the family?
**Respondent:** it is major issue. He do not know where to go, how to come back. He knows nothing. Do not identify good or bad.

**Interviewer:** did you seek any help?
**Respondent:** we did not get any help.

**Interviewer:** did you seek it? search for help?
**Respondent:** yes, he was registered.

**Interviewer:** where did you register?
**Respondent:** here, in kebele.

**Interviewer:** was it about aid for children with developmental delay?
**Respondent:** yes.

**Interviewer:** who triggered the registration—you or people who came?
**Respondent:** most people do not consider the problem a problem. I came to find some help. But there was no help.

**Interviewer:** what hindered you from searching for help?
**Respondent:** nothing hinder me.

**Interviewer:** okay! Why you didn’t make follow up?
**Respondent:** where is the service?

**Interviewer:** …you do not know the service?
**Respondent:** yes! I have no idea.

**Interviewer:** what if the service is available, are you ready to make follow up?
**Respondent:** yes. It is for me, for my child—why not?

**Interviewer:** …what is the problem in the community that makes families not follow developmental delay assessment?
**Respondent:** I don’t know.

**Interviewer:** is it only not being aware of the service?
**Respondent:** yes.

**Interviewer:** now we are telling you there is service. Are you ready to follow up?
**Respondent:** yes.

**Interviewer:** what can prevent you from seeking medical attention?
**Respondent:** nothing prevents me. I don’t know the service and where to go.

**Interviewer:** now that you know the service exists, what is your attitude to receive it?
**Respondent:** if you say go there, come here, I will go and come.

**Interviewer:** how do you feel about the availability of service?
**Respondent:** Yeah! I will make the follow up. It’s good for me.

**Interviewer:** regarding this child you don’t have any service or follow up from health facilities?
**Respondent:** yes nothing.

**Interviewer:** do you have any question comment or concern?
**Respondent:** I have no question. When the child becomes sick I take him to Red Cross. Otherwise I do nothing.

**Interviewer:** where is Red Cross?
**Respondent:** Dilfan Kebele.

**Interviewer:** have you been in Arba minch town?
**Respondent:** yes.

**Interviewer:** when did you come here?
**Respondent:** ten years.

**Interviewer:** therefore this child was born after you came to Chano?
**Respondent:** yes, but delivery attended at Arba minch hospital.

**Interviewer:** how did she recognize the problem?
**Interviewer:** she said they recognized the problem at age two—he was unable to speak.

**Interviewer:** where did she go first for help?
**Interviewer:** where did you go first when you recognized the problem?
**Respondent:** I go church to get help.

**Interviewer:** other place?
**Respondent:** I made an immunization follow up; no one recognized the problem. No one referred me. So I go to church. Prayer made him stand and walk.

**Interviewer:** why she did not go to hospital?
**Interviewer:** she said she was not aware of the service.

**Respondent:** health providers did not give me appointment to come back. During birth we were there; during immunization we came, but no one considered his problem.

**Interviewer:** you do not think help might be available in health facility?
**Respondent:** no expectation—no one considered his problem.

**Interviewer:** do you have any other ideas?
**Respondent:** no idea.

**Interviewer:** there is service given in health facilities; you need to make follow up…
**Respondent:** (child crying)

**Y:** does she go to other place for help?
**Interviewer:** she didn’t go anywhere. If no additional ideas we finish here. Thank you.
