# Supplementary material for: Why are the implementation of and access to child developmental assessment within routine healthcare so difficult in Ethiopia? A descriptive qualitative study
Source: PLoS One. 2026 Aug 10;21(8):e0355686. doi: 10.1371/journal.pone.0355686 (PMC13456246; doi:10.1371/journal.pone.0355686)
Supplement: Supplementary file 2 [file pone.0355686.s002.zip › data/Transcripit 14.docx]

# RECORD: 160830_2227T3

Interviewer: thank you for being willing to the interview. I introduced my name X and my coworker Y. We are health care workers and teachers from Arba Minch University, we came here for research purpose. You came to the health post for immunization purpose.

Respondent: yes.

Interviewer: what do you know about child development? Or growth?

Respondent: I am mother; child development knowledge is mandatory for us. We care, keep hygiene of the child.

Interviewer: it is not merely to mean. We are referring about the behavior they obtain at age of eight months, at age of one year, and the like. It is called developmental milestone in medical wording.

Respondent: yeah! At age of eight it crown, at age of one year start walking, stand up…

Interviewer: yes. We are talking about natural process, but not the care given by families. We need to be on one page. How old is this child?

Respondent: one year and three months.

Interviewer: how many children do you have?

Respondent: three children.

Interviewer: where did you attend birthing for this child?

Respondent: at Lante health center.

Interviewer: where do you attend health follow up?

Respondent: here in health post.

Interviewer: okay! Is he getting immunization?

Respondent: yes, he completed the immunization. He will finalize today. Today’s immunization is the final one.

Interviewer: how is your health follow up experience? Did you miss appointment? Low or good adherence?

Respondent: there are individuals who support the health extension workers. They watch our appointment date and call us. They go house to house to call us and tell the time and date of immunization.

Interviewer: you mean there are people who support health extension workers?

Respondent: if they give the vaccine house to house those individuals support them. Not only child immunization, the eye drug and deworming drug supply. You see many women with children—these are all called by those individuals.

Interviewer: when do you say the child is growing well? Or in good manner? To say a child is growing in good manner what are the signs? How do you know?

Respondent: growing well? I don’t understand your question?

Interviewer: to say there is problem on growth and development. As we had discussed before, a child should crown at age of eight months, stand up at age of one year, and the like.

Respondent: yeah! Very fast child can walk at age of eight months.

Interviewer: yeah? Not only walking, all other conditions.

Respondent: in the first month we expose the child to sunlight. Up to six months only breast milk. At this time children can sit. Up to eight months they start crowning. Up to ten months begin standing and walking. Up to two years they normally speak.

Interviewer: You mean this is normal development? There is no problem?

Respondent: no, there is problem, there might be disease. They become sick and this step will be interrupted.

Interviewer: what are the signs of child with problematic growth? How can it manifest?

Respondent: they cannot walk even by one year. Can’t crown at age of eight months. There is a woman who comes with me, her child is affected. She goes to hospital, tries all treatments, but no change.

Interviewer: how old is her child?

Respondent: one month behind my child (her child is one year and three months old). Did not crown, didn’t stand. She keeps him hugging. (You need much time? question from other individual, might be 20 minutes for Selam)

Interviewer: what are signs of problematic child development?

Respondent: the child crown at age appropriate to crown. Sick children do not crown while healthy children do. Children affected by disease can’t walk at their appropriate age.

Interviewer: what are common developmental problems? Is it not walking or not speaking or else problem?

Respondent: problem is common at early ages, before one year. To stand up it takes time. Most children are affected by this aspect. Diarrhea due to tooth eruption, and to widen abdomen. There are many problems up to one year of age.

Interviewer: up to five years. About speaking, about walking.

Respondent: after they start eating food, they withstand even when they become sick. From only breast milk to additional food (complementary feeding). The problem is common, but as they eat much food they become strong enough to withstand disease.

Interviewer: how do people in this vicinity identify a child with developmental delay or problematic development?

Respondent: it is by disease. They recurrently become sick.

Interviewer: becoming sick? What disease?

Respondent: it is different; there is pneumonia, tooth disease, malaria, typhoid, typhus, and the like. All the health problem of adult is problem of the children too. Children not affected by disease can show good development. On the other hand sick children lag back from growth. The sick child is not equal with the healthy child by development.

Interviewer: when do people notice them?

Respondent: at age of walking. In comparison with peer group. Children born equally should walk equally—unless there is problem.

Interviewer: this is to mean after one year. Do you know developmental assessment service available?

Respondent: do you mean family or else?

Interviewer: it is about small children just like immunization. There is developmental assessment service, developmental assessment is among the services.

Respondent: is this service available in health post?

Interviewer: yeah! Can be in health post or health center.

Respondent: yeah! yeah! They ask. Health extension workers follow house to house. If our children are affected we inform health extension workers and they advise us to go to health facility.

Interviewer: what service do you get when you come for developmental assessment?

Respondent: still now they did not do anything for us. This phenomenon has not been happened, we do not see it, and it’s new for us. Due to this reason they have nothing to do.

Interviewer: developmental assessment is for every child, not for a child with a developmental delay only. You know immunization is for every child—just like immunization, developmental assessment is for every child regardless of their health status.

Respondent: yes of course, health extension worker do everything.

Interviewer: okay. What activities do they do for developmental assessment?

Respondent: the health extension workers, what they do for us is: they come when immunization day arrives, they give drug house to house if the drug is to be given, they give us advice, they call us to health post and give us advice about child growth and feeding as per their age, what care for the mother at the time of birth and during pregnancy. Health extension workers give education for us.

Interviewer: this education is good education, but how do they assess child growth, whether appropriate or not?

Respondent: the health extension workers?

Interviewer: for example one year old child, two years old child, and children in months. What developmental signs do they observe or tell you?

Respondent: three-and-a-half-year-old child go to school, KG, then up to seven years, grade one.

Interviewer: like immunization, do you know the existence of developmental assessment as service in health facility?

Respondent: I don’t know. It did not happen yet. I haven’t seen it.

Interviewer: what is developmental assessment? How do you perceive it?

Respondent: I do not know it. It did not happen.

Interviewer: okay. How many children do you have?

Respondent: this one and his older sister and younger sister only two years old.

Interviewer: you said you do not know about developmental assessment. Therefore, they did not get developmental assessment?

Respondent: yeah! I do not know it. It did not happen to me. I haven’t received education about developmental assessment. If they taught me, I would tell you something but I have not learned it. I do not know it. (Background sound from kebele meeting). If it is about immunization, I know it and all my children got immunized.

Interviewer: you do not know about growth monitoring at all?

Respondent: I have not received education about it.

Interviewer: okay we are health care professionals, what do you think is developmental assessment?

Respondent: when I think about developmental follow up, it is all about feeding, keeping hygiene, and all the cares I offer for my children. I do not think other than this. (Background sound from kebele meeting)

Interviewer: if she knows about developmental assessment, why she did not make the follow up?

Respondent: she did not know about it.

Interviewer: did not learned about it.

Respondent: I did not learned about it.

Interviewer: she said she has no information about growth monitoring, about developmental assessment service. Therefore, she is not eligible for the following questions.

Interviewer: YES!

Interviewer: okay! There is a service for developmental assessment; there are developmental milestones at different age groups. It is known even though you do not know. It tells about walking, speaking, different activities. The service evaluates this natural process and labels the children normal or delayed in development. A child unable to attain that milestone is labeled as a child with developmental delay—walking, standing, crowning, speaking, and all characteristics. Thus, be informed that the service is available and try to use it. Do you have any question or concern?

Respondent: I have no question. (bird singing)

Interviewer: thank you for your time and interview you made. Thank you!
