# Supplementary material for: Why are the implementation of and access to child developmental assessment within routine healthcare so difficult in Ethiopia? A descriptive qualitative study
Source: PLoS One. 2026 Aug 10;21(8):e0355686. doi: 10.1371/journal.pone.0355686 (PMC13456246; doi:10.1371/journal.pone.0355686)
Supplement: Supplementary file 2 [file pone.0355686.s002.zip › data/Transcripit 15.docx]

**Transcript 15**

**Interviewer:** My name is X; her name is Y we are from Arba Minch University. Thank you for your willingness to participate in the interview. Now we come here to make in-depth interview regarding child development. Child development can be delayed which is abnormal and normal without any problem which is normal. We mean development how a child behaves or shows character at different age levels can at month four, eight, one year, two years and the like.
**Respondent:** yes I know.**Interviewer:** therefore, some children lag behind their age. We are to make interview regarding such condition. How old is she?
**Respondent:** for this child
**Interviewer:** yes, is there any problem her on development
**Respondent:** no problem she is fine.

**Interviewer:** do you have with hampered development
**Respondent:** no. I haven’t

**Interviewer:** how old is she?
**Respondent:** in the coming December she will two.

**Interviewer:** how many children do you have?
**Respondent:** three

**Interviewer:** where was delivery attended for her?
**Respondent:** here
**Interviewer:** here in health post?
**Respondent:** yes in Arba Minch hospital.

**Interviewer:** is she receiving follow up, and immunizations?
**Respondent:** yes she started immunization at fifteen days

**Interviewer:** is there other health follow up for her? Regarding growth?
**Respondent:** thanks to God she is fine. She is growing well.

**Interviewer:** only immunization no other follows up?
**Respondent:** yes. You other follow up is if there is a problem.

**Interviewer:** when you go to health facility for immunization, how is their service?
**Respondent:** when I go to health facility they make a diagnosis and give injection.

**Interviewer:** how was that service was it satisfying or annoying.
**Respondent:** it was very satisfying, very good. There is diagnosis, they blood sample, stool, they watch for fever, they see everything, and then if there is disease they give drug.

**Interviewer:** when did you say a child is developing well?
**Respondent:** with the help of God, if a child grow without disease.

**Interviewer:** it is to mean the characteristics they show at different ages like four-month, eight month…..
**Respondent:** up to four months only breast milk then after six month some few additional foods, at month nine immunization is complete, up eleven month the child completes all the vaccination.

**Interviewer:** you know by month five a child turns around, at month eight crown……. we are talking about this kind of changes on children.
**Respondent:** on month four it try to sit down, on six-month crown and begin to stand up supported by chair, at eight month a child is becoming stronger and stronger.

**Interviewer:** you said a child can walk by month six. Can a child stand up and walk by month six?
**Respondent:** it crowns

**Interviewer:** with this regard what is it mean to you when a child is delayed in development?
**Respondent:** yeah! Some children affected, can’t walk alone, affected mean if incomplete body parts (disabled). Can be on leg, on hand, if something happens.

**Interviewer:** without visible problem on their hands and legs, some children shows delay in development. We are focusing on such conditions. With this regard what are the signs which shows the developmental delay of a child.
**Respondent:** this happens when there was something happened during birth.

**Interviewer:** what sign can you observe?
**Respondent:** can’t walk

**Interviewer:** in what age?
**Respondent:** starting from eight month, can’t show changes. Unable to stand up from sitting. This is the sign. And can’t move here and there.

**Interviewer:** which type of developmental delay happen in this area? Is it unable to speak or unable to walk?
**Respondent:** with the help of God this kind of problem did not happened in our area. No problem happened in our community. A child not immunized suffer a lot, unimmunized child suffers, when a child receive immunization and a drug given via mouth, this is very good.

**Interviewer:** how do people know if the child has developmental delay?
Just like that child it happens as a chance, it might happen at birth or after birth. It is God’s right,

**Interviewer:** when do people identify developmental problem happened to a child? In what age?
**Respondent:** even at time of birth, mother can identify a problem. For example I know everything about my child. If there is a problem I can identify it.

**Interviewer:** you immediately at birth developmental problem can be identified you say?
**Respondent:** yes. Mother can identify

**Interviewer:** how about other people, how can they identify?
**Respondent:** other people can identify if I told them. They ask, what happened to this child? Then a mother tells them about the child’s condition.

**Interviewer:** you say people can’t recognize by sign?
**Respondent:** other people simply say what happened to this child? Ask the mother, it the mother who knows the child’s condition and tell for the people. Or health care providers in health center or hospital can show direction where to go for help. They order to go somewhere for help and treatment. But other people simply say take the child to hospital or health center. They do not know what happened to the child.

**Interviewer:** you know development or growth assessment service available in health setting?
**Respondent:** yes

**Interviewer:** what did they do for development assessment?
**Respondent:** they declare immunization campaign, if someone did not come to the immunization they ask the mother why you were absent for immunization day and follow up.

**Interviewer:** it is not immunization, I am referring to developmental assessment., whether the child lag behind from his/her equal age?
**Respondent:** yes they ask it.

**Interviewer:** if yes, what did they do for the assessment?
**Respondent:** if the child lags behind, health extension workers make follow up and give food (alimi migib). For sick children, for children with emaciated body. This might happen due to lack of milk in the mothers’ breast. If there is feeding problem in the household, the mothers’ breast has no milk. They give (almi migib) until the child’s health return to normal. Then children regain their health and start growing well.

**Interviewer:** previously we have discussed that four month age child turn around, eight months age child crown, and at age of one year the child stand up and start walking. I am talking about this condition. Is there any follow up regarding this condition?
**Respondent:** yes!

**Interviewer:** where did the follow up done?
**Respondent:** during immunization day they ask us. They ask about breast feeding, and about many other things.

**Interviewer:** when and how often the ask this?
**Respondent:** at immunization day. At meanwhile they come to home and ask is your child good? And growing well?

**Interviewer:** yeah! Fine other where saying that it is only immunization no other service in health facilities but you are addressing the existence of development assessment service in health post and health centers. From where did you heard about this service?
**Respondent:** I have seen as they were working. The health post is nearby. They ask different issues, give bed net, this is what I have seen, they ask me how your child is? They follow cleanliness of latrine, at that time if there is some draw back on children they comment to modify the condition.

**Interviewer:** is this child received developmental assessment follow up?
**Respondent:** yes! They ask?

**Interviewer:** what is done for her?
**Respondent:** just they see her as she is growing well.

**Interviewer:** what did they do?
**Respondent:** they ask as how is she developing?

**Interviewer:** how did they evaluate whether she is behind her age or not? How old she?
**Respondent:** in the coming December she will two.

**Interviewer:** how did you recognized whether the health care providers were assessing her development status? Is she performing what is expected from one years old child by age one and two too?
**Respondent:** as she is growing well they did not do that, but if she feels sick I come to them. They treat her, make follow up. They give her everything needed. They give drugs.

**Interviewer:** to make health follow for the child, was there any fear about delay in development or not?
**Respondent:** I have no fear. Everything is passed; I made immunization and everything, now she can walk with the help of God.

**Interviewer:** how was the service you received? Was it satisfying or annoying?
**Respondent:** no, it’s very good. They serve in good manner.

**Interviewer:** which service satisfied you more?
**Respondent:** with help of God, the immunization they provided for my child was good. Bed net, bed net helped me much. They vaccinate me, the mother.

**Interviewer:** which service you disliked?
**Respondent:** nothing.

**Interviewer:** what did health care providers ask you?
**Respondent:** told me to take vaccination, to make movement while I was pregnant. They measure blood (which is to mean BP). Minimize salt, do not miss what we told you, and this is good service and they identify problems.

**Interviewer:** is there anything you worried about while they give the service?
**Respondent:** nothing.

**Interviewer:** what is her developmental problem?
**Respondent:** she has no problem.

**Interviewer:** you have told us that there is developmental assessment service here in health center and health post. What conditions facilitate them to use developmental assessment service? And what conditions hinder them from using this service? You know early identification helps to seek early medical attention and the condition reversibility increases. If lately recognized the condition might not be reversible.
**Respondent:** it is family problem

**Interviewer:** What is family problem?
**Respondent:** if I bring this child here, they may order me to go other place. This condition is associated with lack of many. There is also a family who can urgently take to the place referred (ordered). Most people are in lack of many, therefore if they told to go urgently, there will be problem of money. Those people waiting for money to come or search for money for long time, to go the referred health facility. People having no problem with money can go early get advantaged. It is all poverty, the leaving difference.

**Interviewer:** this is what hinders form seeking medical care, what about facilitating condition or factors?
**Respondent:** it is father and mother. They can go anywhere for their child, get loan for their child. They come here early if the condition is easily managed here they will go back to home; they get treatment, injection and education. Poor people can go to treatment after one or two week after getting loan or selling his/her asset.

**Interviewer:** the hindering condition on the health facility side?
**Respondent:** they say take to higher level, they do not prevent. They treat free of charge. There is health insurance again. There is nothing on the health facility side.

**Interviewer:** do you mean community based health insurance?
**Respondent:** health insurance most people are insured.

**Interviewer:** how about on the side of health care providers? It can be barriers or facilitators
**Respondent:** it is good, they don’t hate us.

**Interviewer:** what is other conditions that hinder people from their own side.
**Respondent:** it all about lack of money. Lack of food for children, minimum choice of food due to lack of money. He is unable to buy food. For example this feeds everything it is my capacity to provide all the necessary types of food.

**Interviewer:** what conditions do you know that helps people to use developmental assessment? What do you mean? It is from you knowledge or opinion, not what happened to your child.
**Respondent:** actually if there is serious problem on his/her, it is economic problem. If the health care providers assess and refer to other place early people will see for alternatives, loan or selling asset. God bless them, there is ambulance.

**Interviewer:** facilitating conditions on family side, community side, healthcare providers side, etc.
**Respondent:** on family side if my child grow well, I will not come for developmental assessment. If the child become sick and challenge me, I will come for assessment. If treated and become well, I go to my home and keep quite. If the child become sick I come back to health facility …they give injection then go to home. They give us appointment, change drugs. On family side if the child fine no need to make follow up for developmental assessment. With the help of GOD in my community there is no problem children grow fine and well. Health post workers are good.

**Interviewer:** what do you think when we speak about child developmental assessment. Is it good or bad?
**Respondent:** it is good

**Interviewer:** how good?
**Respondent:** if the child is fine and no illness that is good for me.

**Interviewer:** I am talking about the service we have discussed about. About early detection of problem and solve it. Is the existence of developmental assessment service good, if good how good?
**Respondent:** regarding this question, if the child sick and I come to health facility after treatment the child become well and good. That is good for me. Healthy growth is good. They grow up to one year, eight month, and by six month we begin some few foods.

**Interviewer:** which part of the existing service, you think needs improvement?
**Respondent:** in the future?

**Interviewer:** yeah! In the future
**Respondent:** we need diagnosis, treatment, vaccination, and if the child is not well, and malnourished we wish to receive foods, we wish to receive bed nets, and all the needed service for children.

**Interviewer:** do you have any question?
**Respondent:** no

**Selam:** you have seen a child with some health problem, when did the families come to health facility for child having that kind of condition? In how sever condition they seek medical attention.
**Respondent:** if the child fails to walk. They need to go health facility, it needs diagnosis. It needs follow up; if the problem is not managed here they need to refer to the higher level.

**Interviewer:** when did people come to health facility for medical attention?
**Respondent:** at birth, or three days not possible to identify. Four months, when fail to sit-down. Fail to gain weight, at six month begin some few foods and start walking supported. If the child fail to do so. They come to health facility

**Interviewer:** is this habit common practice?
**Respondent:** no, with help of God this condition is not common here. Thanks to God this condition is not seen in our community. I don’t know about the condition. Children grow well with the help of God.

**Interviewer:** I mean to say did people want to seek medical attention as soon as possible, if developmental delay problem happens? I am not saying developmental delay problem is common condition.
**Respondent:** there is no such condition. But people go to medical care as soon as possible. I know people bring to health facility soon.

**Interviewer:** if the child fail to walk by age of one year or fail to crown by the age of eight month? Did they bring to health facility?
**Respondent:** yes.

**Y:** you no one ignore to come to health facility?
**Respondent:** no one

**Interviewer:** you say there is no one waiting for the child’s condition be self-limiting. Other people said people wait for the child to walk by herself or himself without medical intervention for more than a year. What do you say on this issue?
**Respondent:** no wait in this way. I don’t know. People bring to health facility, the community members ask did you showed for health care providers, and did you go to hospital?

**Interviewer:** okay, Y do have something to add?
**Y:** no more

**Interviewer:** thank you for your information and time you spent with us. We finished our interview here.
