# Supplementary material for: Why are the implementation of and access to child developmental assessment within routine healthcare so difficult in Ethiopia? A descriptive qualitative study
Source: PLoS One. 2026 Aug 10;21(8):e0355686. doi: 10.1371/journal.pone.0355686 (PMC13456246; doi:10.1371/journal.pone.0355686)
Supplement: Supplementary file 2 [file pone.0355686.s002.zip › data/Transcripit 16 .docx]

**Transcript 16**

Thank you again for your willingness to participate in our study. You were selected for this study because of your connection to the subject. At the same time, I would like to remind you that there is no such thing as a wrong answer.

Interviewer: -Can we continue?

Respondent

Interviewer: - Tell me a little about yourself and your family

Respondent: - Certainly, we can continue

Interviewer: - How old is this child

Respondent: - she is one year

Interviewer:-How many children do you have?

Respondent:- I have six children this girl is the sixth one

Interviewer:-Where did you give birth to this child?

Respondent: _ I gave birth to her child at the hospital after undergoing a cesarean section due to a failed attempt at instrumental delivery. Unfortunately, i encountered a complication of fistula. The condition of fistula was challenging and was referred to Addis Abeba and treated there.

Interviewer:-Where do you get child health services for this child?

Respondent:- I have been utilizing child health services for my child at the local health post. The health post has been the primary source of healthcare for my child, specifically for their child health needs. The health post has provided a range of services, including routine check-ups, immunizations, and healthcare advice. I have found the health post to be accessible, conveniently located within the community. The health post also providing education and guidance on various aspects of childcare, nutrition, and hygiene.

Interviewer: Has this child been vaccinated?

Respondent: Definitely she is vaccinated

Interviewer: - How many times she talked the vaccine?

Respondent:-She has been fully vaccinated according to the recommended schedule. She has received all the necessary vaccinations required for age.

Interviewer:-could you please tell me about your general experience with your child at the health facility?

Respondent: - I visited the health post, I sought vaccination services according to the schedule and when my daughter required medical attention for an illness, she did not receive the examination she needed. The health professionals did not conduct any assessment or provide treatment for her condition, specifically related to her enlarged head

Interviewer;-When do you say that a child is not developing healthy

Respondent: - When observe a child's development, it is important to observe their ability of sitting, walking, and there general health condition like frequent sickness ,weakness, and emaciation to be sure they are progressing as expected. For example, my child is not able to sit or walk yet, it can be a cause for concern regarding her health and development.

Interviewer: - When do you say that a child is developing healthy?

Respondent: - I believe a child is healthy when they are able to walk on their own two feet. Unfortunately, my daughter is unable to walk and sit due to the size of her head.

Interviewer: _What the signs of unhealthy growth are of chilled?

Respondent:- Signs of unhealthy growth in a child include inability to sit or walk, frequent crying, weakness, frequent sickness, vomiting, and fever

Interviewer:-What are the most common developmental problems that children face?

Respondent:-the most common developmental challenges that children encounter is the inability to sit and walk.

Interviewer:-How do you know that a child in your area does not have healthy development?

Respondent:- I can't speak for other children, but in my situation, my daughter cannot sit and walk and in addition fathers in our community do not show much concern for their children's well-being, including issues related to walking, proper childcare, health, nutrition, and other essential matters.

Interviewer:- How long does the problem usually occur when they find out they have a developmental problem?

Respondent:-I am aware that she experiences an issue due to the size of her head. However, I did not anticipate that it would be as severe as it is now, hindering her ability to sit and walk.

Interviewer:- Have you ever heard of Child Development Assessment?

Respondent:-I haven't heard of Child Development Assessment. What is it?

Interviewer: - what do you think?

Respondent:-i did no hear about it before and i don’t know anything about Child Development Assessment

Interviewer: - Has your child had any developmental tests before?

Respondent:-No, my child has not had any developmental tests before.

Interviewer: What was the reason for not getting a developmental diagnosis?

Respondent:-I did not have the information about Child Development Assessment and I accept I as Gods gift

Interviewer: - When did you first notice that your child had a developmental difference or delay?

Respondent:- As mentioned previously, I understand that there were some issues after her birth due to her large head. However, by the time she turned six months old, she was not able to sit up. As of now, she is still unable to walk.

Interviewer:-How did you feel about it?

Respondent:-when I know that my child has a developmental delay i experience a range of emotions, including sadness, confusion, fear, guilt, and frustration. i feel overwhelmed by the challenges that lie ahead and worried about how i will be able to support my child's needs, especially given the financial constraints i faces. Additionally, i feel isolated and alone in navigating the complexities of managing my child's developmental delay, because I lack access to resources and support services.

Interviewer: - Are you concerned about the development delay? Why are you concerned?

Respondent: - Yes I am concerned about the developmental delay of my child may face numerous challenges. I have limited access to resources such as healthcare, developmental assessments, and early intervention programs due to financial constraints. Additionally, i experience added stress and anxiety due to the inability to afford specialized care for my child

Interviewer-_ Did you need help?

Respondent: - I didn't require assistance from anyone; I am patiently awaiting guidance from my God, and I have a plan to visit *yohanis Xebele*
