# Supplementary material for: Why are the implementation of and access to child developmental assessment within routine healthcare so difficult in Ethiopia? A descriptive qualitative study
Source: PLoS One. 2026 Aug 10;21(8):e0355686. doi: 10.1371/journal.pone.0355686 (PMC13456246; doi:10.1371/journal.pone.0355686)
Supplement: Supplementary file 2 [file pone.0355686.s002.zip › data/Transcripit 17.docx]

**Transcript 17**

Thank you once more for agreeing to take part in our study. You were chosen to participate in this research due to your relevant background. I also want to emphasize that there are no incorrect responses in this study.

Interviewer:-Can we continue?

Respondent: Yes, we can definitely continue

Interviewer:-Tell me a little about yourself and your family

Interviewer How old is this child?

Respondent;- The child is 5 years old.

Interviewer: How many children do you have?

Respondent; I have three children.

Interviewer:Where did you give birth to this child?

Respondent:- I gave birth to this child in the comfort of my own home. Although it wasn't planned, everything went smoothly

Interviewer:- Where do you get child health services for this child?

Respondent:_ I take my child to the health post in my community for all his health services. They have provided check-ups, vaccinations, and any medical care needs.

Interviewer Has this child been vaccinated? If yes, how many times?

Respondent:- Yes, this child has been vaccinated completely.

Interviewer Could you please tell me about your general experience with your child at the health facility?

Respondent:- I frequently visit the health facility for my child's regular illness. The health professionals there provide me with medication and a supplementary diet. They also educate me on child care, including hygiene practices and feeding.

Interviewer. When do you say a child has healthy development?

Respondent:- A child experiences healthy development when raised in good conditions, free from illness and stress, without creating stress and discomfort on the mother and A child is capable of walking and talking

Interviewer. When do you say that a child has unhealthy development?

Respondent: - A child's healthy development is hindered when they experience frequent illness, have difficulty walking and speaking, lack appetite, and do not engage in play with friends.

Interviewer:-What are the signs of unhealthy child has development.

Respondent: - The sign of unhealthy child development include difficulty with eating, frequent vomiting, recurring illnesses, inability to walk, and inability to tolk.

Interviewer:- How do you know that a child in your area does not have healthy development?

Respondent:- We are know that a child in your area is experiencing difficulties in their development, and we believe that their challenges with walking and speaking may be attributed to a lack of proper care and insufficient nutrition.

Interviewer: - Have you ever heard of Child Development Assessment?

Respondent: - Yes, I have heard of Child Development Assessment

Interviewer: - What is developmental assessment?

Respondent:-It is a health professional's observation and request regarding the care, nutrition, and cleanliness of our child.

Interviewer: - Where is the service of Child Development Assessment provided?

Respondent: - The service offered at the hospital

Interviewer: - Who provided the service?

Respondent: - The Health professionals

Interviewer:-How frequently does your child receive the service?

Respondent:-Whenever she falls ill.

Interviewer: - From where did you get the information on Child Development Assessment?

Respondent: - I obtained the information from other women.

Interviewer: Has your child undergone any developmental assessments previously?

Respondent: No, she frequently visits the health post for sickness but has not had a developmental assessment.

Interviewer:-Why didn't you undergo a developmental assessment for your child?

Respondent:- I was unaware of the need for a developmental assessment before

Interviewer: - When did you first observe signs of developmental delay in your child?

Respondent:-I became aware of my daughter's developmental delay when she started experiencing frequent illnesses.

Interviewer: - How did you feel about you child developmental delay?

Respondent: - I am feeling stressed

Interviewer:-Do you have any concern regarding the development delay?

Respondent: -Indeed, I do.

Interviewer:-Why are you concerned regarding the development delay?

Respondent:-As my daughter is unable to communicate and interact with other children, I am unable to fulfill my own work responsibilities to provide my family.

Interviewer:-Did you need help for your child?

Respondent: - Not at all

Interviewer: -Why didn't you seek help for your child?

Respondent: - I am ashamed from seeking help due to feelings of shame stemming from our economic difficulties.

Interviewer:-Were there challenge in asking for help ?

Respondent:-Just embarrassment.

Interviewer: Have any other personal factors impacted your experience? (Culture, income, environment)

Respondent: The only issue is related to my finances.

Interviewer:-What are barriers to developmental assessment of a child in your opinion, to get a developmental assessment service for the child.

Respondent:- In my view, the obstacles to conducting developmental assessments include financial challenges and absence of the services at the health post in our community.

Interviewer:-What are the obstacles of developmental assessment of a child on your side?

Respondent: - I lack the financial resources it is the only challenge for me

Interviewer: - What are the barriers in society?

Respondent: No issues related to developmental assessment of a child in the society

Interviewer:-What are the obstacles in a health facility?

Respondent: - Is not available at health post

Interviewer: What are challenges of child developmental assessment at the health facility?

Respondent: There is a lack of availability at the health post.

Interviewer:- What are the other barriers to access to developmental assessment services?

Respondent:- My financial situation is a the only obstacle.

Interviewer:-what is something that motivates you to get developmental assessment services for you child?

Respondent: - I found great motivation when my daughter's health deteriorated and required extensive medical attention unless I stay at home.

Interviewer:-What is motivates you to have a developmental assessment from the community? Respondent I appreciate the community's moral support in helping me achieve developmental assessments.

Interviewer: - What encourages you to seek a developmental assessment from a healthcare facility?

Respondent: - There is no incentive from the local health post to undergo further assessment at a higher healthcare institution.

Interviewer: - What motivates you to seek developmental assessment by health professionals? Respondent:-Same health professionals tell me about the importance to achieve development assessment frequently

Interviewer:-What are other motivational factors?

Respondent: - At church they pray for me and let me to achieve development assessment

.Interviewer:- In what way do you think the current service should be improved?

Respondent: - I don’t know the government	knows what to o and how to improve

Thank you
