# Supplementary material for: Why are the implementation of and access to child developmental assessment within routine healthcare so difficult in Ethiopia? A descriptive qualitative study
Source: PLoS One. 2026 Aug 10;21(8):e0355686. doi: 10.1371/journal.pone.0355686 (PMC13456246; doi:10.1371/journal.pone.0355686)
Supplement: Supplementary file 2 [file pone.0355686.s002.zip › data/Transcripit 18.docx]

Transcript 18

Interview: Tell me your daily routine work in pediatric care

Response: Thank you for considering me for this interview. My daily routine starts from the morning session which we hear from interns, then I go to the ward for round. Usually after round if it’s my turn to work at the referral clinic and look at different cases.

Interview: When do you say a child has a healthy development? Why?

Response: We say a child has healthy development when he can do the expected activities for each developmental aspect at the specific age.

Interview: When do you say a child has unhealthy development? Why?

Response: Unhealthy development is the exact opposite of health child development in which when a child did not grow or do the thing, he/she is expected to do at that specific age.

Interview: What are the signs of unhealthy development?

Response: The signs of unhealthy development are related with the domains of development. children having problem for each domain present with different signs. For example, if the child had problem with fine domain, he/she presents with floppy hands to hold on to materials. Whereas those children who had gross motor problem might not move their body.

Interview: What are the most developmental related health problems a child encounter?

Response: In our health facility the commonest cases I encountered in the referral clinics are children who did not start communication with the family or their peers. Th parents visit the pediatric clinics always complaining that their child has no started communicating as children born at the same time as their child. In addition, in the pediatric wards I usually encounter children whose body is not moving. They come when they got some complication.

Interview: How do you know if someone has developmental problem in your area (in this setting?

Response: we donot have any means of tracking those children but usually they get noticed in the community by parents when compared with their peers.

Interview: At what stage of the problem do you usually know the child has developmental problem?

Response: This is a nice question usually we found children with such problem while presenting with other medical conditions. Parents did not bring the child as early as possible. This might be for a lot of reason. First it might be due to the parents donot have knowledge about the condition and they usually took it as a natural process that the child will start talking or moving one day. This is usually a common way of think by most of the parents. Second, parents keep their child at home fearing the community since this kind of child condition is considered as a family curse by the community.

Interview: What services do you do to children to know their developmental status, currently?

Response: We do not offer any special service in the diagnosis and treatment of developmental in our setup. We use our clinical knowledge to identify the problem. Since the nature of the problem is wide and it needs a variety of diagnosis the condition and we donot have such advanced material we only use our clinical knowledge to do this.

Interview: How do you provide developmental assessment either in the community or health facility?

Response: We donot engage in the community-based activities but in the health facilities as I told use earlier, we do the assessment with our clinical knowledge. We donot have any materials to do the assessment, with the clinical diagnosis we refer to other health facilities in the country.

Interview: In your observation do you think the service you are providing timely, adequate? Why? HOW?

Response: I could say with 100% sureness, the service we are providing is not timely and adequate. I said because we are working in a setup with no material to investigate the condition. Currently the health care system is advanced with the use of technologies but we are here with nothing. Starting from the simplest screening tools, materials for assessment which could be used for observing the child while playing. In addition, for treatment we donot have any center with materials.

Interview: What make it the service you are providing different?

Response: Nothing I did not provide different service for the reasons I told you earlier.

Interview: What do you do when you know when a child has developmental problem?

Response: I refer to the higher hospitals.

Interview: How do you follow the child?

Response: If the condition is not complicated, we inform the mother or the care givers to do some activities related to the condition diagnosis. Then we give the caregivers an appointment to see the progress. However, they donot come back for follow up.

Interview: What proportion of children do you think will come to a health facility receive developmental assessment?

Response: To be honest I donot know the exact number but I could say a very few children.

Interview: Think about a child who received developmental assessment service, What does your interaction look like;

Response: I remember one child who come to the clinic because the mother complains the child was not moving her legs after a while and she was a 2 years old baby. So I start to do the assessment in examination bed in the clinic an it took me around 20 minutes. The only material I had at the time was hamer to check for the movement for all the moveable body parts. However, I want to check so many things but there are no such materials that could be used to examine the child by observation while playing with it but I could not do that because we donot have any.

Interview: Think about a child who did not receive developmental assessment service

What is the most common reason you have for not doing the developmental assessment? Why?

Response: This was the very heart-breaking story that I would remember. There was a child who come with complicated heart disease at emergency. After stabilizing the child, we notice the child had other health condition one was she had developmental problems. Asking the mother what is going on she mentioned with tears that she kept the kid at home and very few individuals now about her status. She was afraid that they would isolate her from the community. However, coming to my condition at the time the child need a very detail investigation and help with someone with

Interview: From your observation: What are the factors that could be barrier to do developmental assessment?

Response: I would start from the higher-level related barriers. So, from my observation the government specifically the ministry of health did not give an emphasis for the condition as that of other child health problems. In the health system different programs and service haven implemented for child health problems for example for immunization, the government created different strategies, initiatives to tackle the problem and to deliver the service. But in case of the developmental problems, I did not see such kind of dedication from the government. In addition, different stakeholders or non-governmental organization has not been facilitating to collaborate and work in this specific area.

In the health facility the competing need of different health problems and the very limited resource it makes it difficult to provide the service in terms of increasing the accessibility of the service by providing different material for assessment. In addition, the health care provider has no good knowledge to provide the service, treatment etc. Training regarding developmental assessment has not been given.

Interview: What is the most important factor to do developmental assessment?

Response: I believe all the above-mentioned factors are important but the government focus would be very important since all the other structures work basing it.

Interview: From your observation: What is the factor at that encourage to do developmental assessment?

Response: Unfortunately, I did not encounter any structural or policy related factor that encourages health care provider to do developmental assessment.

Interview: In your observation what do parents do when they know the child has developmental problem?

Response: This an important issue to discuss as parents play a very crucial and initial identification by noticing changes in the child by comparing with the peers. However, we encounter problem with parents as they consider the problem as spiritual problem and took their child to traditional healers and the holy water. This works as well for other health problem. They usually come to the health facility after the condition is complicated with other health problem. Most of the time after the condition gets worse.

Interview: In your opinion what is the attitude of parents towards developmental assessment? Why?

Response: Since we did not provide or deliver developmental assessment service in an organize way its very difficult to tell about the parents attitude but from the few cases I remember they were very worried about being referred to other facilities due to the financial struggles they have.

Interview: Who is the most important family or community member in seeking developmental services? how?

Response: I would say the mothers are the one to bring the child to the health facility most of the time.

Interview: thank you
