# Supplementary material for: Why are the implementation of and access to child developmental assessment within routine healthcare so difficult in Ethiopia? A descriptive qualitative study
Source: PLoS One. 2026 Aug 10;21(8):e0355686. doi: 10.1371/journal.pone.0355686 (PMC13456246; doi:10.1371/journal.pone.0355686)
Supplement: Supplementary file 2 [file pone.0355686.s002.zip › data/Transcripit 19.docx]

**Transcript 19**

Interviewer: Are you willing to participate and have your voice recorded?

Respondent: As long as the information and my voice are important, yes, I am willing.

Interviewer: As an introduction, tell me about the routine activities in the under-five OPD.

Respondent: In the under-five clinic, there is a triage room. In the triage room, they separate emergency cases from non-urgent cases. They send the non-urgent cases to the under-five OPD. These are cases like infectious diseases or other illnesses, and we treat them here.

Interviewer: What are the services you provide for developmental monitoring?

Respondent: Regarding child developmental monitoring, we assess the child's weight, height, and MUAC. To monitor these things, there is a separate room, which we call the triage room. After these measurements are taken, the children come to us. If we are suspicious of the measurements, we do a reassessment of the height and weight to check for malnutrition-related problems. We also give counseling to the mother on how to follow the child's progress and on homemade food preparation. We counsel them on the types of food they can use that are available in the household, and we give advice on how to prepare a balanced diet for the child at home. We do not do anything else other than this regarding child development monitoring.

Interviewer: Can you please tell me the difference between growth and development?

Respondent: Growth means that after the child is born, the height and weight are checked for their age, and if they are in line with the age in the presence of appropriate care, we call it growth. Whereas development means… it can be an increase in size or… what can I say? Development is a general thing.

Interviewer: What do you mean by "it's a general thing"?

Respondent: What can I say? It is something related with thinking or relating with parents. I don't know.

Interviewer: When do you say a child has healthy development?

Respondent: I say a child has healthy development first by looking at what age the child reaches a certain stage. For example, a three-year-old child has things he is expected to do. But if he is doing the activities of a one-year-old, like crawling at that age, I would say he is not well developed. I would say this child is delayed for his age.
For example, a six-month-old child—look at this thing *(he was showing a material in the room)*—if he has healthy development, his eyes would move around with it or he would follow the material. If the child is not happy, cannot respond to the environment, or becomes aggressive while looking at things, I would call this child not well developed. In addition, if the child is not satisfied with the interaction with the mother, I would call this a not well-developed child.

Interviewer: What are the most common development-related health problems a child encounters in this area?

Respondent: Most of the time in this area, we are serving a majority of the rural community. We do not often find children with a clear developmental problem. However, there are children who are considered an extra child and are ignored, growing up carelessly. In relation to nutrition, we find children who have had inappropriate development. For example, a five-year-old child in urban areas could enter KG school, but here they may not.

Interviewer: When they come, what kind of developmental problems do you observe?

Respondent: In my observation regarding developmental problems… *(pauses)* what can I say… if I say motor, the children seem normal, and if I say mental, they seem normal. But most of the time, they look like they are damaged in some aspect. What can I say… these children did not get what is necessary for them to grow. You can notice something different physically.

Interviewer: Tell me about it.

Respondent: For example, as a professional, you can notice children who are physically unhappy or wasted. The condition could be difficult to diagnose—it's hard to say if it's a nutritional problem or something else. You can also observe things related to their hygiene.

Respondent: Usually, parents bring the child to the health institution after they are already damaged. They do not bring the child early. They stay at home considering the child as healthy. They mention so many reasons; one is an economical problem. Second, the mother or the family member only notices the child is going through something after it gets complicated—they don't have the knowledge. Or they notice when they compare the child with his peers. But usually, it is an economical problem.

Interviewer: How are you doing developmental assessment currently?

Respondent: I have tried to mention earlier. If a child comes for care, vital information like height, weight, and temperature is taken in the triage room. Secondly, the complaint of the mother is our major source of information, and 75% of our work is based on that. In addition to the history, the physical examination I perform gives me more information. We assess subjectively and objectively, and then add lab investigation. If the cases are beyond us, we consult other colleagues.

Interviewer: So tell me about the objective assessments you do.

Respondent: Objective assessment first is a stethoscope, a spatula for tonsillitis, and other materials.

Interviewer: What about for developmental assessment?

Respondent: We use height and weight scales, and the growth monitoring chart.

Interviewer: This is for growth assessment. Do you have anything for developmental assessment?

Respondent: That is the thing—we don't have any materials for that.

Interviewer: Do you think the service you are providing currently is timely and adequate?

Respondent: Maybe as I have defined development in terms of motor, gross, or other domains, we need materials to do the assessment. For other things we can use what we have, but for these, it needs special material. It's very difficult to do. Especially because a child's nature is complicated in that they cannot communicate what they are feeling. We hear the necessary information from the mother. For example, for a child having epilepsy, we get all the information from the mother.
In addition, the setup is not fully functioning in terms of the organizational structure, the time to investigate, and the instruments required. For other cases, it's easy since we only use an interview and lab investigation to find out the problem. Those activities that need further instruments are not suitable for our setup.
I could say, professionally, I can give good service but it is not sufficient. However, since this is a primary hospital, we are providing the expected level of service.

Interviewer: What would you do if you found out a child had a developmental problem?

Respondent: I could not decide whether a child had a developmental problem on my own. We consult with other colleagues. Then we tell the mother accordingly. I could not decide by myself.

Interviewer: Why don't you decide?

Respondent: The reason is, it's multidimensional and I could not identify what it is with the current knowledge and materials in the facility. It needs further investigation. The only thing I can do is counsel the mother.

Interviewer: How many of the children get a developmental assessment?

Respondent: In the first place, the reason they come for care decides that. Most of the time, they do not come complaining of developmental delay. They usually come with some kind of illness. Then we look into children who are ill, and if we suspect the child's age and their activities do not match, we suspect a developmental problem. We do not do a developmental assessment for all children. Most of the time, they come with infectious diseases.

Interviewer: Have you faced any child with developmental delay?

Respondent: No, I have not experienced such a case.

Interviewer: What are the barriers to providing developmental assessment?

Respondent: Most of the time, the facility focuses on giving curative services. It does not focus on providing preventive activities. Also, most of the healthcare providers do not have good knowledge about what a child should do at what age. Providers have not taken special training in that specific area. Since we don't have special knowledge, we fail to consciously identify and treat those children.

Interviewer: What other barriers are there?

Respondent: I tried to mention that we only use maternal information to diagnose the child. We don't have the materials for further investigation of the child or to guide us. In addition, there should be a specific unit that works on this issue. To be honest, I have known about this concept in a nutrition course, but on the ground, we don't have a good practical knowledge about the condition. So, if there were a dedicated unit, all children who visit the health facility would have a chance to get a developmental assessment.

Interviewer: What are the facilitators?

Respondent: If there is a problem and barriers, obviously there will be facilitators. The good thing is, any healthcare provider who identifies a child with a developmental problem would not send them home without providing the minimum service. In addition, with the available equipment, we are ready to help the children.

Interviewer: What do you think of developmental monitoring for the future?

Respondent: I believe that any service that can identify such problems is appreciable. It should start working from each kebele, and specialized units should be established.

Interviewer: Who do you think is the most important family member to bring the child to the health facility?

Respondent: It’s usually the mother; if not, the father.

Interviewer: Do you have anything to add on this?

Respondent: No, I don't.

Interviewer: Thank you so much for your time.
