# Supplementary material for: Why are the implementation of and access to child developmental assessment within routine healthcare so difficult in Ethiopia? A descriptive qualitative study
Source: PLoS One. 2026 Aug 10;21(8):e0355686. doi: 10.1371/journal.pone.0355686 (PMC13456246; doi:10.1371/journal.pone.0355686)
Supplement: Supplementary file 2 [file pone.0355686.s002.zip › data/Transcripit 2.docx]

**Transcript 2**

Interviewer: Are you willing to participate and your voice to be recorded?

Respondent: yes, I am willing.

Interviewer: As an introduction tell me about the routine activities in the under-five OPD.

Respondent: Recently, we have been looking at emergency cases and for the time being cold cases are not coming that much. Most of the cases are dehydration and measles case. specially complicated measles cases. This shows that the vaccine history of the children is not full. We have been talking to work on this thing. They have follow up of ANC. They follow the ANC but the delivery is home delivery. we observe this and we are trying to change this trend. In addition to this, regarding vaccine they are partially vaccinated and it seems that is why they develop complicated measles. There are severe dehydration cases and We treat them and observe them at the isolation room for some time. Since the area is cold, febrile illnesses are not occur that much. There are also derma cases and malnutrition related cases. These are the commonest cases we observe frequently. If there are emergency cases, we prioritize this emergency cases and treat them. For those children who come having SAM(sever acute malnutrition) we treat them and let them stay for resustation. We communicate with SAM unit and discuss about the medical management. What we do here is to resustate and made them stable but we do not let them stay for long. They have to start F-75 at Sam unit we send them to there. We observe children up to 12 years of age here. Those who are above this will be sent to other OPDs.

Interviewer: okay, is there any other service the child could pass through before coming here?

Respondent: This is primary Hospital and things have been implemented to level it up to General Hospital. There are seniors and we General practitioners here. In some areas there are seniors. So, when I come to your question as this is primary Hospital, there are health centers under it. Most of the patients come with referral letter from the health centers. Those cases which can be managed at primary level will be managed. Those case which needs consultation with seniors, we consult with them and if it could be finish here, we will do that. But may be due to material like if we could not do investigations here like CT scan, we refer to other places. Most of the time we refer to Arbaminch.

Interviewer: Okay, where could they go after they register? Do they come to you ?

Respondent: There is a triage room. After they went to the registry room and take there card number, they go to triage room. They will be assessed there send to the appropriate OPDs based on their need. In order to come to us the age determines that. If they are up to 28 days, they go to NICU. If the age is greater than that and up to 12 years, we observe them. Currently we are working simultaneously of the cold case and the emergency case. They come to us passing through this.

Interviewer: when did you say a child has healthy development?

Respondent: It’s based on their age level and what is expected from them to do and pass in that way. If they do not go in that way, we consider it as delayance. It may need further investigation but if the child should have to grow a tooth but fails to do so or if the child is expected to start to communicate at his/her age but fail to do that. At the time the child is expected to start walking based on age but could not do that, we consider it as a delay. In order to identify the reason of the delayance investigations should be made.7:03

Interviewer: What are the most developmental related health problems a child encounter in this area?

Respondent: There are physical, mental and communication related delayance. Here in short time I encounter two problems which are related with physical delayance . One of the child was one year and five months old and have difficulty to stand. Most of the time when a child is one year old it’s expected that they should stand. The other child has communication related delayance which is related with language. There are catagories a child is expected to say like one word, two-word, one sentence and this child did not go as expected. So, this is the most common problems we observe.

Interviewer: From your observation at what stage the parents visit the health facility?

Respondent: In this area, the trend most of the time when something happens they go for traditional practice. For example, for a child who has tonsilitis they ‘ማስቡጠጥ’ and come when its very complicated or when its beyond there capacity or it becomes complex. They have this kind of practice.

In relation to developmental problem like when a child could not stand at the expected age or crawl at the expected age or walk at the expected age they think of a traditional thing like ‘wegsha’. If they do not see any change then they bring to the health facility. Most of the time it’s less likely to visit health facilities directly, especially in areas around the city or in rural settings. Those communities who had awareness or who are educated has good thing to visit health facilities. Otherwise, those in rural setting they come to us after they pass through traditional practices.

Interviewer: What services do you do to children to know their developmental status, currently?

Respondent: currently, unless the problem is complicated and it is not beyond from the capacity of this primary hospital, we provide the service here. We give supportive service and advice on things to be done at home. For example, related with cases with the vaccination we identify that and when the vaccine history is poor or the case is related with vaccination and in order to prevent similar things from happening, we put what we investigated and give education on that. For this specific child we give advice to avoid a repeated scenario. We identify whether the cause is related with vaccination or others things that cause developmental delay first. After we identify the cause, we treat it here if it can be treated but the causes that need further investigation and management or seniors’ involvement we refer.

Interviewer: In order to clarify the question for whom you do developmental assessment? How you do it?

Respondent: What we do is normally when a child come to us, we do assessment, and we ask for family history. We search for family history. On family history we ask whether there is anyone with similar situation. If it is a must and also needed to observe those family members, we tell them to bring the child. Sometimes if the cases are communicable if one child come, we tell them to bring other children at home. Like this in case of developmental delay if the underling cause is persistent and available in that area, for example if nutritional is the cause for the developmental delayance, we search for the things and look for the things to be done in that area and also look for family related issues whether there is someone elder and younger in the family with similar problem.

Interviewer: let me clarify the question you do all these things after you have identified a child has developmental delay, right?

Respondent: yes

Interviewer: so, in order to identify whether the child has developmental delay or not how do you asses?

Respondent: okay, there is no unit that works on to identify developmental delyance independently at primary hospital. But for example, in the hospital that I used to work which is compressive hospital at Hawass and I had also internship attachment there, it has its own unit like the other units such as DM unit, TB unit, RVI unit it has its own unit. Here at primary Hospital, it is incorporated with SAM (sever acute malnutrition) unit who are admitted. Most of the time the management is being give together with them. The assessment will be made simultaneously. There is pediatrics OPD, emergency and ward. The pediatrics ward there is SAM ward which works separately and it is called nutritional and rehabilitation center. It is managed there included as complicated SAM. So, when children are screened, they will be managed with malnutrition management simultaneously.

Interviewer: what kind of material you use for assessment?

Respondent: The guide we use, there are two types of guide. The first is the one we use for primary Hospital guideline which is the national guideline. So, with in it there is a section about development.

Interviewer: what is the name?

Respondent: Its primary hospital national guideline. In addition to this there is nutritional guideline for MAM (moderate acute malnutrition), SAM. For mild malnutrition it can be managed at the health center. Most of the time MAM and SAM cases are managed here, specially the sever one because they come to us by referral. Inline with this as I said before there is a guideline.

Interviewer: So, what are the domains included under the guideline?

Respondent: The four domains like physical delayance, communication or language, behavioral domain eeeee what is the fourth one… anyways the four domains are included. we follow that.

Interviewer: So, does the guideline available at your working area?

Respondent: Its condensed. As I said before since it is not a stand a lone service it is included under it.

Interviewer: Do you do the developmental assessment for every child who visit the OPD?

Respondent: It is for every child who come the assessment is being done, including those who have normal development. For those who has developmental delayance if it is manageable with us we do here as I said before but if it needs further evaluation /investigation or senior involvement we refer. Here there is no pediatrician, they come from Arbaminch University. There is no permanently employed pediatrician here.so those cases who need pediatrician involvement we refer them.

Interviewer: Can you say the service you are providing on developmental assessment is sufficient?

Respondent: It is not sufficient. Related to developmental deleyance first the service is condensed, the second is the way primary hospital is organized is not that much organized to do it. That means if it has its own unit, the one assigned to do at that area can communicate with the responsible body and develop so many things by training. Most of the time when a service is given in a consolidated way many thing can be competitive (ሚሻሙ) . Material supply is limited that much (በዚያን ያህል ውስን ይሆናል).

Interviewer: what do you mean by material supply?

Respondent: I did not go to there and its difficult to say this is available and this is not.

Interviewer: Okay, tell me in relation to your working place?

Respondent: Here at our level here most of the materials available are for emergency cases, but we do not have any material for cold cases.

Interviewer: In addition to what you told me earlier what would you do if you identify the child has developmental problem?

Respondent፡ Most of the thing we do is to refer the cases but what we do is supportive management. We do more on identifying the risk factors, the causes for it and on awareness creation and doing the screening. When we do the screening, we also ask whether there are similar scenarios not only in the household but also in the neighborhood. If there is someone in the village with similar scenarios we tell them to contact us. Mostly we work on the screening and creating awareness.

Interviewer: from the children who visit under five OPD for how many of them you developmental screening/assessment? Can you mention as a proportion from 100%?

Respondent: All children who enter this OPD, anthropometrics is done since it is considered as a vital sign, when we take the history and do the physical examination almost, they pass through it. when we ask nutritional history and the pediatrics history all the children pass through it but we give focus for those children whom we suspect.

Interviewer: okay, you told me earlier you have seen two children with developmental problem, right? Think of them to give answer for the following question. What does your interaction look like; Where do you do the assessment? Why? How long it takes?

Respondent: Normally there is the time to examine one patient otherwise for this kind of cases it needs additional time. we give emphasis first for emergency cases. After we resustate these cases we shift to examining children with this problem. As I said before one of the children has physical delayance in which he could not walk at the expected age and the cause we identify is poor nutritional status. He was severely malnuritioed patient. So, get treatment for it here and we link him with those who work below the health center level what we call them health extension workers. They are the one who work and facilitate most of the things related to malnutrition. We create link with them when we send the patients to them.

At the time I needed the child to had x-ray investigation but the x-ray we had is not working due to it reaches its service due date. So, it is made to be stopped working. I think the ministry of health officials has a plan to update it. At the time the service is not available here I send the child to Arbaminch.

Interviewer: What is the most common problem you encounter to do developmental assessment?

Respondent: For the second child as I told you he had language problem. So, at that time we done have any material to measure the language skill of the child. For example, in optamology unit there are material to examine if such kind of material available to assess the language skill of the child.

Interviewer: What is your concern while doing the assessment?

Respondent: The concerning issue to provide the assessment is the need of further investigation and the limited availability of materials. So, the limited availability of materials stops you from doing further evaluation. To tell you the truth the issues related with developmental delay did not get emphasis as I tried to raise earlier. Most of the time the issue is taken to be connect with the underline cause which is nutritional problem. The genetical cause of the problem is very rare. So, its connection with nutrition needs the involvement of different stakeholders. The problem is not resolved only by one personnel. You know it can make you happy when you fix the situation by yourself. So, the nature of the problem needs the involvement of different individuals and that is a concern.

Interviewer: From your observation: What is the most important factor at a health facility that could be barrier to do developmental assessment?

Respondent: The barriers to do developmental assessment is may be the patients do not come early to the health facility. The good thing is if they are connected with the health care providers, the providers feel the problem or if they get the chance to contact the health extension/health post or the health center they do not let them stay longer. They send do us. We also do what we can at our level. The service that is given at different level is interesting.

Interviewer: what are the barriers at facility level?

Respondent: The barriers, one starting from the place there is no a separate room and in addition human power is another barrier. The first thing is room there should be someone assigned to work there. For example, in nutrition management there are assigned professionals like that if there is someone who is assigned as professional for developmental assessment more if this can be done either by short term training or to update professional to work in the area. As one of the barriers I mention human power related issue. The third is materials necessary for it. As I mentioned earlier the four domains are the one to be assessed. So in order to assess the four component it is essential to identify what material we need is.

Interviewer: what about the barrier related with the professionals?

Respondent: Regarding the professional the gap on exposure to the case.

Interviewer: Can you explain this a little bit?

Respondent: on identification of developmental delay in general they are working on the currently available things. But, recent things, data at national, international level, regional level or zonal level or in areas like the information is not available at the hands of the professionals. So, the gap is available there, its information gap. The one thing is that.

Interviewer: what about the other things?

Respondent: other things…. the thing related with developmental as I told you earlier its linked to poor nutritional history, poor vaccination so that since these things are not alleviated or these things should be alleviated as a country and may challenge the countries capacity. Those responsible bodies should work on it. having nutritional problems should be identified specially those having sever nutritional problem. In relation to developmental problem in order to identify this problem it would be easy first to identify areas having nutritional problem. I take the source of the problem as nutrition related and if it is worked in that area.

31:29

Interviewer: From your observation: What is the most important factor at a health facility that encourage to do developmental assessment

Respondent: The facilitators eeeee there are trainings. Even if it is specifically not on developmental assessment but on pediatrics related trainings, SAM related training is organized. That is the good part and, in that training, they touch the developmental areas. That is the good thing. Most of the trainings are linked with NGOs and that is a good thing even to update the professional and share the experience of what is being done in one area to the others recently. To show who is going in a good way and who is not at zonal level. To share the experience of one to the other. In addition to the information they provide, those who give training, they look over which organization is going inline with the program and which is not. it is a good part.

Interviewer: What do you think of changing the current mode of developmental assessment to developmental monitoring by using screening tool?

Respondent: This is very good. I always give attention for such kind of researches. I once face such kind of individuals while I was intern. They were working at national level from ADDIS Abeba and ADAMA. We raise important points and on the last section you give suggestion, at that point you give your suggestion for different stakeholders. You show the gaps and what should be done there. To tell you the truth the researches in Ethiopia does not go beyond the shelf even if we are seeing changes know a day. In addition, it has a good memory that you can talk about as I had participated in research for PHD student.

So at least if there is a tool or guideline, we do not stay in one area for too long, we had rotation every six month. You see me now at OPD but after six months I might go to NICU or surgical ward. If the tool is available everyone who comes can work by using it.so such kind of thing available it is important.

Interviewer: what is the benefit for the children or for the provider?

Respondent: let me start with the providers, it avoids subjective evaluation, put clear direction and minimize things that might be missed. Everyone could not do different things or a mixed-up thing because they can use the same tool. It also minimizes the missing rate like doing GCS (Glasco coma scale). There is a list of command so you do not miss anything. Like this if there is a clear guideline you do not miss anything. In addition, it will help in way that all organization have similar service delivery. It helps to avoid the service to be delivered in one organization not in the other.

This in return benefit the patient. It prevents delayance in receiving the service the patient needs to have before it is complicated. It also prevents waste of money and time by early detection and intervention.

Interviewer: What would be the possible challenges?

Respondent: From the attendants or family or patient or community side you can face that it can be related with lack of awareness because most of the time they relate this thing with spirituality or traditional belief. So, they stayed for long engaging in such kind of things.so it needs working on feeling the gap on awareness.

From providers perspective the challenge could be interest. Everyone who comes to work in this area should have interest as long as they come to help or serve the community. However, from out of them in the hospital the one who has the very interest should work in this area effectively from all the other professionals. Most of the time in pediatrics care its suggested that those who can provide the best care are encouraged to be part of it. The best service as well as best professional who had interested and devoted provide pediatrics care They should be selected and assigned to work there and this could be a challenge.

The overall challenge could be as a government level is financial problem. In order to organize and provide so many things budget is required and it could be another possible challenge.

Interviewer: How do parents know their child has developmental problem before they come to health facility in this area?

Respondent: Its good to start identification of such problem before a mother is pregnant. Most of the time in our country mothers come after pregnancy occur for ANC. But, if possible, it should be started before a women become pregnant at the time, she plans to get pregnant. Its good if they come with enough information. Based on this if the community has awareness on development and participate in early identification of the problem by coming to the health provider. This makes things easy. In order to detect first in relation to the health extension workers doing vaccination, hygiene and communicable disease they give education. if they incorporate this and give education by making this as one component. If awareness is created and next to this on the ANC this issue is incorporated its good.

Interviewer: okay thank you, let me clarify the question how can a mother identify whether her child has health development or not at home?

Respondent: okay from my experience working hear or in my previous working place, most of the time the first born are exposed for the problem because there awareness about the problem is limited. If they do not have the chance to compare the child with their brothers or sisters, they could have difficulty to identify the problem. if the child is the second child they compare with the first born. They say when does my first born started to stand alone, when did he started to run, hike or things like this. So, they related this child with the first born and question why this child does not start talking while the elder started to talk at the same age? So, they identify the problem by comparison with the others.

Interviewer: Think of the two children and what was the parental concern at the time?

Respondent: As I told you earlier, they connect this thing with spiritual problem. when they come to us first, they think as the problem is very complex and relate it with so many things. They had a feeling of regrate like they are the one to put their child into this. we tell them the scientific reason for the occurrence of the problem, and teach them what is expected from them to do.

Interviewer: what is the most frequent question they ask?

Respondent: Good, let us take infertility for example people think infertility has no cure or it has no solution unless it has spiritual intervention or through the holy water. But in the health sector there is a chance that means they might had cured or not. There is similar attitude for developmental problem like infertility. They belief it is already the end or as it does not have any solution or treatment. We told them then it can be treated once the cause is identified and they keep working on it.

Interviewer: what are the challenges while you refer them?

Respondent: Regarding referral for all cases the problem is connected with finance .The financial related concern is what they raise otherwise they are happy with the service.

Interviewer: Who is the most important family or community member in seeking developmental services?

Respondent: From the parents, on the front line is the mother, next is the father then those in the household or in the community. Those who has awareness about the issue are responsible.

Interviewer: how?

Respondent: The role of the mother starts from the ANC and then after on nutrition, exclusive breast feeding, sunlight exposure, providing balanced complementary so she is the one to take responsibility for these things. The father is responsible to cover the financial need to go to the health facility.

Interviewer: Thank you so much for spending time with me
