# Supplementary material for: Why are the implementation of and access to child developmental assessment within routine healthcare so difficult in Ethiopia? A descriptive qualitative study
Source: PLoS One. 2026 Aug 10;21(8):e0355686. doi: 10.1371/journal.pone.0355686 (PMC13456246; doi:10.1371/journal.pone.0355686)
Supplement: Supplementary file 2 [file pone.0355686.s002.zip › data/Transcripit 20.docx]

**Transcript 20**

**I: Interview**

**R: Response**

I: As per your information, you are willing to take part in this study and volunteer to be recorded?
R: Yes, I volunteer to participate.

I: Okay. In the first part, we will discuss child development–related issues. As an introduction, under the Under-5 OPD, what are your daily tasks? What do you do?
R: Okay. (Laughs) First let me introduce myself. My name is Dr. Bekele Barza, General Practitioner at Birbir Health Center. We provide all services, and the Under-5 OPD is one of them. In the Under-5 OPD, we see all cases. The major ones are diarrhea, pneumonia, malnutrition such as stunting, and sometimes developmental delays. All cases for under-five children are handled in this OPD.

I: Okay. Now, you treat many children. When do you say that a child’s development is normal? What are the manifestations?
R: To decide whether child development is normal, we generally observe their physical appearance and age, and check whether the child is acting according to developmental milestones. There are milestones from infancy upwards. We check if the child’s age matches those milestones. Here we take weight for under-five children and check age–weight appropriateness. If a child is malnourished, we identify them. When such cases happen, we have a stabilization center here. We treat them with formula prepared for malnourished children. When they gain weight and meet discharge criteria, we discharge them. Sometimes we also see children with Down syndrome. They have typical physical features and sometimes cannot walk or stand even at three or four years of age. We diagnose developmental delay and link them to a hospital where there is a pediatrician.

I: Okay. What are the signs of developmental delay?
R: Developmental delay is broad. It can be psychosocial, physical, or related to communication. Most of the time, children come after three or four months with physical palsy—their legs and hands may be paralyzed, and their neck cannot support the head. Their age and physical activity are mismatched. Children with Down syndrome have different features—nose shape, tongue, forehead. These are external signs. Internally, there may also be heart deformities. For example, by age two, a child should speak some words. Children with these problems cannot. They also show inappropriate social interaction. These are developmental delay manifestations.

I: What common developmental delays do you often see in this community?
R: Mostly what I already described—what we call facial palsy, paralysis of hands and legs, lethargic appearance, inability to support the head. They may look like they have poliomyelitis, but they don’t. Another common cause is malnutrition. During infancy and after complementary feeding, even if they look physically okay, their development may be delayed. Delays can be congenital (like Down syndrome) or related to poor maternal care. Malnutrition-related delays can be treated here, and children recover—start walking, performing social activities, etc. We compare them to peers. If a six- or seven-year-old cannot do what a two-year-old can, that is a delay.

I: Which age groups are most affected?
R: Mostly one to two years. Families wait until one year and then say, “This child is two years but cannot stand,” or “one year old but cannot sit without support.”

I: How severe are the problems when they come for treatment?
R: It depends on the family’s educational status. Educated families come early. Rural families may come later, often after two years while coming for another medical case, not for the delay.

I: How is the service being delivered?
R: In our health center we deliver services in the immunization center. It used to be a maternal waiting room; now it is the under-five immunization room. Children come for vaccination every Tuesday until 15 months of age. Weight is measured and other conditions assessed. If something is abnormal, they are linked to us. We have weight, anthropometry, height, and length measurements.

I: What about children older than 15 months?
R: They only come if they have a medical case. We don’t do routine developmental assessments for them.

I: What materials/tools do you use for assessment?
R: We mainly use our clinical assessment. We do not have materials specifically for developmental delay assessment. Under-five care includes MUAC, weight, height, and length. The rest is clinical evaluation.

I: Do you think the service is enough to assess developmental delay?
R: No, it’s not enough. Developmental assessment needs additional materials and trained professionals—neonatal, pediatric, and under-five trained staff who know milestones. Our guideline is general.

I: What guideline do you use?
R: Mainly anthropometry and the under-five guideline. It includes brief age-specific descriptions and pictures.

I: Do you assess developmental delay for all children coming with medical cases?
R: No. We assess only based on age, physical appearance, and social activity. For example, a two-year-old who cannot walk is delayed. We look for causes. We do not assess all children because the environment is not suitable and there is a lack of professional capacity. We are very busy—50 children per day with only two nurses.

I: What proportion of children receive developmental assessment?
R: Maybe one case from many. There is also community stigma. Families hide their children. Educated families bring them; others don’t. Maybe one case per month or every two months.

I: Describe one case you remember.
R: A child with facial palsy, general weakness, no communication. The family was educated and requested referral. I know more than three such cases.

I: What triggers you to assess developmental delay?
R: Physical weakness, inability to support the head, inability to sit at age three. We measure height, weight, and refer for imaging like CT scan, which we don’t have. Down syndrome also has clear features. We refer such children.

I: What setup is suitable for developmental assessment?
R: Trained providers, psychiatric professionals, neonatal nurses, and a conducive environment. The work requires patience and proper space.

I: What challenges occur when you disclose the diagnosis to families?
R: Families already know something is wrong. It is painful for them. There is stigma—they may hide the child. Some accept the condition and seek care; others rely on prayer or spiritual beliefs.

I: What strongholds and weaknesses exist in the health system?
R: The system is not supportive for developmental assessment. There is no comprehensive guideline, no dedicated clinic for developmental delay, no referral or treatment system, and policies do not focus on developmental delay.

I: IMNCI 2021 includes developmental delay.
R: Yes, but information reaches late. Training and updates are insufficient.

I: What opportunities exist?
R: Immunization follow-up allows multiple contacts within 15 months. Good community awareness of vaccination helps us observe children regularly.

I: How can the service be improved?
R: Start from ANC with nutrition counseling, ensure safe birth and good neonatal care, promote breastfeeding and complementary feeding, strengthen health extension workers to follow households, train healthcare providers, and improve referral systems.

I: What is the importance of having a standard tool for developmental delay assessment?
R: It would help identify children whose families do not recognize delays. We now rely mostly on family complaints.

I: What challenges may occur when using such a tool?
R: Low community acceptance due to stigma, and our health system is not ready to manage identified cases.

I: Where do families go when they suspect developmental delay?
R: First to spiritual options—church, confession, prayer. Medical care is often the last option, sometimes after two years.

I: After coming to the health facility, what do families like or dislike?
R: Dislikes include lifelong burden, stigma, and economic challenges. Likes include the possibility of diagnosis and treatment.

I: What are families most concerned about?
R: “What is the root cause?” If treatable, they want treatment; if congenital, it is difficult.

I: What services should be available at the community level?
R: Strengthen health extension workers to know every pregnant woman and under-five child in each household. House-to-house follow-up is crucial for early detection and prevention of developmental delays.

I: Thank you very much.
R: You didn’t introduce yourself.
I: Thank you.
