# Supplementary material for: Why are the implementation of and access to child developmental assessment within routine healthcare so difficult in Ethiopia? A descriptive qualitative study
Source: PLoS One. 2026 Aug 10;21(8):e0355686. doi: 10.1371/journal.pone.0355686 (PMC13456246; doi:10.1371/journal.pone.0355686)
Supplement: Supplementary file 2 [file pone.0355686.s002.zip › data/Transcripit 3.docx]

**Transcript 3**

Interviewer: Are you willing to participate and your voice to be recorded?

Respondent: yes, I am willing. Based on the information you gave me it’s a very important issue in our country and I want to provide information.

Interviewer: As an introduction tell me about the routine activities in the under-five OPD.

Respondent: In under five most of the time starting from the neonatal period to and those who come for immunization. we provide treatments for the routine medical cases and also children who come with malnutrition. Some women come to consult for their children. For neonates there is a separate service but for those who are after the neonatal period to five years we provide medical services. Sometimes we provide minor surgical services. In addition, we provide nutritional treatment service by appointing them every month.

Interviewer: whom you give monthly appointment?

Respondent: Most of the time when the condition is sever, we refer to Arbaminch Hospital because we do not have F-75 and F-100 here. But, when they reach at the stage to be treated by plampy nut they will be sent back to us. Also, we screen children and those who have moderate malnuitrion they will be given monthly appointment and treated with plampynut.

Interviewer: can you please tell me about what healthy growth and development is?

Respondent: A child is considered as having a health development is when he can do in terms of thinking, cognation as what is expected from him at that specific age like that of his peers. There can be some deviation either lower or higher from that but if the child is stick on what is expected we know he has a health development. Whereas the growth is about the body growth or height which is expected to have as the peers or children at the same age group, we say the growth is healthy.

Interviewer: from your experience What are the signs of unhealthy development that children have?

Respondent: There are many children who have follow up here and they have unhealthy development. Most of the time children who are five years old but they do not speak or communicate and other who have the signs of under developed cognitation. In addition, there are children who are two years old and cannot start walking and there are children who did not have teeth by one year.

Interviewer: From all this what are the commonest problem?

Respondent: most of the time its common to see children who have difficulty in communication in related to their age. There are children who come with communication problem as what is expected from there age group.

Interviewer: How do you know if someone has developmental problem in your area?

Respondent: the families identify the problem in reference to the peers. When they made mistakes comparing to the peers the family bring them to us. Most of the time in our profession and those at the lower level identify the problem grossly without evaluating by material. we do clinical evaluation to identify those children who have deviation and put our assessment.

Interviewer: At what stage the families bring the child at the health facility?

Respondent: Most of the time they bring at the end stage after they try different things like traditional and spiritual things at the last stage after the child is big.

Interviewer: What services do you do to children to know their developmental status, currently?

Respondent: we use here most of the time manual things. We measure the height by meter and the weight-by-weight scale. Sometimes for those children who had lower kilo in comparison with other children with similar age group we do these things. Some parents describe the development in relation with peers to us.

Interviewer: let me clarify the question, when we talk earlier about growth and development you described the different domains of development .so the question is how do you assess that?

Respondent: okay for that we do not have any tool to assesses that but we assess in the form of interviewing the parents. Specially those children who are 4- or 5-years old children we ask some easy questions like his name, his friends name and others just for the seak of evaluation and to see how he answers the question not to measure the IQ. We do this just to know whether the child is deviating or not from the topic by asking simple question. Otherwise, we do not use special tools to identify this.

Interviewer: you do this for those who speaks? How do you assess the other domain?

Respondent: yeah, for motor activities we ask if they write on paper or move at home or do something at home. we do not do other things than asking whether they do this these things. we do not use other tools.

Interviewer: why do not you use the tools or guidelines which have been used in other health facilities for assessment?

Respondent: okay regarding that, this is a primary hospital which starts working in a recent year and it good be due to the lack of experience sharing or training regarding the topic.

Interviewer: How do you describe the current developmental assessment way in this facility?

Respondent: we use a poor evaluation method which show the problem has got less concern and its not considered as a public health important. This may be due to the rare nature of the problem and it did not get that much concern. I think this might continue util someone starts to advocate about it.

Interviewer: Do you think the service you are providing to identify developmental problem currently is sufficient and timely?

Respondent: It is not sufficient. It should get more attention.

Interviewer: can you explain more?

Respondent: The work should start from the lower level and you can do nothing at the higher level only regarding the service provision. I believe that in order to maintain a good result we should use an approach that should work starting from the lower level. The other thing is in order to say the service is sufficient there should be a set up which has every material and works separately to provide the service.so it needs special services.

Interviewer: For whom you do developmental assessment?

Respondent: Our way of doing the assessment is selective. Here we see medical cases. So, if children come with medical condition and if there is nothing that is visible grossly, we take history. Based on the history if we find children who had a repeated attack of infectious disease, who had feeding problem, change in the hair color and other related things, we base this information in order to do the assessment to screen the development. We are selective.

Interviewer: What proportion of children do you think will come to a health facility receive developmental assessment?

Respondent: Is that daily, monthly or totally? Totally we do the assessment for 40- 50% of the children.

Interviewer: What do you do when you know when a child has developmental problem?

Respondent: Okay if the child has developmental problem, most of the time those families at low economic status encounter such kind of problem. Since malnutrition is the problem of low-income countries and those families who have the lowest in come as well those who are living with highest family member come with such problem. Mainly developmental problem is related with malnutrition; we advise the parents to care for this child. In addition, we treat infections. We give monthly follow up and evaluate the change the child has. if its decreasing or some may continue in the same phase so we separately discuss with the family

Interviewer: what do you do for those who had developmental problem?

Respondent: For those we advise the family that it may take time for the problem to heal and we suggest them to go other facility for a better care where there is a specialized professionals in order to discuss. We advise about schooling that some of the parents do not want to send the children to school.

Interviewer: Do you have the chance to meet children with developmental problem in this facility?

Respondent: yes, I meet with three children with such kind of problem

Interviewer: can you explain that?

Respondent: the first is a four-year-old child who could not walk. His body is not strong. The other could not communicate with the peers and the families and he had Autism-like symptoms. The other is a one-year-old could not stand by himself, eat by himself and he has also down syndrome.

Interviewer: Think about a child who received developmental assessment service, what does your interaction look

Respondent: when I assess it takes 30-40 minute and I found him when we could not communicate, eat food by himself or finish what is given for him. He plays by himself only and he had autism-like symptoms. He has additional illness.

Interviewer: What is your concern while doing the assessment?

Respondent: It that he had additional illness, it’s difficult for me to understand the condition by looking just the baby. Other than this the family has hidden the child for long time. So, it was difficult for me to get some information.

Interviewer: what about other thigs?

Respondent: At the time to examine the mental status evaluation but I did not.

Interviewer: why you did not do this thing?

Respondent: its because there is no standard tools which could help to know the problem. They come and receive the service like any other child. if there is special tool to do assessment, its good.

Interviewer: Think about a child who you want to do developmental assessment for and failed to provide? Did you experience such thing?

Respondent: yes, there was and its was due to the crowded nature of the set up. There is no a separate set up to do the assessment. The room is highly crowded by children. Also, the number of staff and children is disproportional.

Interviewer: Think about a child who you provide but the parents refuse to received developmental assessment service

Respondent: some of the families are willing to receive and some resist to receive .when they are told about cognitive related problem they perceive it as it is a life long problem and scare .So they get angry and be hope less about the child due to awareness problem. It was difficult to discuss this with them.

Interviewer: From your observation: What is the factor s at a health facility that could be barrier to do developmental assessment?

Respondent: In health care system starting from the management its not given a special concern and arranged things. In addition, there is no trained professional who could manage when such kind of cases come to the health facility. The set up is not organized in that there is no a separate room for it. In addition, the health professionals lack to give attention considering the problem is rare. It is not considered as a common disease.

Interviewer: Can you add some other information?

Respondent: In other hospitals it has its own separate room as I worked at referral hospital. The professionals work without getting bored and there was a good working environment. There was a means to even support the families. So, it is a well developed and organized service. If there is a separate room at General and primary Hospital level and at least one profession has training on it and work there. So that if we find out such kind of case we can link with them.

Interviewer: What do you think are the factor at providers side that could be barrier to do developmental assessment?

Respondent: Regarding the professional most of the time related to development, it could be due to awareness or not considering it as problem, they do not give much attention. Even if they give attention, they do not take time to examine the child.

Interviewer: why?

Respondent: Because they give time for the commonest cases.

Interviewer: Tell me about the knowledge and skill of professionals related to developmental assessment?

Respondent: There is a higher level of skill gap on this. In developmental assessment you have to evaluate complex things and in order to do that it needs specially training. Because of this they neglect to do the assessment. I think there is a gap.

Interviewer: From your observation: What is the factor at a health facility that encourage to do developmental assessment?

Respondent: Regarding professionals when they found out one child with developmental problem, it takes/wastes so many things from the family like time, money and other things compared to healthy children. So, it needs to provide special support for the family and it should get attention/concern. The family thinks of the outcome and support should be given. I by myself give concern for such thing. I tell the parents to bring the child even when he had a little problem and to give more attention than the other children. Because they do not express themselves.

Interviewer: What is the factor at a health facility that encourage to do developmental assessment?

Respondent: for developmental assessment the system is poor related to economical issues. It needs huge economy. In the other conditions with a little expense, you can do so many things but for this it needs a big expense to provide the service. So, the system is not as such facilitator to provide the service.

Interviewer: what other thing things you add?

Respondent: It should be done on the society awareness on the community-based outreach activities. So, it should be added as an activity for the providers going out for outreach activities to teach on this problem.

Interviewer: What do you think of changing the current mode of developmental assessment to developmental monitoring by using screening tool?

Respondent: the benefit is to standardize the service in the health care systems, which is not yet implemented, if this kind of service come it will allow the service to be given in a better way as the other services. It helps to aware the providers that this kind of service available.

Interviewer: what is the benefit for the provider?

Respondent: For the providers it add one additional skill.

Interviewer: what about for the families?

Respondent: For the family it allows increase the utilization of health care.

Interviewer: what are the possible challenges?

Respondent: The possible challenge could be, most probably this is the area which could get many supports, but the challenge could be more financial challenge.

Interviewer: what is the reason for not coming early to receive the service in the health facility?

Respondent: The main reason is the community has its own belief that they consider it is demon or evil, so they perceive that the child could not be healed other than the spiritual intervention. It’s related with such kind of attitude. The other reason is those who are key individuals in the community tell the parents to give time and look, then the child can be healed and tell them taking the child to the health facility at this time could not bring any change. Its attitude problem. They finally come to the health facility when this all things fail. They come to medical treatment to try something new and better. The challenge is the attitude.

Interviewer: What do they like most?

Respondent: What they like is the advice that we give them based on our experience. It is good that they are informed about what would happen in the future and that made them happy. We also told them the things to do and the care to give in the future. These things give them comfort but hear that the outcome may not be as good as they expected cause discomfort.

Interviewer: What is the question they ask?

Respondent: Most of the time they ask finally after they hear the result. They ask whether the child has hope to be cured or not. what is the future hope, can they work or not? They had concern related to this.

Interviewer: what about during referral?

Respondent: when we refer, they feel the problem is sever and they become scared.

Interviewer: Who is the most important family or community member in seeking developmental services?

Respondent: The mothers, grand mothers are the one to bring the child.

Interviewer: Thank you
