# Supplementary material for: Why are the implementation of and access to child developmental assessment within routine healthcare so difficult in Ethiopia? A descriptive qualitative study
Source: PLoS One. 2026 Aug 10;21(8):e0355686. doi: 10.1371/journal.pone.0355686 (PMC13456246; doi:10.1371/journal.pone.0355686)
Supplement: Supplementary file 2 [file pone.0355686.s002.zip › data/Transcripit 4.docx]

**Transcript 4**

Interviewer: Are you willing to participate and your voice to be recorded?

Respondent: yes, I am willing.

Interviewer: First tell me your daily routine work in under five OPD?

Respondent: we are providing all services here in the health center. Among these, under five service is the one. The common cases seen in under five OPD include diarrhea, pneumonia, malnutrition related services, and developmental delay cases. Here we have only one under 5 OPD.

Interviewer: When do you say a child has a healthy development? Why?

Respondent: generally, to say a child is on healthy development we look physical appearance and check appropriateness of developmental millstone with age and developmental stage. We usually measure weight, height and compare with age to check appropriateness. Malnutrition cases with weight for age being inappropriate treated in the health center. For such cases linking with stabilizing center found in our health center is being done until full filling discharge criteria. Sometimes Down syndrome cases also diagnosed. Those who have developmental delay referred for hospital for more care.

Interviewer: What are the signs of unhealthy development?

Respondent: can be manifested with different signs like, physical palsy like unable to control head and weak extremity, poor communication for the age which are Common in Down syndrome cases.

Interviewer: which developmental delay type is common in this area?

Respondents: developmental delay with facial palsy, weak extremity like poliomyelitis case, unable to support head which usually eligible for CT scan to look which part of the brain is the palsy affected. Developmental delay related to malnutrition with inadequate maternal milk intake, problems related complementary feeding also common. As I said earlier facial palsy is developmental. Developmental delay due to maternal care in age greater than 6 months is being treated here at nutritional stabilization center. Normal physical appearance, start of walking, social act, and start peer communication are indicators of improvements.

Interviewer: which age groups most affected? At what stage they visits health institution?

Respondents: mostly at 1-2yrs of age is being visiting facility having the case. Most families wait until 1 year hoping that the child can start walking and improvements expecting. After 1 year family starts to consult health providers. Stage of visit to facilities depends on families’ awareness and educational status. Those awared families visits early but rural family get visit lately even came with other medical issues.

Interviewer: How do you provide developmental assessment either in the community or health facility?

Respondent: the service is given during vaccination visits for those age under 15. Anthropometric measurements like Weight, length, and height measured during the visit and relate this with age. During vaccination those child who have health problems including developmental delay, linked to providers in the institution.

Interviewer: how age >15 yrs. can get the service?

Respondents: only when they visits with medical case generally no service specified for these groups.

Interviewer: how do you assess the domains of healthy development? What materials you use to perform developmental assessment?

Respondents: mostly clinical assessment done. Anthropometric measurements taken,

Materials: MAUC, length, height

Interviewer: In your observation do you think the service you are providing timely, adequate? Why? HOW??

Respondent: not enough, materials needed, well oriented providers with training, pediatricians, and pediatric nurse. To assess developmental assessment developmental millstones using guide line need to be well understood. Related to time most case came institution after some delays.

Interviewer: what guidelines are available to assess developmental millstone?

Respondent: age specific criteria supported with picture is available to asses developmental mile stone found in under five guide line.

Interviewer: for whom developmental assessment done? Do you do routine developmental assessment for all children?

Respondents: developmental assessment done as I told you earlier, is being done based on age, physical appearance and socially. For example 2 year child who fail to stand and walk general have developmental delay and the cause need to be identified either developmental or genetic. Age in appropriate appearance is important in developmental assessment in our case. Family members also worried for those for example age >2yr who can’t stand, walk, stay in lying position, even who defecate on bed clearly have developmental delay. For such cases developmental delay assessment is being done.

Interviewer: why not developmental delay assessment not done for all?

Respondents: I believe developmental delay assessment need to be done for all. Practically this is not being done in our facility. The first reason is the condition is not convenient to the assessments. The other is related to provider’s burdens. There are many case visiting the facility and to complete this providers rush the assessment. Those visiting under 5 usually weight take others wise the focus is on the complaint the child is visiting the facility.

Interviewer: What proportion of children do you think will come to a health facility receive developmental assessment?

Respondents: in our case limited number for example 1 out of 100 is being assessed for developmental assessment. Actually the community also need not want to expose such cases. Only family expose children with developmental delay when the child has developed diarrhea or fever. Very limited family members usually the educated groups seek service for developmental delay. Most families kept children with developmental delay isolated socially and hide inside house. A maximum of two cases per month is being assessed.

Interviewer: ever diagnosed children with developmental delay? How do you follow the child?

Respondents: yes, one case diagnosed had facial general weakness which is flaccid, and not communicating I remember visited the facility being his family educated and diagnosed which final referred.

Interviewer: for children assesses and diagnosed, how was the assessment and how suspected for developmental delay?

Respondents: usually came with physically flaccid extremity like polio case, can’t support their head. We referred those case to center having pediatricians. For those with malnutrition, malnutrition treatment given in the facility. After improvement and weight gain we referred to center having pediatricians.

Interviewer: what was the challenges faced during assessing cases? What is the most common problem you encounter to do developmental assessment? What type of set up is needed to do developmental assessment?

Respondents: physical assessment done, weakness checked, weight and height measured. After this we referred these children for CT scan. For example Down syndrome cases, they drop their tongue, have inappropriate ear set and cardiac malformation which arise a need for referral.The set up needs well trained providers in the assessment including psychiatrists, neonatologists.

Interviewer: what was the challenge on side of family when they told the case?

Respondents: actually it is painful to family having child being lacking behind with his peers. Most families already knows when visiting the facility. And some accept and ready to further treatment. But there are family members who hide and kept such case in the house without consulting and getting treatment. Actually developmental delay treatment in Ethiopia is very difficult due to the set up and treating brain palsy is very difficult. Even if the cases are not prevalent as infectious diseases, I think the case is prevalent.

Interviewer: what are the barriers to do developmental assessment at the health system?

Respondents: one is motivation of health care providers since the assessment is time taking and needs patience, and provider’s ignorance. This might be related with lack of training including updates and motivation and high burden of patient flow as 50 cases per day is being seen with 1 or 2 providers only in one OPD. If appropriate assessment flowing the procedure to be done about 1 hours is required for 1 child which is impossible with high client flow. In adequate number of the provider also on barriers.

The system not encouraging to do so. At system level there is no guide lines regarding child assessment. Developmental assessment by itself needs its clinic and teams managing this. If developmental delay assessment clinic presents, more assessment can be done. Nothing is done on this problem and already is ignored.

Interviewer: the issues is included in 2021 new IMNCI guide line, what can you tell me about it?

Respondents: information are not reached on time and training updates are rare.

Interviewer: what are the good things to provide the service? Facilitators for developmental assessment?

Respondents: not as such encouraging but there are many opportunities to get children during birth, at immunization and up to 45 days many visits for each child which gives chance to assess developmental assessment. Awareness on vaccination improved at community which gives chance for assessment.

Interviewer: how can be the service improved?

Respondents: need to start during ANC focusing weight and counseling on maternal adequate nutrition. Then child follow up, adequate breast feeding and complementary feeding on 6 month need to give attention. On clinical side orientation training, increase number of staff, and improving referral system needed on developmental delay.

Interviewer: what do you think is the important if standard tools are ready?

Respondents: this improve the diagnosing the problem and more cases can be diagnosed.

Interviewer: what do you think the challenges to give the service? When families told the problem, what is their response? When they visit facility?

Respondents: community acceptance on the problem, institutional set up, availability of treatments for diagnosed cases. Mostly they perceive as other thing like curs, and they give priority for praying and the like. And when medical cases occur, they visit facility. Social influence, economic Burdon, social attitude are stressful conditions to families. Families hide such child when someone gets enter the house. Families frequently asks the reason for this problem,

Interviewer: What type of services need to be given at community level?

Respondents: health extension programs was important previously. During their visit they knows every pregnant woman, every under 5 and follow immunization well. So, strengthening this system is important.

Interviewer : Thank you
