# Supplementary material for: Why are the implementation of and access to child developmental assessment within routine healthcare so difficult in Ethiopia? A descriptive qualitative study
Source: PLoS One. 2026 Aug 10;21(8):e0355686. doi: 10.1371/journal.pone.0355686 (PMC13456246; doi:10.1371/journal.pone.0355686)
Supplement: Supplementary file 2 [file pone.0355686.s002.zip › data/Transcripit 5.docx]

Transcript 5

Interviewer: Are you willing to participate and your voice to be recorded?

Respondent: yes, I am willing.

Interviewer: Tell me your daily routine work in your coordination unit regarding child growth and development?

Respondent: Regarding child development there are so many activities specially by under-five teems and its under maternal and child health unit. So, there are so many activities and the first is to measure the weight and height to see their development. we check whether the child is feeding on breast properly we check the change in weight. We also check the change in weight by asking the mother how much is the weight of the baby at birth or from the registry we look for her previous weight and her current weight. For those who are one year and above there are other nutritional screening. we do this thing and look for there development.

Interviewer: okay before we go to further discussion can you tell me the difference between child growth and development?

Respondent: okay the difference between growth and development, when we say growth, it can be about the body but when we say development it includes the body as well as the mental component.

Interviewer: okay from what you stated development incorporate growth and the mental component. So how you do developmental assessment?

Respondent: eee about developmental assessment, we identify such kind of issues while they come for vaccination at the immunization unit. In addition, when they come being sick by other cases to OPD. This is how we identify children with developmental problem. So, they are different from normal children by having something debated.

Interviewer: How do you know if someone has developmental problem in your area?

Respondent: In order to identify the problem for example some children like there is an expected activity that they should have done for example for six month or nine-month child there are activities. For example, a six-month baby is expected to sit by himself and a nine-month child is expected to start to stand and walking. So, these problems can be detected when they come for immunization of measles or may be when they come for other treatment. So, we identify them at this time.

Interviewer: Do you have any guideline to use?

Respondent: we just do interviews like’ does your child start speaking or does he start calling for some words?’ We do not have any other guideline. At OPD level it can be available but at immunization unit there is no guideline.

Interviewer: When do you say a child has a healthy development? Why?

Respondent: I say the child has normal development starting from birth if he is free from reasons that can hinder the development or that can stop his life and then after in his day to day living if he did not encounter any problem, for me it is healthy development. I say it is not normal development when at birth he has anomalies. Those children having anomalies could not develop or grow to their full potential. They have there on consequences. In the procedures done to solve these anomalies may be there could have a short term or a long-term damage. A few numbers of Children from many could survive this and develop in a good way.

Interviewer: What are the signs of unhealthy development?

Respondent: The signs that I consider if the child has developmental problem is for example in terms of system, I could say he has not a well-developed cognation. if call them or order them to do something and they fail to do that we can differentiate.

Interviewer: What are the most developmental related health problems a child encounter?

Respondent: The most of the time I encounter here is that they say “ it has been this month since my child is born but could not start standing alone, she did not start to talking ‘.Then we reassure the condition and if it could not be done in our set up we refer for better health care.

Interviewer: How do you know if someone has developmental problem in your area?

Respondent: As I told you earlier, most of the time they have their own means of identifying as my child is growing it for example, they consider the child is developing when she starts walking or starts speaking a word .so may be if they fail to sit by themselves, they consider it as a problem.

Interviewer: How could they identify or know it’s a problem? What is there base?

Respondent: As I told you they consider starting to walk as a development.

Interviewer: At what stage of the problem do you usually know the child has developmental problem?

Respondent: They come before it gets complicated. I believe that the current situation that we have is taking us to a better thing. That mean in the first-place mothers give delivery at health facility and after delivery she could have so many follow up at the health facility. So, at that time the health professional or other providers could tell her that to bring the child immediately if they notice any problem. So, she bring the child immediately. So they come early before it gets complicated.

Interviewer: What are the programs that your organization implementing to identify children with developmental problem?

Respondent: okay, our facility is hospital and the health center is closer to the community. There are also health post and there are campaign for some disease like for measle. So, we collaborate with those campaigns and we tell them to bring children with this kind of problems. The first is campaign which is can be immunization for measles or other. The other is deworming. So, when this campaign is available by collaborating with this we have been working. Other wise we got this children while they come for other treatment to the facility. In addition ,we get those in our catchment while giving birth here. But health centers and health posts are more involved in this thing.

Interviewer: okay let me clarify the question As you know there so many programs in the healthy system for example ANC, immunization. So is there such kind of program for developmental assessment?

Respondent: no there are no programs

Interviewer: How do you explain the available infrastructure/logistics to provide the service?

Respondent: Regarding infrastructure it has not started to be given as a separate unit. We provide the service at under five opd. It has no its own unit.

Interviewer: can you explain the logistics?

Respondent: Related with logistics there are not enough materials. There is a meter to measure their heigh .There are no materials other than this.

Interviewer: what about the working environment?

Respondent: As I told you earlier it has no a separate unit. If there is a case it needs special care

Interviewer: How do the professional do the assessment?

Respondent: If there is a case we do the assessment as a routine case. May be if we got a different finding we refer to the general hospital.

Interviewer: What are capacity building activities done so far regarding developmental assessment?

Respondent: Regarding to capacity building the professionals did not get any special training which is on child growth and development. However, the professional are working by the knowledge they had from the education.

Interviewer: How do you explain the skill and knowledge of providers to do developmental assessment

Respondent: I could not say they have a full knowledge but they are working by using the knowledge they have. They use whatever opportunity they have to work on this thing.

Interviewer: In your observation do you think the service you are providing timely, adequate?

Respondent: No its not adequate.

Interviewer: why?

Respondent: Because first as I said before they are taking the service at under five opd together with other services. if we do not give focus for it ,we may miss things or as I said we could not see the growth and development of the child properly.

Interviewer: what would you do if you know the child has developmental problem?

Respondent: when we know if the child has developmental problem it can be due to medical problem or it could be due to hormonal problem that they get from their mother during birth. If we identify this thing the set up is not as such proper so we refer them.

Interviewer: From your observation: What is the most important factor at a health facility/health system that could be barrier

Respondent: The first thing is the skill and knowledge of the professional. The other is related to the logistic. The logistic is not sufficient to provide service and also the infrastructure is not sufficient. This things stop us to provide the service.

Interviewer: what about a health facility/health system related barrier?

Respondent: Regarding the health system barrier it is not being provided in a separate unit and lack of a stake holder or other organization that funds this program like the others. So, there is lack of donner that help children who had such kind of problem to get further treatment. If the system could be available to the lowest level.

Interviewer: What other factors be barrier to do developmental assessment?

Respondent: At system level the problem has not given emphasis and this could be due to the occurrence of the problem is very few in the community. I think the reason for the less emphasis is this.

Interviewer : Can you clarify “what you say the occurrence of the problem is few” can you estimate the occurrence from 100 children.

Respondent: I do not think the occurrence is that big it could be 1% or 2%. I think the reason is this.

Interviewer: How do you do the assessment? Is that for all children or when you suspect?

Respondent: All children who come to the health facility screened. For example, in immunization its requested to register the developmental milestone status of the child.so all children are screened and if the child has the problem on the registry we tick that. So, when someone who wants the data we provide from the registry.

Interviewer: what the name of the registry?

Respondent: It is the immunization registration or EPI registration.

Interviewer: From your observation: What is the most important factor at a health facility that encourage to do developmental

Respondent: The facilitators eee as an organization its our work to identify such kind of cases and inform. It’s our duty and our day-to-day activity. However, as a hospital or organization there is nothing that could help us to facilities our work related with this. There are things I mentioned before related with infrastructure and financial issues. We have no capacity to pass these things.

Interviewer: what about providers side facilitators that encourage to do developmental assessment

Respondent: The professional are ready if things are available.

Interviewer: what other facilitators?

Respondent: if training is organized which are more related with this. So, far we do not have training. If the responsible body facilitate this and the provider got the training in order to build their knowledge and let them provide knowledge-based service.

Interviewer: What do you think of changing the current mode of developmental assessment to developmental monitoring by using screening tool? Tell me about growth monitoring first

Respondent: All children who come to the under-five OPD monitor for their growth. At the time that we monitor the growth for example, in our set up there are things we do not have and we do the monitoring for those children who had malnutrition but after some time there is a project that order all children who enter the under-five OPD should be monitored for all parameters of their growth. So, children who are identified as having the problem then classified and get their treatment whether its SC or the others.

Interviewer: So, tell me in relation to about changing the current mode of developmental assessment to developmental monitoring by using screening tool

Respondent: if I got the chance to change things the first thing I do is to organize the infrastructures. In addition, I will try to organize training for professional.

Interviewer: let me clarify the question what would be the benefit of changing the current developmental assessment in a way to be delivered in a monthly base with standard screening tool?

Respondent: okay if there is a guideline, their own schedule and a monthly follow up, it helps to identify children who has the problem but stays at home. There can be children who has the problem but stayed at home due to lack of information or missed to get such kind of opportunity. So, if there is a separate unit it can increase the chance of get this kind of children. In addition, it facilitates to provide service and education for those children.

Interviewer: What would be the possible challenges?

Respondent: Regarding knowledge they may connect the issue with none medical conditions like this is from devil or this thing comes from heredity .They might have such kind of thinking and if they come to us we can educate and change such kind of perception.

Interviewer: What about in the health facility?

Respondent: when you open a separate unit it could not be a routine service and needs man power and many financial issues due to the extra working hours like providers ask per Diem. So this things need finance

Interviewer: What are the possible facilitators?

Respondent: Identifying such kind of children , knowing there problem and helping them by informing the cause of the condition could let them change there way of thinking .so looking this could be a facilitator.

Interviewer: In your observation what do parents do when they know the child has developmental problem?

Respondent: what they do first is they ask the neighbors, families they say ‘this thing happened what is it ‘.so based on the answer they get they go for the next action.

Interviewer: Where do they go to get health services?

Respondent: They go to health facilities. I could not say all come to health facility. As I said before they ask family members who is educated and ask where to go. The answer they get led them. If they had a good led ,they go to the health facility if not they go to other places like tradition healers

Interviewer: Can you explain about the traditional service?

Respondent: They related such kind of problem with heredity from the mother or the father also they perceive as the father or the mother have a curse. So they relate and take them to psychics’. In addition, they use traditional herbals.

Interviewer: Is there any one who do such thing at the near by?

Respondent: no there is no one here but at the rural setting there is.

Interviewer: At what stage do they seek health care?

Respondent: From the children we face so far we did not get children who come to us at complicated stage.

Interviewer: What do you see as the barriers for parents to access child development assessment?

Respondent: The first thing is the knowledge they have about the problem. In addition ,unavailability of a different service after they come here as a separate unit. For example, they consider all the units has a separate investigation service. when we say go to ANC they know where to go. So, its a challenge that the service is not available at a separate unit.

Interviewer: what about others? What are the differences in access to child development assessment (monitoring)?

Respondent: if we give level those who have better knowledge and better income come to receive the service in early time. Those who had lowest income fear to come by saying they would ask me the highest payment so they do not come for the service.

Interviewer: Who is responsible for resolving barriers? why?

Respondent: The responsibility is the governments. If it makes the system cost free they might use.

Interviewer: Who is the most important family or community member in seeking developmental services? how?

Respondent: The father and the mother or the whole family are responsible. In addition ,those in the community who are respected are responsible to guide and educate them. The responsible of the father is to support financial ,prepare things and made decision. The mother has a role to identify the problem as ‘our child should start to do this thing but he/she does not’.

Interviewer: Thank you so much.

Respondent: Thank you.
