# Supplementary material for: Why are the implementation of and access to child developmental assessment within routine healthcare so difficult in Ethiopia? A descriptive qualitative study
Source: PLoS One. 2026 Aug 10;21(8):e0355686. doi: 10.1371/journal.pone.0355686 (PMC13456246; doi:10.1371/journal.pone.0355686)
Supplement: Supplementary file 2 [file pone.0355686.s002.zip › data/Transcripit 6.docx]

**Transcript 6**

Interviewer: How is child development monitoring being implemented in this health facility?

Respondent: We will see the weight in the form set by the Minister of Health when the children come for vaccination. Now, for example, we ask the mother the status of the child in the next visit after she has her current visit. We do not leave by providing only the vaccination we measure the head circumference, height, and we check for there growth . We also check the status of the child. we are working like this.

Interviewer: Are you providing this service only for children who come for immunization?

Respondent: We do this for every child. Sometimes children could not come to this room and if there is no professional at the opd there card still come, so they will be taken to this room (the immunization room) because here there are material to do the examine. They come to receive the service here.

Interviewer: Do you think the service you are providing currently is timely and adequate?

Respondent: No, it is not. In terms of infrastructure, the health center does not have its own dedicated room. Currently, we are providing different services in the same room at the same time, and we have also done this in the past. This arrangement is not appropriate for the children or for maternal, especially regarding privacy. It is difficult to say the service is appropriate for shecha Health center. To say it's convenient is difficult; we are constantly moving from one department to another.

Interviewer: How do you follow the children?

Respondent: Yes, if a child coming to under-five clinic for care, they get treated and they leave. There is nothing we call "reached." It means doing monitoring. Because it's for vaccination, it's only if there is a defaulter. It's when we do defaulter tracing. But we are not very focused on "where is the child's growth?" We are more focused on "how is their vaccination schedule progressing?" It takes priority. But there is something lacking in connection with their growth.

Interviewer: What developmental monitoring and assessment practices have been observed in work with children and their families?

Respondent: I will be honest on this. If I receive training on this topic, I will give training on the topic related to growth/development, that activity will continue on that topic and you will be paid. Also, this kind of service also comes from something that has monitoring, it will exist as an activity. If payment is missing, there is a lot of hesitation. For people working with children, something timely in terms training should be given; everyone wants to be paid properly for their work. At our place, this matter doesn't exist. Because of this, they are in a state of negligence. Even if it's done more or less, it cannot be said to be 100%.

Interviewer: What is the family's participation in the monitoring?

Respondent: Yes, there are different types of parents-some educated, and some are not. Most parents pay attention to their child’s age and expect certain milestones, like walking. When a child has not started walking at the expected time, they begin to wonder, ‘What is happening with my child?’ or ‘why has not this happened this month?’. If the child has already started walking, parents usually donot see it as big issue. Even if the child is a bit late, many parents are not very concerned. From what I’ve observed, families expect a child to walk when they reach the typical age of walking. For example, when the child turns one, they may ask,’ What happened? Why isn’t my child walking yet?’. Parents only see it as a problem if the child cannot walk when they believe the child should be able to-unless the child is sick. Generally, parents donot monitor developmental milestones closely unless they are more educated.

Interviewer: how do you involve them in the place where this service is being provided?

Respondent: We need to involve mothers. For example, when they come in, we assess the child based on their age. If the child is four years old, we ask questions such as: ‘What is their approximate weight?’ ‘What problems have you noticed?’ How are you caring for your child?’ ‘What is their food intake like?’ and ‘Under what conditions are you providing their meals?’ We do awareness -raising work on these issues.

Beyond this, I donot believe there is much else that has been implemented. When children appear to be developing normally, we usually donot give many additional instructions. But if the child is below expectations or shows signs of delay, we ask, “what are you feeding them?’ and provide guidance such as “From a nutritional perspective, you should provide food like this and this.’ If the issue is related to breastfeeding, we also give appropriate education on that topic.

Interviewer: How do you provide the subsequent follow-up service?

Respondent: This is not very common. For example, saying “their growth is like this,” or “their developmental delay is noted here and should be addressed in this way” is rarely done. As I mentioned earlier, assessments mostly happen during vaccination visits or when the child is sick, but there is very little work specifically focused on developmental delay.

Interviewer: What things can be described as barriers to implementation?

Respondent: T o be able to implement the work properly, screening is needed. Before starting any intervention, we first need to screen children to understand their situation. After the screening, we can determine how to begin the work. It could be done through a formal program, but currently, this is not happening. So before we can start anything we conduct screening. As for what can be recorded in the family’s component, since very little screening is being done. I cannot say much.

Interviewer: What are the other barriers?

Respondent: Training is one of them. After you receive training, you may continue for two or three years, but there are not many updates. For example, in this health center, trainers donot come regularly. Another issue is materials- we donot have complete materials. We have some, but to say we have 100% of what is needed would not be true. Even in terms of the room itself, if the materials were complete, the space might be insufficient. But since it is all being done in a “half-half” way, it is not adequate.

Regarding staff, supervision must be done regularly. A person who took training, after 3 years and 4 years, science is always updating. It happens that they think "I have already taken it." But a staff doesn’t raise the issue of competency on training. "We need to do supervision," they say. Like in this health center, the trainings are not optimized. Also, it's said "take training from another place," but it's not always available

Interviewer: Are there trained personnel specifically for this? Connected with it?

Respondent: Currently, the professional who received the training are limited-about four or five in total. Two of them are permanently assigned to this department. One of them is responsible for data entry.

Interviewer: Now, what do you think is the biggest problem?

Respondent: Although there are trained professionals who can be assigned, under -five children are not consistently meeting with staff who have received this training. A shift system is used, but the staffs who should be responsible are not always the ones providing the service. I have already raised these issues: at the under-five clinic, there must be a dedicated professional. Children under clinic-five,there must be a dedicated professional. Children under five need to be seen by someone properly trained.

According to the guidelines, the ideal approach is for the professional providing the service to be someone who has completed the training. We have discussed this closely, and starting from the next budget year, if duty assignments are made, we can arrange for trained professionals to work in their designated positions. We have already discussed this, and adjustments will be made in the next period. But for now, this system is not yet in place.

Interviewer: What about other barriers ?

Respondent: It is something that attention has been diverted from. Before, we didn't mention it much. We say immunization, we say defaulter, but "this child is delayed," "they should have grown at their time," this matter, from a health perspective, "why haven't they talked?" "Why haven't they walked?" is not the focus, it's on other things.

Interviewer: Why do you think that is?

Respondent: Why do I think? First, in our area, it is not very common, it happens occasionally. But now there is a lot. And from the beginning, perhaps it wasn't seen, or it wasn't studied. It could be like that. I think it's a matter that hasn't been studied myself.

Respondent: In relation to health institution, staff are often assigned to outreach duties. When this happens, if a professional who is responsible for a particular job is called for outreach duties, even important activities are interrupted so they can go and fulfill that duty. Because of this, the work does not become routine, and the professional cannot carry out their responsibilities consistently.

If a professional were permanently assigned to that specific job, it would clearly be their responsibility, and there would be no question about why they are not doing it. However, because the role is linked to the health center and staff are frequently taken out for outreach activities, the work is often left unfinished. Therefore, this situation creates an additional challenge for the professional and affects the continuity of the service.

Interviewer: We have listed the problems. And now, what things could encourage us or facilitate our implementation?

Respondent: The things can facilitate implementation include support from the health center. For example, when professionals are assigned to a site, they need certain resources. If they are sent to the field, they need payment. With out any motivating factors, the work will not be done, and the professional may leave the assignment. If the necessary support is not provided, they may say ,’I can’t do this’. From the professional’s perspective, being assigned toa duty along with an appropriate budget motivates them and enables them to do their work properly

Interviewer: If a child has a developmental delay, until a medical diagnosis is reached, what do you do?

Respondent: If we identify a developmental delay, we look into what caused it. We ask, ‘what brought this thing?’ and “What is its origin?” because developmental delays donot happen without reason. A delay may be related to illness or nutrition. Understanding why the issue started is essential, because making an accurate diagnosis is what an appropriate solution.

Interviewer: what way can these things be captured so that we can bring a better service?

Respondent: First, starting with myself and the department-under-five services require additional support. As I mentioned earlier, other necessary components must be in place. For example, if we are going to admit children or provide services, we need an appropriate and convenient space to do so. We have repeatedly requested an additional room for our department, but to this day the issue remains unresolved.

Currently, we manage by working in one shred room, rotating shifts and adjusting schedules. If the under-five department were fully independent, with its own dedicated space, this would greatly improve our work. From the department’s perspective, when the budget year begins, the needs of this department must be properly assessed and addressed. Not only through external decisions, but also by considering my own input as a leader.

Regarding professionals, as I mentioned earlier, staff must be properly assigned to the site. To make the work more effective, the health center and its management need to consider, “In what way should we assign professionals?” If they review this and set a clear direction, and then implement it; it would greatly improve the service.

Interviewer: Thank you so much for the time you gave us
