# Supplementary material for: Why are the implementation of and access to child developmental assessment within routine healthcare so difficult in Ethiopia? A descriptive qualitative study
Source: PLoS One. 2026 Aug 10;21(8):e0355686. doi: 10.1371/journal.pone.0355686 (PMC13456246; doi:10.1371/journal.pone.0355686)
Supplement: Supplementary file 2 [file pone.0355686.s002.zip › data/Transcripit 7.docx]

**Transcript 7**

Interviewer: Are you willing to participate and your voice to be recorded?

Respondent: yes, I am willing. As long as it is to protect the health condition of the community it’s okay, I am willing.

Interviewer: As an introduction tell me about the routine activities in the under-five OPD.

Respondent: In under five OPD it has its own protocol and guideline. Directions are set on the guideline and based on that we do the assessment and do additional checkups. Also Based on it even the drug that we prescribed is based on the direction on the guideline. We work by using this protocol.

Interviewer: Do you have guideline for all of the things you do in under five OPDs?

Respondent: There is IMNCI protocol and on it there are list of diseases .so the guideline gives a direction as do this, assess this so we assess based on it and again there are things you are expected to check out and treat. It puts the treatment to be given. So, we give based on the guideline starting from 2014 in Ethiopian Calander.

Interviewer: okay coming to our today’s discussion point, when do you say a child has a healthy development? Why?

Respondent: okay healthy development starts from pregnancy. During the time of pregnancy if a mother uses adequate nutritious food and if she has her health follow up properly. From the time she become pregnant to the day she gives birth there is an expected weight she should gain I think it is around 18 kg. The way she feeds and has health follow up also help her as well as the baby she is expecting to give birth to be healthy. So, from the time she gets pregnant to delivery and after delivery tile two years if the nutrition is good in all dimension like mental health the development is good. If there is something missed in between this problem will happen. Childrens fail to have healthy development in terms of cognation or in the other dimensions. So, this are very essential things especially the first 1000 day of the child is very important. its very related and starts and continues from pregnancy.

Interviewer: What are the signs of unhealthy development?

Respondent: okay from the time of birth that means from zero to 2-month ,2 month to 4 month and like this till 18 month and above it there is expected things to do. So, if the child has missed to full fill what is expected from him to do, we assess that too. In between these nutritional issues assessed.

Interviewer: What are the most developmental related health problems a child encounter?

Respondent: Normally, we did not face such kind of problem most of the time but we face children with malnutrition. if the child has no good nutrition its known that so many problems will happen. Here related with the living condition, the people only consider giving birth not on the other things related necessary for the child after birth for example if it is a must for the child to receive protein and fail to get one, he would be exposed to other thing. This thing again could lead to other problems. Here we face so many malniutired children but we don’t encounter children with developmental problem.

Interviewer: How do you know if someone has developmental problem in your area?

Respondent: family?

Interviewer: yes, family.

Respondent: In relation to development for example at the time the child is expected to start crawling but the child did not start, they mention my child does not start crawling while his peers start crawling this showed there could be some problem.so they say please look the child. At that time we start to identify what kind of problem there is. Is there feeding problem? If the child has feeding problem for example exclusive breast feeding is recommended tile six month, then after six month exclusive breast feeding is not sufficient alone. So, they should start complementary feeding. Then we say in addition to the exclusive breast feeding there are recommended additional things but you did not do this these things or these things are absent. In addition, sometimes, if they are economically disadvantaged that means if they do not even had food to eat for themselves, they ignore to prepare foods for the child. Because of these children could expose to malnutrition or to the expected development for his age. So, these things cause damage.

Interviewer: At what stage of the problem do you usually know the child has developmental problem?

Respondent: sometimes they come after they had severe complication. Then based on the protocol we measure the weight for age. If the condition is sever, we treat them by F100 or F75 at the stabilization center. Otherwise, we treat the others at the outpatient by OTP and plumpnut. In addition, we counsel to give the child additional things at home and we follow every week. we tell them that the OTP is as treatment and it does not have any continuity.so we counsel them to adjust their feeding style.

Interviewer: What services do you do to children to know their developmental status, currently?

Respondent: About the developmental assessment that all professionals may do the assessment or not. Its different from one individual to the other. The assessment done by those trained professional is different from those who are not trained. Those trained individuals could work based on what he has learned and the guideline and check the listed expectations the child should full fill based on the guideline. For example, the child is expected to full fill this thing on this age or do the child has missed what is expected him to do in his previous months? Or not does he feet for the current age or not. So, you check all these things.

Interviewer: what is the guideline?

Respondent: The treatment guideline which is IMNCI, all things are available on it. Its developed based on age and has sections based on age. Also, there is the expected direction a child should full at a specific age.

Interviewer: Do you do developmental assessment for every child?

Respondent: It’s a must to do the assessment as per the guideline for every child who comes for treatment. It can be for follow up or they come as sick, it’s a must to do the assessment because the guideline order to do so. We check for developmental milestones, anemia, malnutrition TB, HIV, there are so many things. This are under the guideline and the developmental component is introduced in the recent years. In the previous times you only measure weight and height but in recent years like about two years the developmental component is introduced because it is essential. Based on this it is a must to assess.

Interviewer: Previously you have mentioned you also check the previous history of the child? Would you please clarify that.

Respondent: ooh that one we ask whether the child has full filled what is expected from him at some age previously before he come to here. If he full fill to do what is expected from his age its okay if not we consider him as he misses to do what is expected from his age.

Interviewer: So, you ask the mother?

Respondent: yes

Interviewer: you also mentioned earlier the developmental assessment is introduced in the guideline because its essential, how is it essential?

Respondent: yes, I said before because child development is essential. because starting from the time one mother gets pregnant till she gives birth and till the child growth, our body needs so many things. So, for these things should be prepared early and it’s a must to work on development. If we fail to work on it, it will be a challenge for the future life, and so many other things.so it is essential.

Interviewer: What materials you use to perform developmental assessment?

Respondent: the material we use related to nutrition is MUAC, weight scale, other thing will be assessed by asking the mother.

Interviewer: what about for developmental assessment?

Respondent: can you clarify?

Interviewer: There are different domains of development as from our conversation earlier, right? So how do you do the assessment for each?

Respondent: We do not have any other material in addition to what I mentioned earlier, we just ask and get information from parents. We do not have any other material but we know that head circumference should be measured if we suspect something.

Interviewer: you had the training, right? So, what materials are important for the assessment.

Respondent: on the training after attending the theoretical sessions we get the chance to have practical session at some health facilities but we do not have access for the material but the focus was on the theoretical concepts.

Interviewer: can you clarify what you mean by access?

Respondent: on the training sites the children come or may not come at the facility. we did not do any additional thing other than looking for the children at the time.

Interviewer: In your observation do you think the service you are providing timely, adequate?

Respondent: No, it is not sufficient.

Interviewer: why?

Respondent: It is not sufficient because there should be something practical thing that we should have engaged at. Also, there should be a means to confirm your diagnosis. The information that we receive from parents or the mother may or may not right. But its good that if we have things to do practically by using different material. If there is a material for assessment it can strengthen our diagnosis by giving justification to come to conformation. Its better if such things are available.

Interviewer: What do you do when you know when a child has developmental problem?

Respondent: if there is a developmental problem if there is anything a child has missed things related to his development in the past, we could not do anything for the past but we try to adjust things for the future. We provide follow up and professional support on what we expect on he should do at that specific age. Otherwise, if we could not do anything in our set up we will refer to other settings. 15:48

Interviewer: What proportion of children do you think will come to a health facility receive developmental assessment?

Respondent: at least 50% of the children get the assessment service.

Interviewer: Think about a child who received developmental assessment service, what does your interaction look?

Respondent: I do not experience a child suspected for developmental delay so I do not do the assessment.

Interviewer: From your observation: What is the factor s at a health facility that could be barrier to do developmental assessment

Respondent: okay, on the previous question which is on how many of the children get developmental assessment from 100 children, there is a shortage of trained professional. I am the only one who had the training. If I am not available someone will examine and write something on the registry. So, the service has some gaps in that the one I provide and the others is different. At least if there is someone who stays the whole day working in the area and if there is material that can be used and help to strengthen the service to be provided here. This thing will help to provide an improved and better service. The shortage of professionals by itself limits the quality of the service to be provided for the people equally. In addition, if there is material that confirm our assessment as I told you earlier rather than basing our assessment on parental response. In order, to provide a well-organized service different material will help us to provide a better service as well the community to receive a better service.

Interviewer: What do you think are the factor at providers side that could be barrier to do developmental? In addition, with the previous barriers at the health care system.

Respondent: By the way the responsibility to organize training is not the health centers, there are other bodies. the organization just report for the responsible stake holder as “we had shortage of trained professional” in this area. It just let know the responsible bodies the number and list of individuals to participate in training that will be scheduled on different topic based on the gap in the organization. The decision of training is given at the zonal and regional level. They are the one to facilitate things. For example, every year it is a must to have training and one individual has a responsibility to serve for one year after training. So ,it is the problem of the zone and the woreda if they fail to provide training. In addition, there is turnover of trained professionals and these all cause a gap.

Interviewer: what about other reasons? As you told me earlier only the 50% of children receive developmental assessment and the rest does not, so what is the reason for this?

Respondent: okay in under five children treatment there is shortage of drugs or treatment and it’s not easily accessible. In addition, there are treatments that come by donation from EBSA and others, they are available for some times and lost for some. This can make you treat the patient in less satisfactory way and cause a gap. Also, sometimes we run out of materials for example currently there is no weight scale in under-five OPDs and we are on the process to get another one yet. Now we use the weight scale of the adults. First, we weight the mother and the baby together then we weight the mother and the baby separately then we subtract to get the weight of the child. Lack of materials as well as disorganization of the service limits us from providing a good service.

Interviewer: From your observation: What are the factor at a health facility that encourage to do developmental assessment?

Respondent: I think having professional who are ready to serve is one facilitator and also having trained professional is another.

Interviewer: What other factors be facilitators to do developmental assessment?

Respondent: nothing

Interviewer: What do you think of changing the current mode of developmental assessment to developmental monitoring by using screening tool?

Respondent: This is a very good thing for example in the old days there was a service what we call it CBNC if you know it. It is a monthly service that was provided at community level. Every month under five children in the community measured their weight, and screened for problems. Also, we checked whether the weight and the height are appropriate for that specific age. It also helps to identify whether there is a gap or not. It has been done at kebele level. When they come to us for treatment, we do measure the weight. But when you announce at the kebele level all children could come and be measured. At that time, you can identify who has normal development appropriate for their age, who had moderate problem, and who has sever problem. At that time, you get the chance to counsel the mothers on what to do for the child. If such kind of things incorporates and available it a good mode of delivery, this thing was introduced in the old days by NGO or some other organization and they handover it to the health care system and stop it. If the health system could not handle it in a good way it fails.

Interviewer: what is the other benefit?

Respondent: May be…motivation is a good thing for a human being and if those mothers who bring the child could be encourage by different means it is better. For example, there was an organization called alive and thrive working on nutrition bring things for children. For example, they prepare the plate in a way that shows what foods the child should feed on. It’s prepared in a colorful way with pictures and the mothers get training on that and how to feed their children. Then when they went, they give the mothers a plate. So, this things encourage the families to buy the foods necessary for the child by using the money they have .even they sold some of their materials to buy food. If such kind of arrangements are made in the community it will motivate the mothers to participate in identification of the problem but it may have economical challenge. Here compared to the husband, the wife is highly involved in the household situation. Overall, we could not do such kind of thing in our set up and in our capacity unless the NGOs has a means to initiate and support such kind of activities. In addition, if the providers are motivating to do so it will be easy.

Interviewer: What would be the possible challenges?

Respondent: okay the possible challenge could be related to finance in order to full fill most of the services and fiancé accounts 70% of the challenges out of 100 %. if there is no budget it would be difficult. For example, in order to demonstrate and show the mothers how to feed the children……we did demonstration on how to feed the child from the money raised from the community. we even build demonstration settings then we practically showed food was prepared from each food group like protein and other nutrients which are thought to be necessary for development at health center level and kebele level. If such kind of things available like fund to facilitate some activities, then we can educate the community. The community can also do what they can even use what they have at home.

Interviewer: In your observation what do parents do when they know the child has developmental problem?

Respondent: if there is developmental problem as I told you earlier if one child lags behind his peers. They ask each other as what is wrong with my child? After this they discuss on who could help us? Who would tell us? At the community level there are health developmental armies on one to five arrangements. So, based on that chain they speak that there is problem with X’s child. If they screen the child at the time and find out there is a problem, they bring to us. At the facility we do the assessment and tell the mother as the problem with your child is this, these things and you have to do these things. if we can do the follow up we also tell that to her. So, they speak to each other at the community and exchange information.

Interviewer: Do all of them come to the health facility?

Respondent: ooh those of who has a clue and at the near by do come. Currently the awareness of the community is different from the old times. For example, in the old days they do not know about vaccination, for malnourished children or if the child has swelling on legs they do ‘Asity’ (burning the skin by fire) and they engage in so many things. But nowadays after the health extension program has come there are so many changes. The health extension workers, health development armies and the one to five arrangements have a meeting every 15 days and talk if there is problem. if they find a problem, they will bring to the health extension worker and then she brings it to us at the health center or other health facilities. But in some rural settings there is a gap. Currently, we are under Gerse city administration but previously there are so many rural kebeles under our catchment which are hard to reach. At that time there are different problems. But we are serving few kebeles and have less problem.

Interviewer: In your opinion what is the attitude of parents towards developmental assessment?

Respondent: the parents are happy when you do things for their child. They are eager to know so many things about their child and the health of the child. When parents come to any health facility to receive health service, they are happy when you do so many investigations. Even when you do a few investigations and send them home they would be dissatisfied but if you measure weight, height, MUAC and so many things they say this facility is good, the professional working in this facility is good. But if the child has not as such a problem and you rush things, they would not be happy.

Interviewer: when you do developmental assessment what are the parental concerns?

Respondent: okay normally when they come to the health facility, they already accept things but sometimes when we told the mother your child has this this problems they worry a lot. They ask how can I solve this thing? Some of them have no money even to buy things to eat. Even if its not allowed to give the flour for everyone , we consider this mothers. Due to the seasonal changes now we are encountering different problem know but we recommend this mothers to be supported.

Interviewer: Did face a mother who refuses to receive developmental assessment service?

Respondent: No, we told the mother this thing should be done for your child. As along as they come to us they did not refuse to receive the service. They want to know about the problem of their child .so they do not refuse.

Interviewer: what things they ask about?

Respondent: if it takes a longer time while you do the assessment, they say what is the problem with my child? If there is a problem, we tell them the reality and counsel them if you do this thing, you could help your child. So, we tell and counsel them about the reality. Then you hear the feedback from them. However, they consider their child has received the right care when you take your time to do each and every assessment. when there is a high case flow some want to stay for long and some do not. The recommended time for a client to stay in the health facility is 30 minutes. Some want to went out immediately and some want to take time for their child to have a detail investigation.

Interviewer: Who is the most important family or community member in seeking developmental services? how?

Respondent: Both the husband and the wife and any one in the family is responsible. It’s the responsibility of all. As long as they want and accept the child to be born it’s their responsibility. So, they have to take care of the child. The husband should provide the money to buy things and the mother should give her time to take care of the baby. It’s the responsibility of all. The mothers are good at caring for the child at the household. She knows each any every stage of the child’s development but the others could not do that. The mother is the one nearest to child. If Both are employed workers there could be a Nany. Overall it’s the responsibility of all.

Thank you so much
