# Supplementary material for: Why are the implementation of and access to child developmental assessment within routine healthcare so difficult in Ethiopia? A descriptive qualitative study
Source: PLoS One. 2026 Aug 10;21(8):e0355686. doi: 10.1371/journal.pone.0355686 (PMC13456246; doi:10.1371/journal.pone.0355686)
Supplement: Supplementary file 2 [file pone.0355686.s002.zip › data/Transcripit 8.docx]

**Transcript 8**

1. How is child development monitoring being implemented in this health facility?

**Answer:** We monitor the development of children by measuring whether their age is proportionate to their weight and whether their weight is appropriate for their age.

Okay thanks but this is what we call it growth monitoring; my question is about how you monitor the different child developmental domains.

- It is by asking the mother and by looking at the child's activities. For example, can he walk at the age expected him to walk. we ask the mother and we also look at them.

1.1.is there any standard to classify the child on there developmental concern?

**Answer:** no we do not have such kind of thing.

1.2. You are expected to report about child development on the IMNCH right? How could you do that?

**Answer:** It is by looking and asking the mother, there is no manual other than that. I have neither trained nor experienced the new IMNCH guide, the one I am applying is the old one. I use it.

1.3.Do you think that initiating the service after the mother has concern sufficient to identify developmental problems?

Answer: no its not

Why?

Because, for example, when we ask a mother, "mothers say my child do things late, my children start walking late” she might say that my children start crawling late. This may not be enough, what if the child continues like this.

The service we provide is not enough. I always think that even if I get one case, where should I refer to. For example, if I get a 4-year-old who can't walk, where can I send him? For example, a 4-year-old child has to play, I haven't experienced it yet but what would I do if I found one. I always think this thing.

2.As you told me you do developmental assessment by using your clinical knowledge if you suspect and the mother has concern. Also, you told me the service you provide is not enough. What do you think the right way of assessment should be?

**Answer:** For example, there are danger signs of other treatments, or what we call children's danger signs. If we see these danger signs in children, there is a recommended immediate action to be taken. If there is this kind of signs, for example, a sign of child developmental delay. I think it would be good if there was a guide to do something after you see these kinds of signs.

2.1. This kind of guide is available on IMCHI ,why don’t you use it?

**Answer:** Because I haven't taken the training, I don't know if the other professional who recently took the training might apply it. But I believe all children who visit the clinic should be assessed. We first measure the weight and then we go into history.

2.2. How do you involve care givers or families in the developmental assessment? Because you told me you initiate the developmental assessment based on the family concern?

**Answer** ፡ Most of the time, what we do the assessment when we see the signs. when we see the sign we start questioning. We do this when we suspect the child development is delayed, but if he is an active child, we do not enter into that question. Involve the family only when we see the signs. We engage them by asking.

2.3. Do you have any schedule for follow up?

**Answer** ፡No child has taken the services with us yet. There is follow up for other diseases, but for this kind of problem we don’t have. We haven't face it yet, but there was one child who came with the problem while I was working at the health post. When she come she is very thin we thought it was nutritional problem and give her plapnut but for two to three months she has no change .Then we refer her to hospital. I hear they give her milk and I left the site. But it seems she has a development problem.

3. Would you please tell me what the barriers are to provide the assessment?

**Answer** ፡ when I do this, for example, there is no many things. but when there are many children, we do not take weights, heights. We will be loaded and we do not follow the requirements. If the number of patients is high we don’t even take MUAC ,we do that if we suspect the child has the problem. We do not give much attention to look at the child if the case flow is large.

3.1. Do you mind telling me your knowledge about developmental assessment?

**Answer** ፡ I don’t think I have enough knowledge about developmental assessment. I just give suggestion about it or say do this or that but I am not sure to make decision on developmental related problems.

3.2. What are environmental or material related barrier?

**Answer** ፡ It is not comfortable, for example, the materials should be colorful, this on the wall should be color full to get the attention of the children not to make them stressful. The weight scale should be colorful. But the environment is not favorable.

**3.3.How can we solve this barrier and who would be responsible?**

**Answer** ፡ I do not know may be we don’t bring the problem to the health center. The responsibility starts from me because I am working on the place but I did not bring the problems to the manger and it starts from.

We have done nothing to improve the problem. I was the child health focal person in the past and we asked some material and also about the room but due to shortage of budget this things are not fulfilled.

3.3. What do you think about the mode of delivering the assessment? Do you think what you are doing now is sufficient or do you have any other suggestion?

**Answer** ፡ For example, if one or two experts are here, it is possible depending on the season. There are times when patients are not there even for me to work. There are times when there is over flow of patients and its good if two providers are assigned. I think we can do better by completing all the necessary things from the room and then examining the MUAC. I think it is better if all professionals training.

It is very important. We have skill gap now. If we don't take the training, we will have a skill gap and we will not be able to treat the whole thing better. Therefore, it is better if first we get training and second professional be committed to provide better treatment.

3.4.what do you think of using a standard screening tool?

**Answer** ፡In the middle of that, we can find children with developmental problems. For example, if it is something like a campaign, there is cost . Also, if it is in the form of a campaign, it will be a lot of work for us. If it is means to be provided every month, most of the time it is done with health extension workers which cause overlap of activities. If it has payment the cost would be difficult.

4. What are the facilitators to provide the service?

**Answer** ፡ In our side there is no any facilitator, but those who come to do research at the health post call mothers of adolescents having developmental disability and teach them to prevent knowledge gap. They teach the mothers to take out their children. Most of the time they hide their children. other than this there is no other facilitator. They were teaching that they can be what their peers can, and. they can go to school. Just don't hide.

**5.Do you give treatment once you identify developmental disability**

**Answer: No**

**Thank you**
