# Supplementary material for: Why are the implementation of and access to child developmental assessment within routine healthcare so difficult in Ethiopia? A descriptive qualitative study
Source: PLoS One. 2026 Aug 10;21(8):e0355686. doi: 10.1371/journal.pone.0355686 (PMC13456246; doi:10.1371/journal.pone.0355686)
Supplement: Supplementary file 2 [file pone.0355686.s002.zip › data/Transcripit 9.docx]

**Transcript 9**

1. How is child development monitoring being implemented in this health facility?

Answer: When the children come to us for treatment, we start with general examination. Then we see for child development, if they have a delay based on the standard Milestone we have , we will tell mothers how to follow up if there are babies who are not making it. There are no children we will leave without seeing them.

1.1.is there any standard to classify the child on there developmental concern?

Answer: yes, there is Milestone. There is also a chart posted on the wall. It is also included in IMNCH, and EDD means that if we found out children whose family has concern, then we classify by using it whether the child has or no problem. We use it to see all children.

1.2. Do you examine all children or only for those who have a concern?

Answer: We mainly see children who have concerns specially those who are malnourished, and we do something special for them. A child should go in line with their age that means children should do what is expected from there age . If this child fail to do that , we will provide special supervision. We also advise the mother to play with them and tell them about 85% of their child ‘s mental development is between 1 year to 3 years. When we put them on OTP, we tell them it is not enough to just give the baby plapy nut. While we send her home we tell her to let the child play, talk to him and the food he eat will benefit him if we do this things together.

2. How do you involve care givers or families in the developmental assessment? Because you told me you initiate the developmental assessment based on the family concern?

Answer: Family often does not have awareness. Most of them come for treatment. we start by taking weight then we'll ask them why they came, then we'll see what need to be seen next. Then we ask her starting from pregnancy during ANC follow up. We ask her when the baby was born, whether she give birth at home or not. We do this for children who has concerns about. After this we ask, what is expected from the child at this his age . For example, a 1-year-old child is supposed to stand on something and this exists on the milestone . If he does not full feel this she tell us as my child could not sit or stand. Its us who ask them this things but there are no mothers who come to us by complaining my child is lagging behind his friends.

If the child doesn't meet the criteria there, there is something we can suspected then refer to a hospital, but I haven't personally experienced it. But if there is a baby that I suspected, I will council the mother and appoint her for follow up and told her to come back and contact me at that time.

2.1. Do you have any schedule for follow up?

Answer: For all children coming to the clinics they have a follow up. There are kids we appoint after two days, five days or a week. But often they have problem for coming back. They have an appointment but they don't come. If the child gets better, they will stay at home, but we will appoint them anyways.

Do you do this to check for there development or for any case?

Answer: We don't appoint them for checkup. They will not come back. We often appoint mothers who come for treatment. If there is a developmental delay suspect, we will appoint them for same follow-up. Few of them come.

How do you link children with developmental delay? Would you please tell me this process?

Answer: Now, for example, when a baby comes to the clinic, if he is suspected, we look at the baby's age first. Then we look back at the expected milestone. if he does not meet the milestone he fall into the red column of the chart. we will send him to the hospital for treatment because if he does not meet the two milestones he has the problem. There is a referral system.

3. Okay still now we have been discussing how the assessment is being implemented, would you please tell me what the barriers are to provide the assessment?

Answer: you saw our health center, and the set up is not suitable for under five clinic. EPI service is being provided together with it. For some children, for example, there is something called a play box station and it is not suitable for implementing that. Official from the region came with some items, but we don't even have a shelf to store the items. There is a real challenge in doing it right.

Regarding skill there are trained professionals but the other trained professionals are not working here, they are in other OPDs. I am working here with others who are not trained.

I don’t think the training is sufficient because if we see as a country during the training period what was suggested was to do EDD in a well organized way starting from ANC. It was mentioned a child can start to differentiate things at 6 month in abdomen , to identify maternal or other voices at the time. EDD should be started from that time. we tell the mothers the child will become effective ,productive and do not be found at useless in the future if you do this assessment. But tell them verbally in the absence of full filled environment. We do not even have a place to show how to do the assessment. So that's the challenge.

3.1. what are the health system barrier ? Do you think it got sufficient focus/attention?

Answer: I don’t think that it has got sufficient attention. Because the government has decided to work on EDD now, after many researches. But the ministry of health has not done much on it. If Just because one person is being trained on EDD doesn't mean he can change this whole individuals. Therefore, I would like to say that all the health professionals should be trained and also this training should be given to the community level for health extension workers. Because I believe that it should be established from the lower level. To make the service successful, the work should start from the lowest level of health system.

3.2. Earlier in our conversation you have mentioned many barriers like training, playing stations, and etc. Would you please explain how the case flow would be the barrier for assessment and also the possible solution for that?

Answer: It's not something that takes any time. You tell the mothers and they will listen to you. We tell mothers especially when they come, we are very happy especially when we get under 3 years old, because it has to be done starting from the young age. We counsel them like this, but at that time, if there is at least material and a prepared place, we will do the examination and have our conversation while they are at waiting place. It should be done while they are playing, adapt the environment, when they are happy and their mind is not stressed. But we are not doing that. But when they come in, we tell them, we do counseling and there is nothing to be done. no time is wasted, because if a case comes to me, I will tell her either at the beginning or the end about EDD. Other wise its not time taking.

I have told you before that if there is something convenient to keep even the items I took like shelf, , something that can be locked. Apart from that, it would be nice if there was a place where the children could sit until they came in from outside. We talked to him ( the manager ) but no one is willing to work. It is good if a place is prepared.

I used to ask him repeatedly. Even the color inside the room was painted after insisting for a long period. It is not favorable environment and everyone is not cooperative. everyone should involve and be trained on EDD, that's why.

3.3. What do you think the possible solution for this things are?

Answer: As I said, the responsibility should not only be from the higher leader to the health center. It would be good if it included all people so that everyone share their own responsibility. I believe that during the EDD training time if the manager of health center had gone and take it, he would have executed some things saying that it is my business. I believe that if the concerned body gets involved and then extends down to extension workers. I think everyone should be able to take the responsibility equally.

3.4. What do you think about the mode of delivering the assessment? Do you think what you are doing now is sufficient or do you have any other suggestion ?

Answer: It's known that its difficult economically, I don't think it's possible for everyone to make digital assessment. If the setup is fixed enough with what we have, it is better to put it graphically. because it is easy to use pictures. sometimes it is good to use digital like using LCD at the waiting area. It would be good to display a small video about EDD by LCD and making role play .so that mothers have a better awareness and I suggested working starting from that level. That is before they even enter the health facility.

4. What are the facilitators to provide the service?

Answer: I mean, after I trained it makes me happy that It's not something we spend a lot of money on, and by talking and paying attention to children and listening to them, we don't have to spend any energy because if we work on those children, where can this country reach in the future? That means we are the one to work on child mind. It is our responsibility to make them good citizens. What I feel while assessing is that if we do this, they will be creative and talented in the future. I convince my self to work on this when I came.

5.How do you manage the materials that you are donated for assessment?

Answer: To put the item, the item is sited since it comes. It has been 1 month or 2 months. Its in store and hopefully this will be solved.

How long has it been since you took the training? Less than 1 year.

6.Have you examined many children so far in one year?

Answer: we mainly examine malnourished children

7. Do you think that the assessment is being given now sufficient? What do you think of changing the service with a routine screening? What would be the possible challenge?

Answer: The service being provided currently is not sufficient. The children come to the health facility by some coincidence it can be even once a year.AS we have discussed earlier it did not get attention like other diseases. The reason is it should be worked on this children. If we have to work on children the time is now .I believe that this thing should get attention. The service provided now is not enough Because we get the children may be once a year or by coincidence.

7.1. what do you think of using a standard screening tool?

Answer: I think it would be very helpful and make our work very easy.

But there should be a well trained man power and the number of trained man power should be many. In addition, the set up should be adjusted, if we want to provide the service.

I have been telling you training for all professionals. This is because if they do not get the training, they became reluctant even if you give them the orientation. They keep saying she got the money from the training that is why she is acting like this and why should we care. So the training should be inclusive.

8. Finally is there a long term follow up for children having developmental delay in this facility?

Answer: This is economically difficult . most of the children who visit our facility are from lowest economical family. We could not do anything by ourself. we do nothing for them. If the child is under OTP we may support him ,while the child is under OTP we can do the EDD and follow both the malnutrition and the development. Otherwise we do not have anything to do economically.

What about the long term treatment?

Answer: I don’t know.

Thank you
